# Synthesis and Bioactivity of Luffarin I

**DOI:** 10.3390/md13042407

**Published:** 2015-04-20

**Authors:** Aitor Urosa, Isidro S. Marcos, David Díez, Anna Lithgow, Gabriela B. Plata, José M. Padrón, Pilar Basabe

**Affiliations:** 1 Department of Organic Chemistry, Chemistry Faculty, University of Salamanca, Plaza de los Caídos 1-5, 37008 Salamanca, Spain; E-Mails: aitor_ug@usal.es (A.U.); ismarcos@usal.es (I.S.M.); ddm@usal.es (D.D.); 2 Nuclear Magnetic Resonance Service, University of Salamanca, Plaza de los Caídos 1-5, 37008 Salamanca, Spain; E-Mail: tille1962@usal.es; 3 BioLab, University Institute of Bio-Organic “Antonio González” (IUBO-AG), Centre of Biomedicine Research of Canarias (CIBICAN), University of La Laguna, C/Astrofísico Francisco Sánchez 2, 38206 La Laguna, Spain; E-Mails: g.b.plata@gmail.com (G.B.P.); jmpadron@ull.es (J.M.P.)

**Keywords:** luffarin I, sclareol, diastereoselective reduction, sponges, sesterterpenolide, marine metabolites

## Abstract

The first synthesis of Luffarin I, sesterterpenolide isolated from sponge *Luffariella geometrica*, has been accomplished from commercially available sclareol. The key strategy involved in this synthesis is the diastereoselective reduction of an intermediate ketone. Luffarin I against human solid tumor cell lines showed antiproliferative activities (GI_50_) in the range 12–17 μM.

## 1. Introduction

During the last few years, there has been intensive research for new natural pharmacologically active compounds. In general, the chemistry of marine organisms and of sponges in particular has led to the discovery of a great number of novel and interesting metabolites [1]. Many marine-living organisms have developed toxic secondary metabolites to defend themselves against predators [2].

There is a group of pentaprenyl terpenoids whose structures are derivable from geranylfarnesyl diphosphate, known as sestertepenoids, of frequent occurrence in marine sponges. The diverse bioactivity of sesterterpenoids has made them attractive targets for both biomedical and synthetic purposes [3].

Marine sponges have been the source of a large number of relevant sesterterpenes with biological activities, including anti-feedant [4,5,6], platelet-aggregation inhibition [7,8] and anti-inflammatory [9,10].

**Figure 1:**
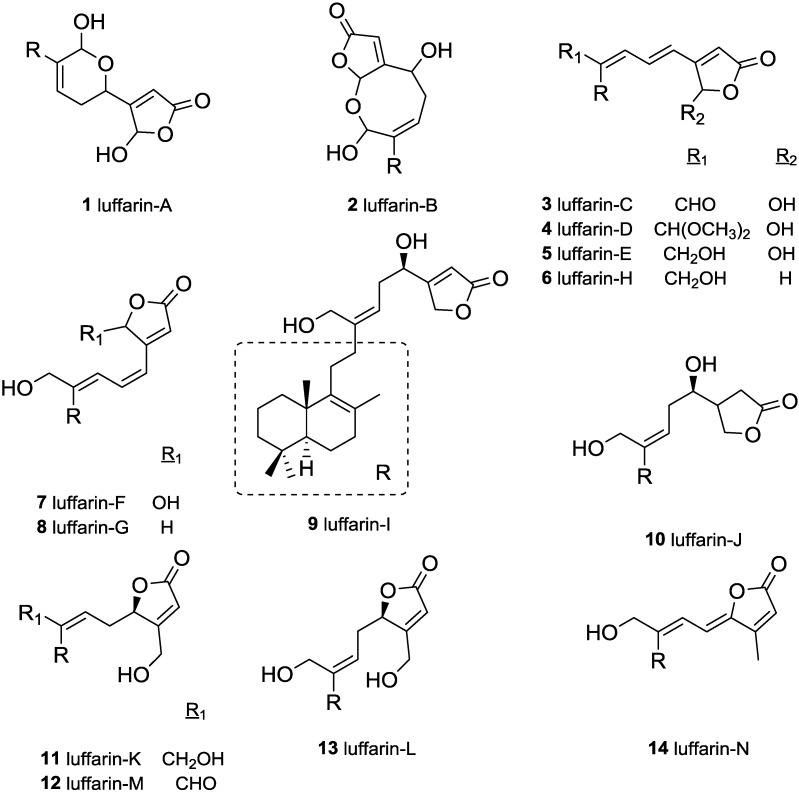
Structure of Luffarins (A–N) isolated from *Luffariella geometrica*.

**Figure 2:**
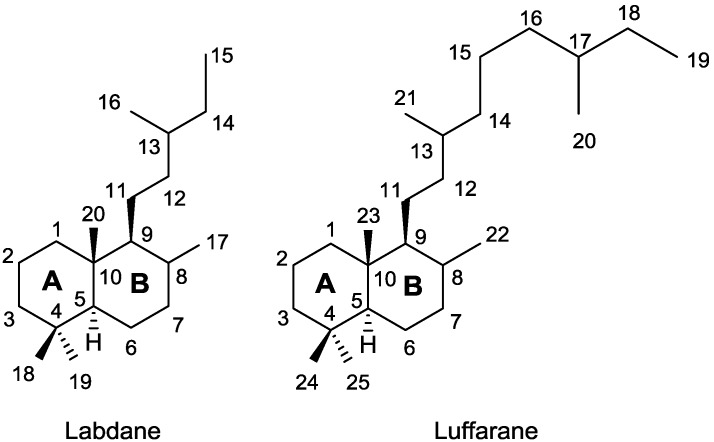
Labdane and luffarane skeletons numbering.

The luffarins (A–N), **1**–**14**, Figure 1, were isolated by Butler and Capon from an Australian marine sponge, *Luffariella geometrica* [11]. Luffarins are sesterterpenolides that have in common with the labdane skeleton the decaline moiety, showing the same stereochemistry for A and B rings. In particular, luffarins bear an eleven carbon atom side chain attached to C-9 with either a butenolide, a hydroxybutenolide or butanolide group, Figure 2. All of them have the same fragment R (Figure 1) and the skeleton can be defined as luffarane.

Luffarin I, **9**, can be proposed as a key intermediate for the synthesis of some luffarins. Herein we report the first synthesis and biological evaluation of luffarin I. Another sesterterpenolide with luffarane skeleton, luffalactone, has been synthesized previously [12].

## 2. Results and Discussion

**Scheme 1:**
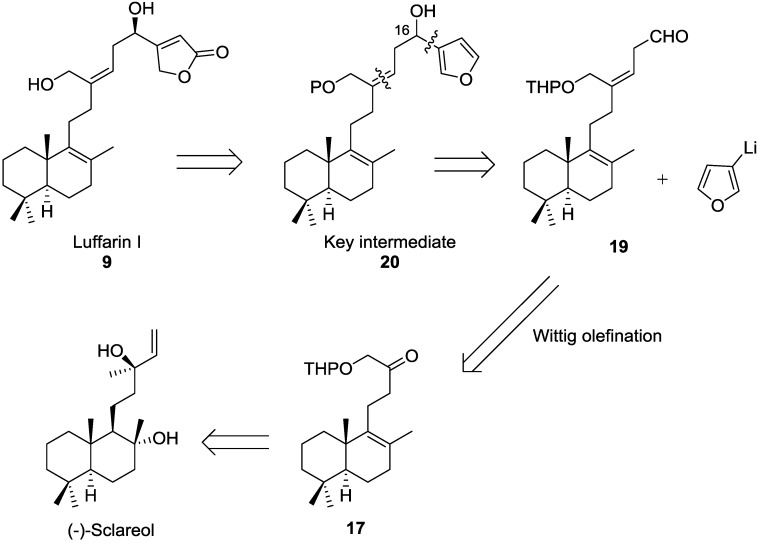
Retrosynthetic analysis for Luffarin I, **9**.

Our retrosynthetic analysis for luffarin I, **9**, is outlined in Scheme 1.

The synthesis of Luffarin I, could proceed from the furane intermediate **20**, that can act as key intermediate in the synthesis. The furane ring can be added to the side chain of an aldehyde as **19**, by an organometallic addition. The side chain of **19** can be obtained by a Wittig olefination of a methyl ketone **17**. The last compound can be obtained from (−)-sclareol, as a starting material (Scheme 1).

**Scheme 2:**
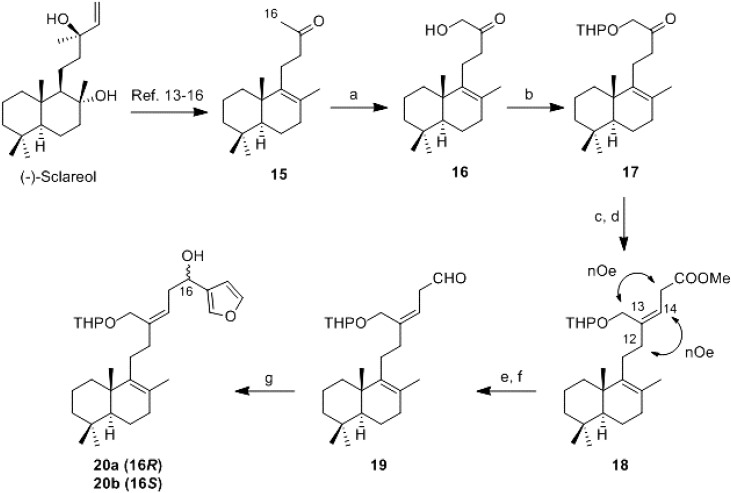
Synthesis of **20a/20b**. *Reagents and conditions:* (**a**) i: (Diacetoxyiodo) benzene (DIB), KOH/MeOH, 0 °C. ii: H_2_SO_4_ 5%, 0 °C, 1.5 h, 50%; (**b**) Dihydropyran (DHP), *p*TsOH, benzene, RT, 100%; (**c**) (2-carboxyethyl)triphenylphosphonium bromide, *n*-BuLi, THF/DMSO, −5 °C; (**d**) TMSCHN_2_, benzene/MeOH, 0 °C, 10 min, 56%; (**e**) LiAlH_4_, Et_2_O, 0 °C, 0.25 h, 100%; (**f**) Dess-Martin Periodinane (DMP), DCM, RT, 0.5 h, 100%; and (**g**) 3-bromofurane, *n*-BuLi, Et_2_O, −78 °C, 0.5 h, 41%.

Keeping in mind the retrosynthetic scheme, methylketone **15** was obtained by the degradation of (−)-sclareol using known procedures [13,14,15,16] (Scheme 2). The functionalization of methyl at C-16 can be achieved in two ways (1) in two steps with lead tetraacetate (LTA), BF_3_•Et_2_O [17] and subsequent hydrolysis of the acetoxy group (40% in two reactions) or (2) by direct (diacetoxyiodo)benzene (DIB) [18,19] functionalization with more favorable results, giving in both cases **16** (Scheme 2). After protection of the primary hydroxyl group as its tetrahydropyranyl derivative, **17**, Wittig reaction [20,21,22,23,24] with (2-carboxyethyl)triphenylphosphonium bromide and subsequent esterification of the resulting acid with trimethylsilyldiazomethane (TMSCHN_2_) achieved methyl ester **18**. NOE experiments confirmed the *Z*-geometry of double bond ∆^13^.

Ester **18**, was reduced with lithium aluminium hydride (LAH) and the resulting alcohol was oxidized using Dess-Martin [25,26,27] procedure to give aldehyde **19**.

The 3-bromofurane lithium derivative, achieved by metallation of 3-bromofurane with *n*-BuLi, was added to aldehyde **19** giving a 1:1 mixture of epimers at C-16 (**20a/20b**).

**Scheme 3:**
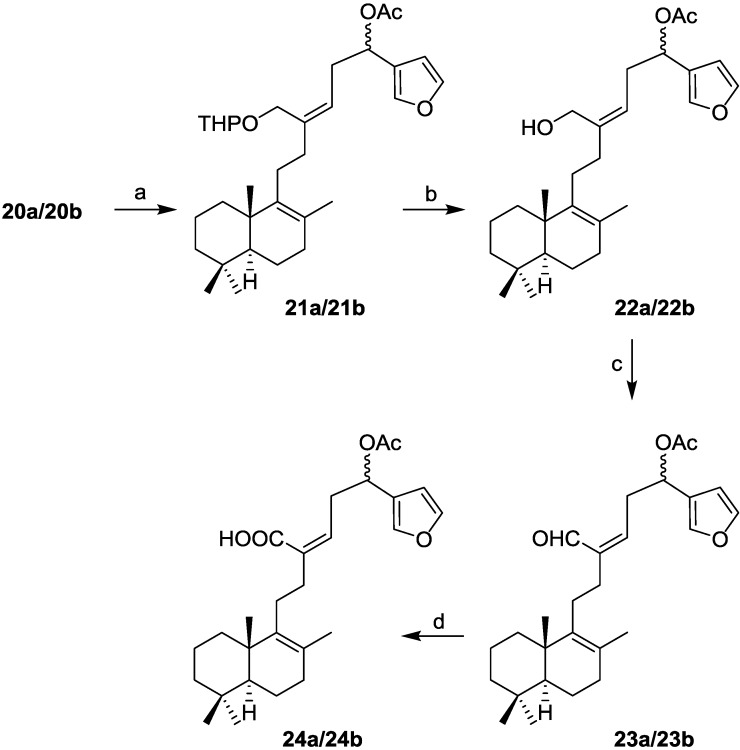
Synthesis of **24a/24b**. *Reagents and conditions:* (**a**) Ac_2_O, pyridine, RT, 24 h, 99%; (**b**) *p*-TsOH, MeOH, RT, 4 h, 100%; (**c**) DMP, DCM, RT, 0.5 h, 99%; (**d**) NaClO_2_ 5%, NaH_2_PO_4_, 2-methyl-2-butene, RT, 0.5 h, 99%.

The oxidation of the mixture **20a**/**20b** with tetrapropylammonium perruthenate (TPAP) in presence of 4-methylmorpholine *N*-oxide (NMO) led only to decomposition products. Thus, before oxidation of the C-16 hydroxyl group, the double bond was deactivated by conjugation with a carboxylic acid (Scheme 3). Acetoxylation of the secondary alcohol led to the acetoxy derivatives **21a/21b**, which by deprotection of the THP group, gave the hydroxyderivatives **22a/22b**. The oxidation of the later compounds to the required acids was achieved in two steps; oxidation of the alcohols to aldehydes **23a/23b** was carried out with DMP and finally oxidation to the desired acids mixture **24a/24b** by oxidation of the aldehydes with sodium chlorite (Scheme 3).

**Scheme 4:**
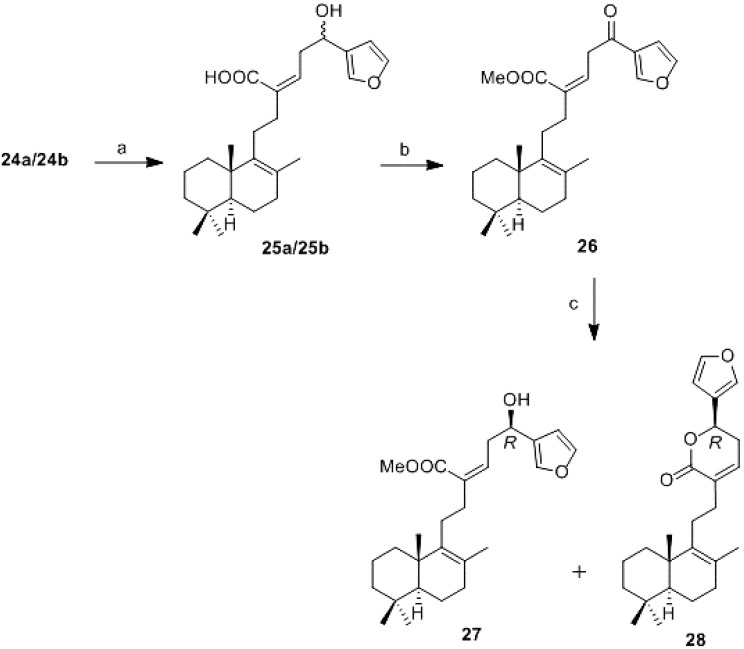
Synthesis of **27** and **28**. *Reagents and conditions:* (**a**) K_2_CO_3_, MeOH, RT, 7 h, 100%; (**b**) i: DMP, DCM, 0 °C, 0.5 h. ii: TMSCHN_2_, MeOH/benzene, 0 °C, 10 min. 89% for two steps; (**c**) (*S*)-2-methyl-CBS-oxazaborolidene, Me_2_S.BH_3_, toluene, −78 °C to −30 °C, 20 h, (**27**, 52%, **28**, 42%).

The resulting α,β-unsaturated acids **24a/24b** were transformed into ketone **26** by hydrolysis of the acetoxy group and oxidation of the resulting alcohol (**25a**/**25b**) with DMP [25,26,27] at low temperature and subsequent esterification of the carboxylic group with TMSCHN_2_ (Scheme 4). The Corey-Bakshi-Shibata [28,29,30,31,32,33] reduction of ketone **26** allows obtaining diastereoselectively only one of the C-16 hydroxyl derivatives (Scheme 4).

Treatment of **26** with (*S*)-2-methyl-Corey-Bakshi-Shibata-oxazaborolidine using borane dimethyl sulfide as reducing agent under inert atmosphere and low temperature, produced diastereoselectively **27** and **28**. The reduction proceeds with an excellent yield and both **27** and **28** are valid intermediates in the synthesis of the target molecule luffarin I.

The generated stereogenic center at C-16 has *R* configuration as expected, confirmed the application of Mosher methodology [34,35,36,37,38,39], see Supplementary Information. 

**Scheme 5:**
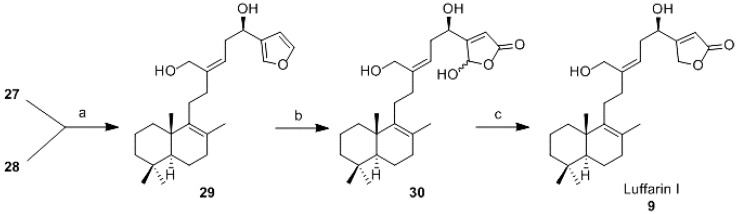
Synthesis of luffarin I (**9**). *Reagents and conditions:* (**a**) DIBAL-H, DCM, RT, 1.5 h, (from **27**, 85%; from **28**, 95%); (**b**) O_2_, *N,N*-diisopropylethylamine (DIPEA), hυ, Bengal Rose, −78 °C, 6 h, 99%; (**c**) NaBH_4_, EtOH, 0 °C, 5 min, 71%.

Reduction of either **27** or **28** with diisobutylaluminium hydride (DIBAL-H) afforded diol **29**, in good yield in both cases. Conversion of the furane ring of **29**, into the γ-hydroxybutenolide was carried out following Faulkner’s methodology [40]. Photochemical oxidation of **29** with ^1^O_2_ in the presence of Rose Bengal irradiating with a 200W lamp for 10 min gave quantitatively the hydroxybutenolide **30**. Reduction of **30** with NaBH_4_ [41] transformed the γ-hydroxybutenolide ring into the required γ-butenolide present in luffarin I, **9** (Scheme 5).

The spectroscopic data of **9**, as well as its optical rotation ${\left[\text{α}\right]}_{\text{D}}^{20}$ = +69.0 (*c* 0.51, CHC_l3_) comply with those corresponding to the natural product described by Butler and Capon as luffarin I ${\left[\text{α}\right]}_{\text{D}}^{20}$ = +64.3 (*c* 1.4, CHCl_3_) [11]. It can be concluded that luffarin I has been obtained from methylketone **15**, in 15 steps.

### Biological Studies

From the set of synthesized analogues, a total of four compounds were submitted to biological assays. The *in vitro* activity was assessed in A549, HBL-100, HeLa, SW1573, T-47D and WiDr human solid tumor cells. The results expressed as GI_50_ were obtained using the SRB assay [42], and the results are given in Table 1. The standard anticancer drugs cisplatin and etoposide were used as positive controls. Overall, the data on antiproliferative activity show that all tested compounds exhibited growth inhibition in at least four of the cell lines of the panel. The natural compound **9** is the most active of the series with GI_50_ values in the range 12–17 µM. Table 1. The antiproliferative activity is comparable to the reference drugs in the most resistant cell lines T-47D and WiDr. Although the set of compounds in this study is small, the presence of the butenolide fragment can explain the enhanced activity of **9** when compared to analogues **29** and **30**.

**Table 1:** Antiproliferative activity (GI_50_) against human solid tumor cells of compounds produced via Scheme 4 and Scheme 5. Values are given in μM and are means of three to five experiments; standard deviation is given in parentheses. n.d. = not determined.

Compound	Cell Line					
	A549 (lung)	HBL-100 (breast)	HeLa (cervix)	SW1573 (lung)	T-47D (breast)	WiDr (colon)
**9**	12 (±0.6)	15 (±0.6)	13 (±1.4)	13 (±0.8)	17 (±0.9)	17 (±0.7)
**28**	24 (±0.6)	41 (±1.9)	30 (±2.1)	37 (±2.5)	55 (±3.0)	52 (±0.6)
**29**	57 (±9.3)	93 (±13)	43 (±9.6)	64 (±12)	>100	>100
**30**	32 (±2.3)	27 (±3.5)	25 (±3.2)	26 (±2.4)	35 (±2.4)	54 (±8.7)
cisplatin	n.d.	1.9 (±0.2)	2.0 (±0.3)	3.0 (±0.4)	15 (±2.3)	26 (±5.3)
etoposide	n.d.	1.4 (±0.1)	3.3 (±1.6)	15 (±1.5)	22 (±5.5)	23 (±3.1)

## 3. Experimental Section 

16-Hydroxy-14,15-dinor-labd-8-en-13-one (**16**): To a stirred solution of **15** (60 mg, 0.23 mmol) in MeOH (1.1 mL) was added slowly a solution of KOH (76 mg, 1.15 mmol) in MeOH (1.75 mL) and the mixture was reacted at 0 °C for 10 min. Afterwards, (diacetoxyiodo)benzene (DIB) (146 mg, 0.46 mmol) was added, and the mixture was stirred at 0 °C following the reaction evolution by TLC. When the reaction had finished, a 5% aqueous solution of H2SO4 (1.5 mL) was added and the mixture was reacted at 0 °C for 90 min. It was quenched with water and the product was extracted with DCM. The combined organic layers were washed with brine, dried (Na2SO4), filtered, and concentrated *in vacuo*. The resulting crude residue was purified by flash CC (hexane-AcOEt, 98:2) to obtain **16** (33 mg, 50%). ${\left[\text{α}\right]}_{\text{D}}^{20}$ = +81.9 (*c* 0.64, CHCl_3_); IR υ 3443 (OH), 2936, 1721 (C=O), 1441, 1375, 1024; ^1^H-NMR (400 MHz, CDCl_3_) δ 4.24 (2H, s, H-16), 2.50–2.30 (4H, m, H-11, H-12), 2.00–1.00 (11H, m), 1.57 (3H, s, Me-17), 0.94 (3H, s, Me-20), 0.88 (3H, s, Me-18), 0.82 (3H, s, Me-19); ^13^C-NMR (100 MHz, CDCl_3_) δ 209.6 (C), 138.7 (C), 127.3 (C), 67.9 (CH_2_), 51.9 (CH), 41.7 (CH_2_), 39.3 (CH_2_), 39.0 (C), 37.0 (CH_2_), 33.6 (CH_2_), 33.3 (C, CH_3_), 21.6 (CH_3_), 21.4 (CH_2_), 19.9 (CH_3_), 19.4 (CH_3_), 18.9 (CH_2_-2).

16-(2-Tetrahydropyranyloxy)-14,15-dinor-labd-8-en-13-one (**17**): To a stirred solution of **16** (152 mg, 0.54 mmol) in dry benzene (3.6 mL) was added *p*-toluenesulfonic acid (3 mg, 0.016 mmol) and dihydropyran (DHP) (0.15 mL, 1.62 mmol). The evolution of reaction was controlled by TLC. When the reaction had finished a 10% aqueous solution of Na2CO3 (3 mL) was added and it was reacted for 30 min. It was quenched with water and the product was extract with AcOEt. The combined organic layers were washed with water and brine, dried (Na2SO4), filtered, and concentrated *in vacuo* to obtain **17** (196 mg, 100%). ${\left[\text{α}\right]}_{\text{D}}^{20}$ = +26.0 (*c* 2.4, CHCl_3_); IR υ 2941, 1717 (C=O), 1665 (C=C), 1456, 1126, 1036; ^1^H-NMR (400 MHz, CDCl_3_) δ 4.94 (1H, dd, *J* = 2.8 and 4.8 Hz, H-2′ major.), 4.63 (1H, t, *J* = 3.7 Hz, H-2′ minor.), 4.23 (1H, d, *J* = 17 Hz, H_A_-16), 4.09 (1H, d, *J* = 17 Hz, H_B_-16), 3.90–3.40 (2H, m, H-6′), 2.60–2.50 (2H, m, H-12), 2.40–2.10 (2H, m, H-11), 2.00–1.00 (17H, m), 1.53 (3H, s, Me-17), 0.93 (3H, s, Me-20), 0.87 (3H, s, Me-18), 0.82 (3H, s, Me-19); ^13^C-NMR (100 MHz, CDCl_3_) δ 208.8 (C), 139.2 (C), 126.7 (C), 98.9/94.6 (CH), 71.9 (CH_2_), 62.9/62.4 (CH_2_), 51.9 (CH), 41.7 (CH_2_), 39.9 (CH_2_), 39.0 (C), 36.9 (CH_2_), 33.6 (CH_2_), 33.2 (C, CH_3_), 30.6/30.2 (CH_2_), 25.4/25.2 (CH_2_), 21.6 (CH_3_), 21.3 (CH_2_), 19.9 (CH_3_), 19.7/19.2 (CH_2_), 19.4 (CH_3_), 18.9 (CH_2_-2); HRMS (ESI) *m/z* calcd for C_23_H_38_O_3_Na (M + Na)^+^ 385.2713, found 385.2722.

Methyl 21-(2-tetrahydropyranyloxy)-17,18,19,20-tetranor-luffara-8,13*Z*-dien-16-oate (**18**): To a suspension of (2-carboxyethyl)triphenylphosphonium bromide (270 mg, 0.65 mmol) in dry THF (3.2 mL) and dry DMSO (0.8 mL) at −5 °C under argon atmosphere, *n*-BuLi (1.6 M in hexane; 0.8 mL, 1.3 mmol) was added slowly and the reaction was stirred for 10 min. A solution of **17** (47 mg, 0.13 mmol) in THF/DMSO 4:1 (2.5 mL) was added via cannula dropwise and the reaction was stirred for 90 min. It was allowed to warm to room temperature, quenched with saturated aqueous solution of NH4Cl and extracted with AcOEt. The combined organic layers were washed with water and brine, dried (Na2SO4), filtered, and concentrated *in vacuo*. The obtained acid was directly esterified: the resulting crude residue was dissolved in C6H6/MeOH 1:1 (2.4 mL) and cooled at 0 °C. Under argon atmosphere TMSCHN2 (2.0 M in hexane; 0.3 mL, 0.6 mmol) was added. After 10 min, the solvent was removed under reduced pressure and the resulting crude residue was purified by CC (hexane-AcOEt, 97:3) to obtain **18** (30 mg, 54%). ${\left[\text{α}\right]}_{\text{D}}^{20}$ = +41.4 (*c* 0.8, CHCl_3_); IR υ 2941, 2868, 1744 (C=O), 1200, 1132, 1024; ^1^H-NMR (400 MHz, CDCl_3_) δ 5.58 (1H, t, *J* = 7.1 Hz, H-14), 4.58 (1H, t, *J* = 3.0 Hz, H-2′), 4.21 (1H, dd, *J* = 11.9 Hz, H_A_-21), 4.04 (1H, dd, *J* = 2.1 and 11.9 Hz, H_B_-21), 3.90–3.80 (1H, m, H-6′), 3.68 (3H, s, COOMe), 3.50–3.45 (1H, m, H-6′), 3.17 (2H, d, *J* = 7.1 Hz, H-15), 2.20–2.05 (4H, m, H-12, H-11), 1.95–1.10 (17H, m), 1.57 (3H, s, Me-22), 0.93 (3H, s Me-23), 0.88 (3H, s, Me-25), 0.82 (3H, s, Me-24); ^13^C-NMR (100 MHz, CDCl_3_) δ 172.5 (C), 140.2 (C), 136.8 (C), 126.0 (C), 119.5 (CH), 97.6/94.6 (CH), 64.3 (CH_2_), 62.9/62.1 (CH_2_), 51.9 (CH), 51.7 (CH_3_), 41.8 (CH_2_), 39.0 (C), 36.9 (CH_2_), 36.0 (CH_2_), 33.6 (CH_2_), 33.3 (C, CH_3_), 33.1 (CH_2_), 30.6/30.5 (CH_2_), 27.1 (CH_2_), 25.4 (CH_2_), 21.7 (CH_3_), 20.0 (CH_3_), 19.7 (CH_2_), 19.5 (CH_3_), 19.0 (CH_2_ - 2); HRMS (ESI) *m/z* calcd for C_27_H_44_O_4_Na (M + Na)^+^ 455.3132, found 455.3116.

21-(2-Tetrahydropyranyloxy)-17,18,19,20-tetranor-luffara-8,13*Z*-dien-16-al (**19**): To a solution of **18** (31 mg, 0.072 mmol) in Et_2_O (5.3 mL) at 0 °C was added LiAlH_4_ (27 mg, 0.72 mmol). The reaction was stirred at rt for 15 min and then quenched with wet AcOEt, dried (Na_2_SO_4_), filtered through a short pad of Celite and concentrated *in vacuo*. The resulting alcohol (80 mg, 0.196 mmol) was solved in DCM (11.8 mL) and it was added DMP (103 mg, 0.25 mmol). The reaction was stirred at rt for 30 min. It was diluted with AcOEt and washed with 10% NaHCO_3_/10% Na_2_S_2_O_3_ 1:1, dried (Na_2_SO_4_), filtered, and concentrated *in vacuo* to obtain **19** (79 mg, 100% from **18**). IR υ 2940, 2725, 1726 (CHO), 1684 (C=C), 1456, 1375, 1119, 1024; ^1^H-NMR (400 MHz, CDCl_3_) δ 9.67 (1H, s, H-16), 5.57 (1H, t, *J* = 7.4 Hz, H-14), 4.60–4.57 (1H, m, H-2′), 4.23 (1H, d, *J* = 11.8 Hz, H_A_-21), 4.03 (1H, d, *J* = 11.8 Hz, H_B_-21), 3.89–3.49 (2H, m, H-6′), 3.27 (2H, d, *J* = 7,5 Hz, H-15), 2.13–2.05 (4H, m, H-11, H-12), 1.95–1.10 (17H, m, H-7), 1.58 (3H, s, Me-22), 0.94 (3H, s, Me-23), 0.88 (3H, s, Me-25), 0.83 (3H, s, Me-24).

21-(2-Tetrahydropyranyloxy)-19,20-epoxy-luffara-8,13*Z*,17(20),18-tetraen-16(*R*,*S*)-ol (**20a/20b**): To a solution of 3-bromofuran (0.13 mL, 1.47 mmol) in Et2O at −78 °C under argon atmosphere was added dropwise *n*-BuLi (1.6 M in hexane; 0.92 mL, 1.47 mmol) and the solution was stirred for 10 min. After that, a solution of **19** (59 mg, 0.147 mmol) in Et2O (1.6 mL) was added dropwise via cannula and the mixture was stirred for 30 min. It was allowed to warm to room temperature, quenched with a saturated aqueous solution of NH4Cl and extracted with AcOEt. The combined organic layers were washed with water and brine, dried (Na2SO4), filtered, and concentrated *in vacuo*. The resulting crude residue was purified by flash CC (hexane-AcOEt, 9:1) to obtain a mixture of **20a/20b** (27 mg, 41%). ${\left[\text{α}\right]}_{\text{D}}^{20}$ = +47.4 (*c* 0.22, CHCl_3_); IR υ 3249 (OH), 2940, 1440, 1202, 1024; ^1^H-NMR (400 MHz, CDCl_3_) δ 7.37 (2H, s, H-19, H-20), 6.38 (1H, s, H-18), 5.51–5.43 (1H, m, H-14), 4.71–4.64 (2H, m, H-2′, H-16), 4.17–3.90 (2H, m, H-21), 3.88–3.52 (2H, m, H-6′), 2.60–2.40 (2H, m, H-15), 2.20–2.10 (4H, m, H-11, H-12), 1.85–1.05 (17H, m), 1.58 (3H, s, Me-22), 0.94 (3H, s, Me-23), 0.88 (3H, s, Me-25), 0.83 (3H, s, Me-24); ^13^C-NMR (100 MHz, CDCl_3_) δ 143.1 (CH), 140.6 (C), 140.2 (C), 138.9/138.8 (CH), 129.2 (C), 126.0 (C), 124.7 (CH), 108.6 (CH), 97.9/97.2 (CH), 66.2 (CH), 64.6/64.2 (CH_2_), 61.9/61.7 (CH_2_), 51.9 (CH), 41.9 (CH_2_), 39.0 (C), 37.0 (CH_2_), 36.8 (CH_2_), 36.6 (CH_2_), 33.6 (CH_2_), 33.3 (C, CH_3_), 30.4/30.3 (CH_2_), 27.3 (CH_2_), 25.4 (CH_2_), 21.7 (CH_3_), 20.1 (CH_3_), 19.7 (CH_2_), 19.5 (CH_3_), 19.0 (CH_2_ - 2); HRMS (ESI) *m/z* calcd for C_30_H_46_O_4_Na (M + Na)^+^ 493.3288, found 493.3303.

16(*R*,*S*)-Acetoxy-21-(2-tetrahydropyranyloxy)-19,20-epoxi-luffara-8,13*Z*,17(20),18-tetraene (**21a/21b**): To a solution of **20a/20b** (30 mg, 0.064 mmol) in pyridine (1.5 mL) was added acetic anhydride (1.5 mL) and the reaction was stirred at rt in anhydrous conditions for 24 h. It was quenched with ice and extracted with AcOEt. The combined organic layers were washed with 2 M aqueous solution of HCl, 10% aqueous solution of NaHCO3 and water until neutral pH was reached, dried (Na2SO4), filtered, and concentrated *in vacuo* to obtain **21a/21b** (32 mg, 99%). ${\left[\text{α}\right]}_{\text{D}}^{20}$ = +19.6 (*c* 0.08, CHCl_3_); IR υ 2940, 1742 (C=O), 1371, 1236, 1024; ^1^H-NMR (400 MHz, CDCl_3_) δ 7.41 (1H, s, H-20), 7.37 (1H, s, H-19), 6.39 (1H, bs, H-18), 5.80–5.75 (1H, m, H-16), 5.31 (1H, t, *J* = 7.3 Hz, H-14), 4.60–4.50 (1H, m, H-2′), 4.25–3.95 (2H, m, H-21), 3.95–3.45 (2H, m, H-6′), 2.80–2.50 (2H, m, H-15), 2.20–2.10 (4H, m, H-11, H-12), 2.04 (3H, s, *Me*COO), 2.00–1.00 (17H, m), 1.57 (3H, s, Me-22), 0.93 (3H, s, Me-23), 0.88 (3H, s, Me-25), 0.82 (3H, s, Me-24); ^13^C-NMR (100 MHz, CDCl_3_) δ 170.3 (C), 143.1 (CH), 140.3 (C, CH), 140.2 (C), 126.0 (C), 123.5 (C), 122.5 (CH), 109.0 (CH), 97.8/97.6 (CH), 68.2 (CH), 64.2 (CH_2_), 62.1/61.9 (CH_2_), 51.9 (CH), 41.8 (CH_2_), 39.0 (C), 36.9 (CH_2_), 36.1 (CH_2_), 33.6 (CH_2_), 33.3 (C, CH_3_), 33.1 (CH_2_), 30.6 (CH_2_), 27.4 (CH_2_), 25.4 (CH_2_), 21.7 (CH_3_), 21.2 (CH_3_), 20.1 (CH_3_), 19.5 (CH_3_), 19.3 (CH_2_), 19.0 (CH_2_ - 2); HRMS (ESI) *m/z* calcd for C_32_H_48_O_5_Na (M + Na)^+^ 535.3394, found: 535.3381.

16(*R*,*S*)-Acetoxy-19,20-epoxy-luffara-8,13*Z*,17(20),18-tetraen-21-ol (**22a/22b**): To a solution of **21a/21b** (65 mg, 0.13 mmol) in MeOH (12.2 mL) was added *p*-toluenesulfonic acid (8 mg, 0.04 mmol) and the reaction was stirred at rt for 4 h. It was added water and extracted with AcOEt. The combined organic layers were washed with water and brine, dried (Na2SO4), filtered, and concentrated *in vacuo* to afford **22a/22b** (56 mg, 100%). ${\left[\text{α}\right]}_{\text{D}}^{20}$ = +8.9 (*c* 0.17, CHCl_3_); IR υ 3468 (OH), 3134, 2928, 1739 (C=O), 1371, 1238, 1024; ^1^H-NMR (400 MHz, CDCl_3_) δ 7.41 (1H, s, H-20), 7.37 (1H, s, H-19), 6.39 (1H, bs, H-18), 5.80–5.75 (1H, m, H-16), 5.31 (1H, t, *J* = 7.3 Hz, H-14), 4.12 (1H, d, *J* = 14.0 Hz, H_A_-21), 4.11 (1H, d, *J* = 14.0 Hz, H_B_-21), 2.80–2.50 (2H, m, H-15), 2.20–2.10 (4H, m, H-11, H-12), 2.04 (3H, s, *Me*COO), 2.00–1.00 (11H, m), 1.57 (3H, s, Me-22), 0.93 (3H, s, Me-23), 0.88 (3H, s, Me-25), 0.82 (3H, s, Me-24); ^13^C-NMR (100 MHz, CDCl_3_) δ 170.3 (C), 143.3 (CH), 142.9 (C), 140.3 (C, CH), 126.0 (C), 124.1 (C), 121.8 (CH), 108.8 (CH), 68.3 (CH), 60.1 (CH_2_), 51.8 (CH), 41.7 (CH_2_), 38.9 (C), 36.9 (CH_2_), 36.3 (CH_2_), 33.6 (CH_2_), 33.3 (C, CH_3_), 33.1 (CH_2_), 27.3 (CH_2_), 21.7 (CH_3_), 21.2 (CH_3_), 20.1 (CH_3_), 19.5 (CH_3_), 19.0 (CH_2_-2); HRMS (ESI) *m/z* calcd for C_27_H_40_O_4_Na (M + Na)^+^ 451.2819, found 451.2804.

16-(*R*,*S*)-Acetoxy-19,20-epoxy-luffara-8,13*Z*,17(20),18-tetraen-21-al (**23a/23b**): To a solution of **22a/22b** (10 mg, 0.025 mmol) in DCM (1.4 mL) was added DMP (13 mg, 0.05 mmol). The reaction was stirred at rt for 30 min. It was added AcOEt and washed with 10% NaHCO3/10% Na2S2O3 1:1, dried (Na2SO4), filtered, and concentrated *in vacuo* to obtain **23a/23b** (11 mg, 99%). ${\left[\text{α}\right]}_{\text{D}}^{20}$ = +22.2 (*c* 0.1, CHCl_3_); IR υ 3136, 2929, 2868 (CHO), 2733, 1741 (C=O), 1678 (C=C), 1371, 1234, 1024; ^1^H-NMR (400 MHz, CDCl_3_) δ 10.07 (1H, s, H-21), 7.43 (1H, s, H-20), 7.41 (1H, s, H-19), 6.40 (1H, bs, H-18), 6.38 (1H, t, *J* = 8.4 Hz, H-14), 5.91 (1H, t, *J* = 6.6 Hz, H-16), 3.22–2.95 (2H, m, H-15), 2.20–2.00 (4H, m, H-11, H-12), 2.05 (3H, s, *Me*COO), 2.00–1.00 (11H, m), 1.58 (3H, s, Me-22), 0.92 (3H, s, Me-23), 0.88 (3H, s, Me-25), 0.83 (3H, s, Me-24); ^13^C-NMR (100 MHz, CDCl_3_) δ 190.7 (C), 171.1 (C), 143.6 (CH), 143.4 (C), 141.3 (CH), 140.2 (CH), 139.8 (C), 126.7 (C), 123.8 (C), 108.6 (CH), 67.3 (CH), 51.9 (CH), 41.7 (CH_2_), 39.0 (C), 36.9 (CH_2_), 33.6 (CH_2_), 33.3 (C, CH_3_), 33.1 (CH_2_), 31.6 (CH_2_), 27.8 (CH_2_), 21.7 (CH_3_), 21.0 (CH_3_), 20.0 (CH_3_), 19.5 (CH_3_), 19.0 (CH_2_ - 2); HRMS (ESI) *m/z* calcd for C_27_H_38_O_4_Na (M + Na)^+^ 449.2662, found 449.2660.

16-(*R*,*S*)-Acetoxy-19,20-epoxy-luffara-8,13*Z*,17(20),18-tetraen-21-oic acid (**24a/24b**): To a solution of **23a/23b** (8 mg, 0.019 mmol) in *t*-BuOH (0.25 mL) and 2-methyl-2-butene (51 μL), a solution of monobasic sodium phosphate (NaH2PO4, 10 mg) in water (0.1 mL) and 5% aqueous solution of NaClO2 (48 μL) were added. The reaction mixture was stirred at rt for 30 min. Then, water and 2 M aqueous solution of HCl were added. It was extracted with AcOEt and the combined organic layers were washed with water until neutral pH was reached, dried (Na2SO4), filtered, and concentrated *in vacuo* to afford **24a/24b** (8 mg, 99%). ${\left[\text{α}\right]}_{\text{D}}^{20}$ = +31.4 (*c* 0.5, CHCl_3_); IR υ 3500–2700 (COOH), 2924, 2855, 1744 (C=O), 1694 (C=O), 1645 (C=C), 1456, 1371, 1234, 1024; ^1^H-NMR (400 MHz, CDCl_3_) δ 7.43 (1H, s, H-20), 7.38 (1H, s, H-19), 6.40 (1H, bs, H-18), 5.98 (1H, t, *J* = 6.9 Hz, H-14), 5.89 (1H, t, *J* = 7.0 Hz, H-16), 3.15–3.00 (2H, m, H-15), 2.35–2.20 (4H, m, H-11, H-12), 2.06 (3H, s, *Me*COO), 1.80–1.00 (11H, m), 1.56 (3H, s, Me-22), 0.92 (3H, s, Me-23), 0.87 (3H, s, Me-25), 0.82 (3H, s, Me-24); ^13^C-NMR (100 MHz, CDCl_3_) δ 171.8 (C), 170.1 (C), 143.1 (CH), 140.4 (CH), 139.3 (C), 138.3 (CH), 133.9 (C), 126.5 (C), 124.0 (C), 108.6 (CH), 67.8 (CH), 51.9 (CH), 41.8 (CH_2_), 39.0 (C), 36.9 (CH_2_), 35.1 (CH_2_), 34.5 (CH_2_), 33.6 (CH_2_), 33.3 (C, CH_3_), 27.8 (CH_2_), 21.7 (CH_3_), 21.1 (CH_3_), 20.1 (CH_3_), 19.5 (CH_3_), 19.0 (CH_2_-2); HRMS (ESI) *m/z* calcd for C_27_H_38_O_5_Na (M + Na)^+^ 465.2611, found 465.2604.

16-(*R*,*S*)-Hydroxy-19,20-epoxy-luffara-8,13*Z*,17(20),18-tetraen-21-oic acid (**25a/25b**): To a solution of **24a/24b** (20 mg, 0.05 mmol) in MeOH (1.4 mL) was added anhydrous K_2_CO_3_ (14 mg, 0.1 mmol) and the mixture was reacted at rt for 7 h. Then, water and 0.01M aqueous solution of HCl were added until neutral pH was reached. It was extracted with AcOEt and the combined organic layers were washed with water until neutral pH was reached and brine, dried (Na_2_SO_4_), filtered, and concentrated *in vacuo* to obtain **25a/25b** (20 mg, 100%). IR υ 3500–2700 (COOH), 2924, 2854, 1714 (C=O), 1456, 1377, 1261, 1026; ^1^H-NMR (400 MHz, CDCl_3_) δ 7.41 (1H, s, H-20), 7.40 (1H, s, H-19), 6.41 (1H, s, H-18), 6.08 (1H, t, *J* = 7.8 Hz, H-14), 4.85 (1H, t, *J* = 6.9 Hz, H-16), 2.95–2.85 (2H, m, H-15), 2.35–2.25 (4H, m, H-11, H-12), 2.05–1.00 (11H, m), 1.58 (3H, s, Me-22), 0.93 (3H, s, Me-23), 0.88 (3H, s, Me-25), 0.83 (3H, s, Me-24).

Methyl 16-oxo-19,20-epoxy-luffara-8,13*Z*,17(20),18-tetraen-21-oate (**26**): To a solution of **25a/25b** (11 mg, 0.027 mmol) in DCM (0.5 mL) at 0 °C was added DMP (21 mg, 0.054 mmol). The reaction mixture was stirred under argon atmosphere at rt for 30 min. Then, it was added AcOEt and the organic layer was washed with 10% aqueous solution of Na_2_S_2_O_3_, dried (Na_2_SO_4_), filtered, and the solvent was partially removed under reduced pressure. The obtained acid was esterified directly: the crude was dissolved in C_6_H_6_/MeOH 1:1 (0.34 mL) and cooled to 0 °C. Under argon atmosphere TMSCHN_2_ (2.0 M in hexane; 27 μL, 0.054 mmol) was added dropwise. After 15 min the solvent was removed under reduced pressure to obtain **26** (9 mg, 89%). ^1^H-NMR (400 MHz, CDCl_3_) δ 8.14 (1H, s, H-20), 7.44 (1H, s, H-18), 6.80 (1H, s, H-19), 6.39 (t, *J* = 6.8 Hz, H-14), 4.05 (2H, d, *J* = 6.8 Hz, H-15), 3.77 (3H, s, COO*Me*), 2.35–2.25 (4H, m, H-11, H-12), 2.05–1.00 (11H, m), 1.57 (3H, s, Me-22), 0.93 (3H, s, Me-23), 0.88 (3H, s, Me-25), 0.83 (3H, s, Me-24).

Reduction of **26** (**27** and **28**): To a solution of **26** (40 mg, 0.096 mmol) in dry toluene (1.9 mL) under argon atmosphere at −78 °C, (*S*)-2-methyl-CBS-oxazaborolidine (1.0 M in toluene; 0.19 mL, 0.19 mmol) and borane dimethylsulfide (1.0 M in toluene; 0.19 mL, 0.19 mmol) was added. The reaction mixture was stirred at −30 °C for 20 h. It was quenched with MeOH (2 mL) and it was allowed to warm to room temperature. Then it was added water and Et_2_O and extracted with Et_2_O. The combined organic layers were washed with water and brine, dried (Na_2_SO_4_), filtered, and concentrated *in vacuo*. The resulting crude residue was purified with a column with Amberlyst 15 (NH_4_^+^) and after that, with a flash CC (hexane-AcOEt, 85:15) to obtain **27** (19 mg, 52%) and **28** (16 mg, 42%). 

Methyl 16*R*-hydroxy-19,20-epoxy-luffara-8,13*Z*,17(20),18-tetraen-21-oate (**27**): ${\left[\text{α}\right]}_{\text{D}}^{20}$ = +33.3 (*c* 0.2, CHCl_3_); IR υ 3466 (OH), 3134, 2928, 1717 (C=O),1647 (C=C), 1437, 1375, 1219, 1026; ^1^H-NMR (400 MHz, CDCl_3_) δ 7.40 (1H, s, H-20), 7.39 (1H, s, H-19), 6.41 (1H, bs, H-18), 6.00 (1H, t, *J* = 7.9 Hz, H-14), 4.83–4.78 (1H, m, H-16), 3.77 (3H, s, *Me*COO), 2.87–2.83 (2H, m, H-15), 2.30 (2H, t, *J* = 8.7 Hz, H-12), 2.00–1.00 (13H, m), 1.57 (3H, s, Me-22), 0.93 (3H, s, Me-23), 0.88 (3H, s, Me-25), 0.83 (3H, s, Me-24); ^13^C-NMR (100 MHz, CDCl_3_) δ 168.9 (C), 143.2 (C), 139.7 (C), 138.9 (CH), 136.6 (CH), 135.6 (C), 128.9 (C), 126.6 (C), 108.5 (CH), 66.5 (CH), 51.8 (CH), 51.5 (CH_3_), 41.8 (CH_2_), 39.0 (C), 37.9 (CH_2_), 36.9 (CH_2_), 35.3 (CH_2_), 33.6 (CH_2_), 33.3 (C, CH_3_), 28.2 (CH_2_), 21.7 (CH_3_), 20.0 (CH_3_), 19.4 (CH_3_), 19.0 (CH_2_ - 2); HRMS (ESI) *m/z* calcd for C_26_H_38_O_4_Na (M + Na)^+^ 437.2662, found 437.2659.

19,20-Epoxy-luffara-8,13*Z*,17(20),18-tetraen-21,16*R*-olide (**28**): ${\left[\text{α}\right]}_{\text{D}}^{20}$ = +56.0 (*c* 0.26, CHCl_3_); IR υ 2924, 2855, 1724 (C=O), 1464, 1377, 1117; ^1^H-NMR (400 MHz, CDCl_3_) δ 7.49 (1H, s, H-20), 7.42 (1H, s, H-19), 6.46 (1H, bs, H-18), 6.65–6.60 (1H, m, H-14), 5.39 (1H, dd, *J* = 4.1 and 11.0 Hz, H-16), 3.15–3.00 (2H, m, H-15), 2.50–1.00 (15H, m), 1.62 (3H, s, Me-22), 0.95 (3H, s, Me-23), 0.89 (3H, s, Me-25), 0.84 (3H, s, Me-24); ^13^C-NMR (100 MHz, CDCl_3_) δ 165.0 (C), 143.6 (CH), 139.9 (CH), 139.5 (C), 137.2 (CH), 133.8 (C), 126.9 (C), 124.0 (C), 108.6 (CH), 72.3 (CH), 51.9 (CH), 41.8 (CH_2_), 39.0 (C), 37.0 (CH_2_), 33.6 (CH_2_), 33.3 (C, CH_3_), 31.6 (CH_2_), 30.5 (CH_2_), 27.7 (CH_2_), 21.7 (CH_3_), 20.1 (CH_3_), 19.5 (CH_3_), 19.0 (CH_2_-2); HRMS (ESI) *m/z* calcd for C_25_H_34_O_3_Na (M + Na)^+^ 405.2400, found 405.2405.

Reduction of **27** and **28** (**29**): To a solution of **27** (14 mg, 0.034 mmol) in DCM (0.3 mL) under argon atmosphere, DIBAL-H (1.0 M in hexane; 0.2 mL, 0.2 mmol) was added dropwise. The mixture was reacted at rt for 90 min and then, AcOEt was added. It was quenched with a saturated aqueous solution of potassium sodium tartrate (1 mL), and it was stirred for 15 min. After that it was extracted with AcOEt and the combined organic layers were washed with 6% aqueous solution of NaHCO_3_, water and brine, dried (Na_2_SO_4_), filtered, and concentrated *in vacuo*. The resulting crude residue was purified by CC (hexane-AcOEt 9:1) to obtain **29** (11 mg, 85%).

To a solution of **28** (3 mg, 0.008 mmol) in DCM (0.22 mL) under argon atmosphere, DIBAL-H (1.0 M in hexane; 48 μL, 0.05 mmol) was added dropwise. The mixture was reacted at rt for 2.5 h and then, AcOEt was added. It was quenched with a saturated aqueous solution of potassium sodium tartrate (1 mL), and it was stirred for 15 min. After that it was extracted with AcOEt and the combined organic layers were washed with 6% aqueous solution of NaHCO_3_, water and brine, dried (Na_2_SO_4_), filtered, and concentrated *in vacuo*. The resulting crude residue was purified by CC (hexane-AcOEt 9:1) to obtain **29** (3 mg, 95%).

19,20-Epoxy-luffara-8,13*Z*,17(20),18-tetraene-16*R*,21-diol (**29**): ${\left[\text{α}\right]}_{\text{D}}^{20}$ = +51.2 (*c* 0.37, CHCl_3_); IR υ 3345 (OH), 2928, 2866, 1456, 1161, 1024; ^1^H-NMR (400 MHz, CDCl_3_) δ 7.40 (2H, s, H-19, H-20), 6.41 (1H, s, H-18), 5.41 (1H, t, *J* = 7.9 Hz, H-14), 4.73 (1H, dd, *J* = 4.4 and 8.0 Hz, H-16), 4.20 (1H, d, *J* = 11.6 Hz, H_A_-21), 4.05 (1H, d, *J* = 11.6 Hz, H_B_-21), 2.60–2.50 (2H, m, H-15), 2.20–2.10 (4H, m, H-11, H-12), 2.00–1.00 (11H, m), 1.58 (3H, s, Me-22), 0.95 (3H, s, Me-23), 0.88 (3H, s, Me-25), 0.83 (3H, s, Me-24); ^13^C-NMR (100 MHz, CDCl_3_) δ 144.0 (C), 143.3 (CH), 140.1 (C), 138.9 (CH), 128.7(C), 126.1 (C), 123.0 (CH), 108.5 (CH), 66.0 (CH), 60.2 (CH_2_), 51.9 (CH), 41.8 (CH_2_), 39.0 (C), 37.1 (CH_2_), 37.0 (CH_2_), 36.3 (CH_2_), 33.6 (CH_2_), 33.3 (C, CH_3_), 27.4 (CH_2_), 21.7 (CH_3_), 20.1 (CH_3_), 19.5 (CH_3_), 19.0 (CH_2_-2); HRMS (ESI) *m/z* calcd for C_25_H_38_O_3_Na (M + Na)^+^ 409.2713, found 409.2695.

16*R*,20(*R*,*S*),21-Trihydroxy-luffara-8,13*Z*,17-trien-19,20-olide (**30**): To a solution of **29** (15 mg, 0.038 mmol) in DCM (5.6 mL), DIPEA (74 μL, 0.38 mmol) and Bengal Rose (1 mg) were added. After that, anhydrous oxygen was bubbled in for 10 min, the mixture was cooled to −78 °C and under oxygen atmosphere, it was irradiated by 200 W light for 5 h. Then, it was allowed to warm to room temperature and oxalic acid (5 mL) was added. The mixture was stirred for 30 min. Afterwards water was added and the mixture was extracted with DCM. The combined organic layers were washed with water, dried (Na_2_SO_4_), filtered, and concentrated *in vacuo*. The resulting crude residue was purified by flash CC (hexane-AcOEt; 1:1) to afford **30** (16 mg, 99%); IR υ 3308 (OH), 2924, 2851, 1748 (C=O), 1456, 1261, 1024; ^1^H-NMR (400 MHz, CDCl_3_) δ 6.17 (1H, s, H-20), 6.03 (1H, s, H-18), 5.45–5.35 (1H, m, H-14), 4.80–4.70 (1H, m, H-16), 4.25–4.00 (2H, m, H-21), 2.65-2.55 (2H, m, H-15), 2.20–2.10 (4H, m, H-11, H-12), 2.00–1.00 (11H, m), 1.56 (3H, s, Me-22), 0.94 (3H, s, Me-23), 0.88 (3H, s, Me-25), 0.83 (3H, s, Me-24); ^13^C-NMR (100 MHz, CDCl_3_) δ 167.8 (C), 167.7 (C), 144.0(C), 139.8 (C), 126.2 (C), 122.5 (CH), 117.8 (CH), 98.0 (CH), 68.1 (CH), 60.0 (CH_2_), 51.9 (CH), 42.0 (CH_2_), 39.0 (C), 37.4 (CH_2_), 36.7 (CH_2_), 33.7 (CH_2_), 33.6 (CH_2_), 33.3 (C, CH_3_), 27.2 (CH_2_), 21.7 (CH_3_), 20.1 (CH_3_), 19.5 (CH_3_), 19.0 (CH_2_-2).

16*R*,21-Dihydroxy-luffara-8,13*Z*,17-trien-19,20-olide (Luffarin I (**9**)): To a solution of **30** (7 mg, 0.017 mmol) in absolute ethanol (1.1 mL) at 0 °C, NaBH4 (2 mg, 0.061 mmol) was added. After 5 min, water and 2 M aqueous solution of HCl was added. It was extracted with AcOEt and the combined organic layers were washed with water until neutral pH was reached and brine, dried (Na2SO4), filtered, and concentrated *in vacuo*. The resulting crude residue was purified by CC (hexane-AcOEt; 1:1) to obtain **9** (5 mg, 71%). ${\left[\text{α}\right]}_{\text{D}}^{20}$ = +69.0 (*c* 0.51, CHCl_3_); IR υ 3387 (OH), 2926, 2866, 1780 (C=O), 1748, 1638 (C=C), 1456, 1026; ^1^H-NMR (400 MHz, CDCl_3_) δ 5.99 (1H, bs, H-18), 5.40 (1H, t, *J* = 8.1 Hz, H-14), 4.89 (2H, bs, H-20), 4.66 (1H, t, *J* = 5.9 Hz, H-16), 4.22 (1H, d, *J* = 11.6 Hz, H_A_-21), 4.14 (1H, d, *J* = 11.6 Hz, H_B_-21), 2.60–2.50 (2H, m, H-15 ), 2.20–2.00 (4H, m, H-11, H-12), 2.00–1.00 (11H, m), 1.57 (3H, s, Me-22), 0.94 (3H, s, Me-23), 0.88 (3H, s, Me-25), 0.83 (3H, s, Me-24); ^13^C-NMR (100 MHz, CDCl_3_) δ 173.6 (C), 172.3 (C), 145.1 (C), 139.7 (C), 126.4 (C), 121.8 (CH), 114.9 (CH), 71.3 (CH_2_), 67.4 (CH), 60.3 (CH_2_), 51.8 (CH), 41.7 (CH_2_), 39.0 (C), 37.6 (CH_2_), 37.0 (CH_2_), 35.1 (CH_2_), 33.6 (CH_2_), 33.3 (C, CH_3_), 27.3 (CH_2_), 21.6 (CH_3_), 20.1 (CH_3_), 19.5 (CH_3_), 19.0 (CH_2_ - 2); HRMS (ESI) *m/z* calcd for C_25_H_38_O_4_Na (M + Na)^+^ 425.2662, found 425.2671.

Supplementary files available. Copies of IR, HRMS, NMR spectra and study of C-16 stereochemistry of compound 29 using Mosher’s methodology are included.

## 4. Conclusions

The first synthesis of Luffarin I has been achieved from (−)-sclareol confirming its structure and absolute configuration as 5*S*, 10*S*, 16*R*. This methodology opens the way for the synthesis of other marine natural compounds of this class. The study of the antiproliferative activity of Luffarin I showed remarkable biological activity towards human cancer cell lines. A more detailed structure-activity relationship study may be necessary in order to establish the scope and limitations of the new scaffold. Experiments needed to validate the usefulness of this compound as potential anticancer drug are in progress and will be reported elsewhere.

## Supplementary Files

Supplementary File 1
