# Supplementary material for: Synthesis and Bioactivity of Luffarin I
Source: Mar Drugs. 2015 Apr 20;13(4):2407–23. doi: 10.3390/md13042407 (PMC4413218; doi:10.3390/md13042407)
Supplement: Supplementary File 1 [file marinedrugs-13-02407-s001.pdf]

Supplementary Information

As it has shown in the manuscript treatment of **26** with (*S*)-2-methyl-CBS-oxazaborolidine using borane dimethysulfide as reducting agent under inert atmosphere and low temperature, produced diastereoselectively **27** and **28**.

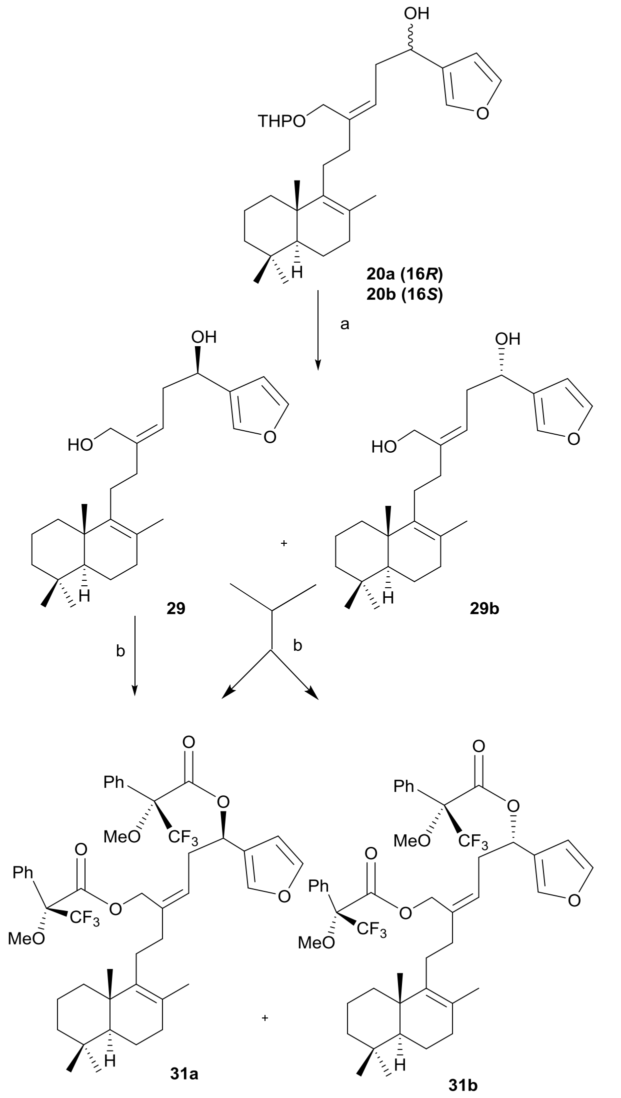

Scheme S1. Synthesis of **31a/31b**. *Reagents and conditions:* (**a**) *p*-TsOH, MeOH, RT, 66%; (**b**) (+)−MTPA, DMAP, DCC, DCM, 0 ℃-RT, 24 h, 45%.

The generated stereogenic center at C-16 has *R* configuration as expected. Confirmation of the diastereoselectivity of the reduction was done applying Mosher methodology (Scheme S1). The mixture of diastereoisomers **29/29b**, obtained after deprotection of mixture **20a/20b**, was esterified with (+)−MTPA leading to the diester mixture **31a/31b** (Scheme S1).

Comparison between the ^{1}H NMR spectra of the mixture (**29/29b**) and compound **29** obtained from Corey-Bakshi-Shibata reduction indicated that this reaction afforded only one of the diastereoisomers.

Isobe *et al.* [1] have established that for secondary alcohols with an α-furyl substituent, the ring protons (H-18 in this case) can be used for the analysis that allow determination of the absolute configuration of the centre observed.

Tables S1 and S2 show the chemical shifts for H-18 for **29** and **31a** as well as for **29b** and **31b**.

Tables S1. Comparative studies for the signals of H-18 in ¹H-NMR for compounds **29** and **31a**.

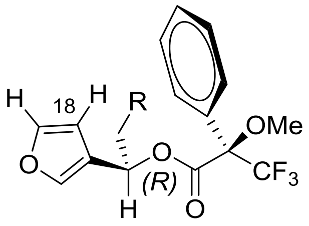

	**29**	**31a**
δ **H-18** (ppm)	6.41	6.40

Table S2. Comparative studies for the signals of H-18 in ¹H-NMR for compounds **29b** and **31b**.

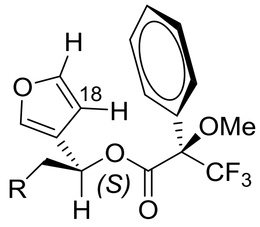

	**29b**	**31b**
δ **H-18** (ppm)	6.41	6.28

The comparison between the chemical shifts of H-18 in **31a** (δ 6.40) and **31b** (δ 6.26) permit us to conclude that the isomer with the signal in the ¹H NMR spectrum of H-18, at lower field (**31a**) is 16*R* and the one at higher field (**31b**) is 16*S*, due to the shielding effect of the aromatic ring. This analysis confirmed the diastereoselectivity of the reduction of **26** that lead to compounds **27** and **28** [1].

19,20-Epoxy-luffara-8,13*Z*,17(20),18-tetraen-16(*R*,*S*),21-diol **(29/29b)**: To a solution of **20a/20b** (14 mg, 0.03 mmol) in MeOH (3.5 mL) was added *p*-toluenesufonic acid (1 mg, 0.005 mmol). The reaction mixture was stirred for 48 h. Then, water was added and it was extracted with AcOEt. The combined organic layers were washed with water and brine, dried (Na_2SO_4), filtered, and concentrated in vacuo to obtain **29/29b** (7 mg, 66%). IR υ 3345 (OH), 2928, 2866, 1456, 1161, 1024; ¹H-NMR (400 MHz, $CDCl_3$) δ 7.40 (2H, s, H-19, H-20), 6.41 (1H, s, H-18), 5.41 (1H, t, *J* = 7.9 Hz, H-14), 4.73 (1H, dd, *J* = 4.4 and 8.0 Hz, H-16), 4.20 (1H, d, *J* = 11.6 Hz, H_A-21), 4.05 (1H, d, *J* = 11.6 Hz, H_B-21), 2.60–2.50 (2H, m, H-15), 2.20–2.10 (4H, m, H-11, H-12), 2.00–1.00 (11H, m), 1.58 (3H, s, Me-22), 0.95 (3H, s, Me-23), 0.88 (3H, s, Me-25), 0.83 (3H, s, Me-24); ¹³C-NMR (100 MHz, $CDCl_3$) δ 144.0 (C), 143.3 (CH), 140.1 (C), 138.9 (CH), 128.7 (C), 126.1 (C), 123.0 (CH), 108.5 (CH), 66.0 (CH), 60.2 (CH_2), 51.9 (CH), 41.8 (CH_2), 39.0 (C), 37.1 (CH_2), 37.0 (CH_2), 36.3 (CH_2), 33.6 (CH_2), 33.3 (C, CH_3), 27.4 (CH_2), 21.7 (CH_3), 20.1 (CH_3), 19.5 (CH_3), 19.0 (CH_2 × 2); HRMS (ESI) *m/z* calcd for $C_{25}H_{38}O_3Na$ $(M + Na)^+$ 409.2713, found 409.2695.

Di-(*R*-α-methoxy-α-trifluoromethyl-phenyl)-acetate of 19,20-epoxy-luffara-8,13*Z*,17(20), 18-tetraen-16(*R*,*S*),21-diol (**31a/31b**): To a solution of **29/29b** (7 mg, 0.017 mmol) in DCM (0.6 mL), *R*-α-methoxy-α-trifluoromethylphenylacetic acid (9 mg, 0.04 mmol) and DMAP (1 mg, 0.006 mmol)

were added. It was cooled to 0 ℃ and under argon atmosphere, *N*,*N*′-dicyclohexylcarbodiimide (DCC) (38 μL, 0.38mmol) was added. The mixture was reacted at 0 ℃ for 90 min, and then, it was allowed to warm to room temperature. The reaction mixture was stirred for 24 h. The resulting crude was filtered and water was added. Then it was extracted with AcOEt and the combined organic layers were washed with 2 M aqueous solution of HCl, 10% aqueous solution of $NaHCO_3$ and water until neutral pH was reached, dried (Na_2SO_4), filtered, and concentrated in vacuo to obtain **31a/31b** (6 mg, 45%). $[\alpha]_D^{20}$= +28.7 (*c* 0.39, $CHCl_3$); IR υ 2932, 2859, 1748 (C=O), 1668 (C=C), 1456, 1271, 1169, 1022; ^{1}H-NMR (400 MHz, $CDCl_3$) δ 7.45–7.30 (12H, m, H-19, H-20, Ph-), 6.39 (1H, s, H-18, 16*R*, minor.), 6,27 (1H, s, H-18, 16*S*, major.), 5.98 (1H, t, *J* = 5.8 Hz, H-16 major.), 5.97 (1H, t, *J* = 5.8 Hz, H-16 minor.), 5.46 (1H, t, *J* = 7.2 Hz, H-14, major.), 5.37 (1H, t, *J* = 7.8 Hz, H-14, minor.), 4.83 (1H, d, *J* = 12.0 Hz, H_A-21, major.), 4.78 (1H, d, *J* = 12.0 Hz, HA-21, minor.), 4.70 (1H, d, *J* = 12.0 Hz, H_B-21, major.), 4.63 (1H, d, *J* = 12.0 Hz, H_B-21, minor.), 3.51 (3H, s, *Me*O), 3.50 (6H, s, *Me*O), 3.45 (3H, s, *Me*O), 2.85–2.60 (2H, m, H-15), 2.20–2.00 (4H, m, H-11, H-12), 2.00–1.00 (11H, m), 1.54 (3H, s, Me-22), 0.94 (3H, s, Me-23), 0.88 (3H, s, Me-25), 0.82 (3H, s, Me-24); HRMS (ESI) *m/z* calcd for $C_{45}H_{56}NO_7F_6$ (M + NH_4) 836.3956, found 836.3985.

Di-(*R*-α-methoxy-α-trifluoromethyl-phenyl)-acetate of 19,20-epoxy-luffara-8,13*Z*,17(20), 18-tetraen-16*R*,21-diol (**31a**): To a solution of **29** (5 mg, 0.013 mmol) in DCM (0.45 mL), R-α-methoxy-α-trifluoromethylphenylacetic acid (7 mg, 0.03 mmol) and DMAP (1 mg, 0.005 mmol) were added. It was cooled to 0 ℃ and under argon atmosphere, DCC (28 μL, 0.28mmol) was added. The mixture was reacted at 0 ℃ for 90 min, and then, it was allowed to warm to room temperature. The reaction mixture was stirred for 24 h. The resulting crude was filtered and water was added. Then it was extracted with AcOEt and the combined organic layers were washed with 2 M aqueous solution of HCl, 10% aqueous solution of $NaHCO_3$ and water until neutral pH was reached, dried (Na_2SO_4), filtered, and concentrated in vacuo to obtain **31a** (9 mg, 80%). $[\alpha]_D^{20}$= +12.3 (*c* 0.36, CHCl3); IR υ 3065, 2926, 2855, 1748 (C=O), 1670 (C=C), 1464, 1271, 1169, 1022; ^{1}H-NMR (400 MHz, $CDCl_3$) δ 7.45-7.30 (12H, m, H-19, H-20, Ph-), 6.39 (1H, s, H-18), 5.97 (1H, t, *J* = 5.8 Hz, H-16), 5.37 (1H, t, *J* = 7.8 Hz, H-14), 4.78 (1H, d, *J* = 12.0 Hz, H_A-21), 4.63 (1H, d, *J* = 12.0 Hz, H_B-21), 3.50 (3H, s, *Me*O), 3.45 (3H, s, *Me*O), 2.85–2.60 (2H, m, H-15), 2.20–2.00 (4H, m, H-11, H-12), 2.00–1.00 (11H, m), 1.54 (3H, s, Me-22), 0.94 (3H, s, Me-23), 0.88 (3H, s, Me-25), 0.82 (3H, s, Me-24); HRMS (ESI) *m/z* calcd for $C_{45}H_{56}NO_7F_6$ (M+NH_4) 836.3956, found 836.3985.

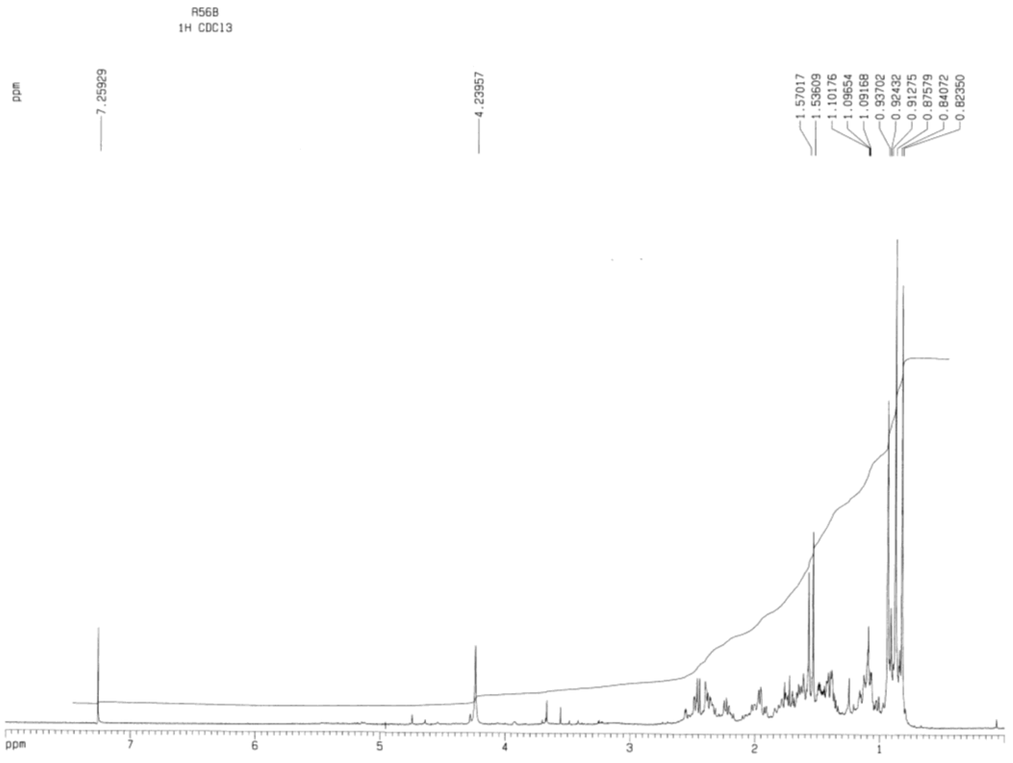

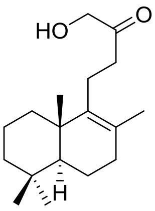

16

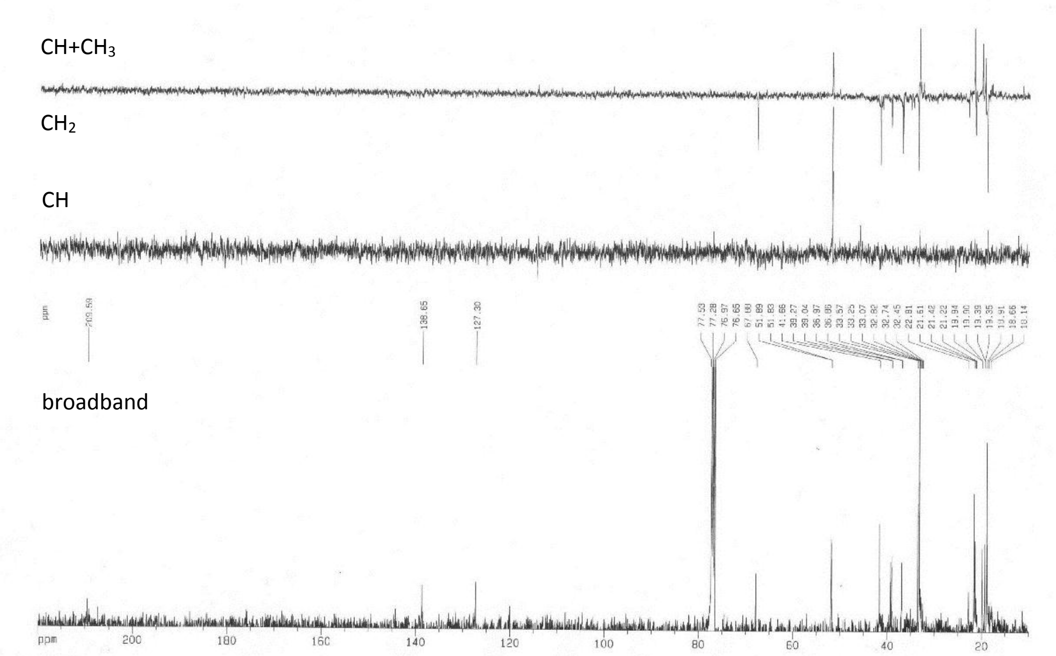

Figure S1. ^{1}H-NMR $CDCl_3$ and ^{13}C-NMR $CDCl_3$.

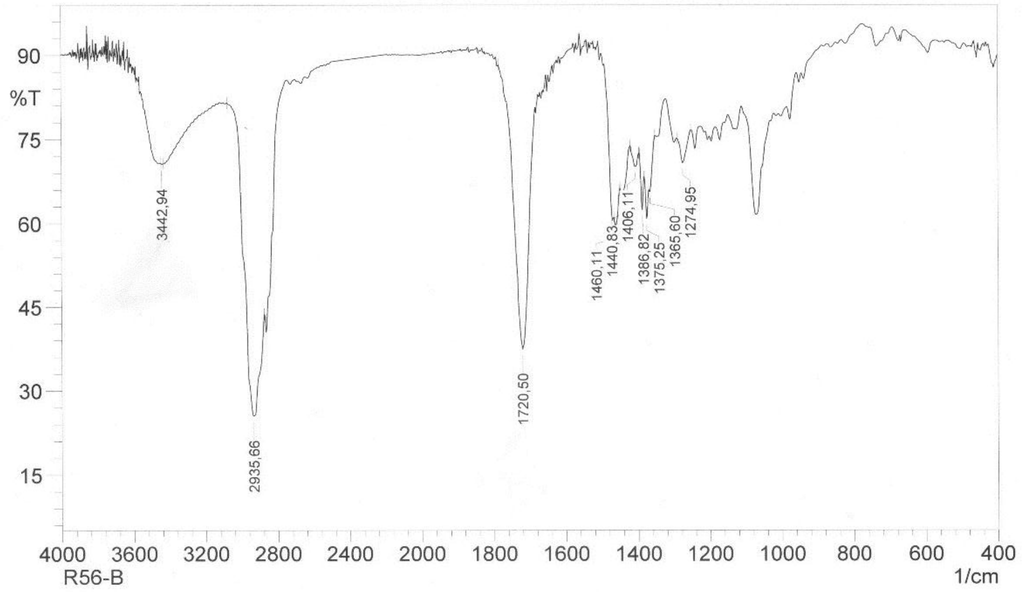

Figure S2. IR of **16**.

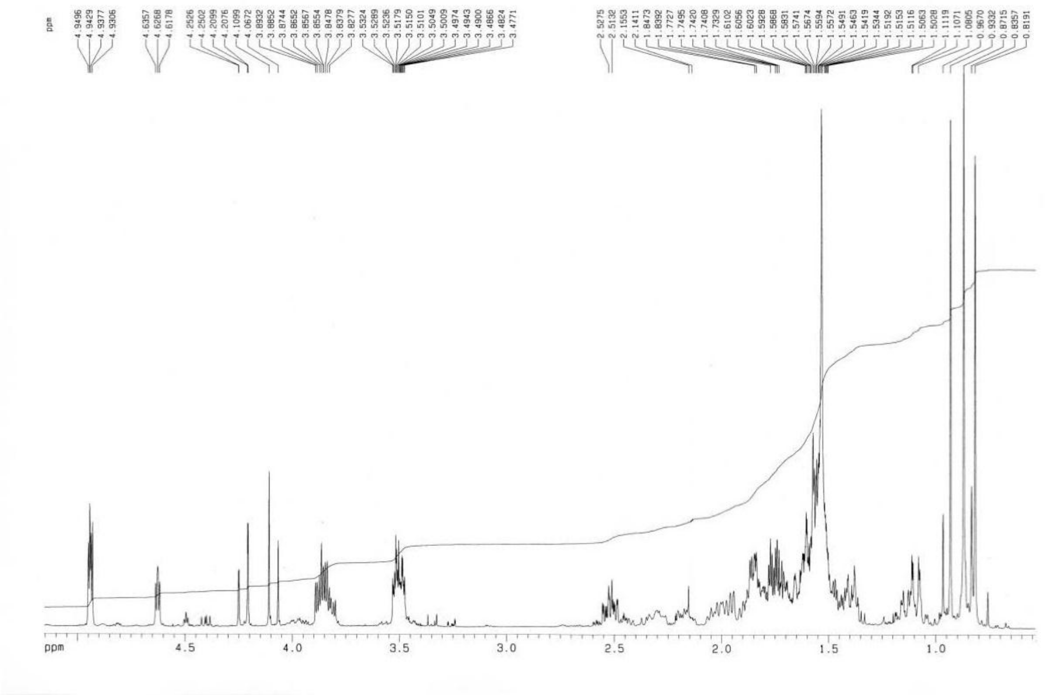

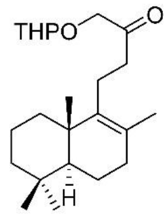

17

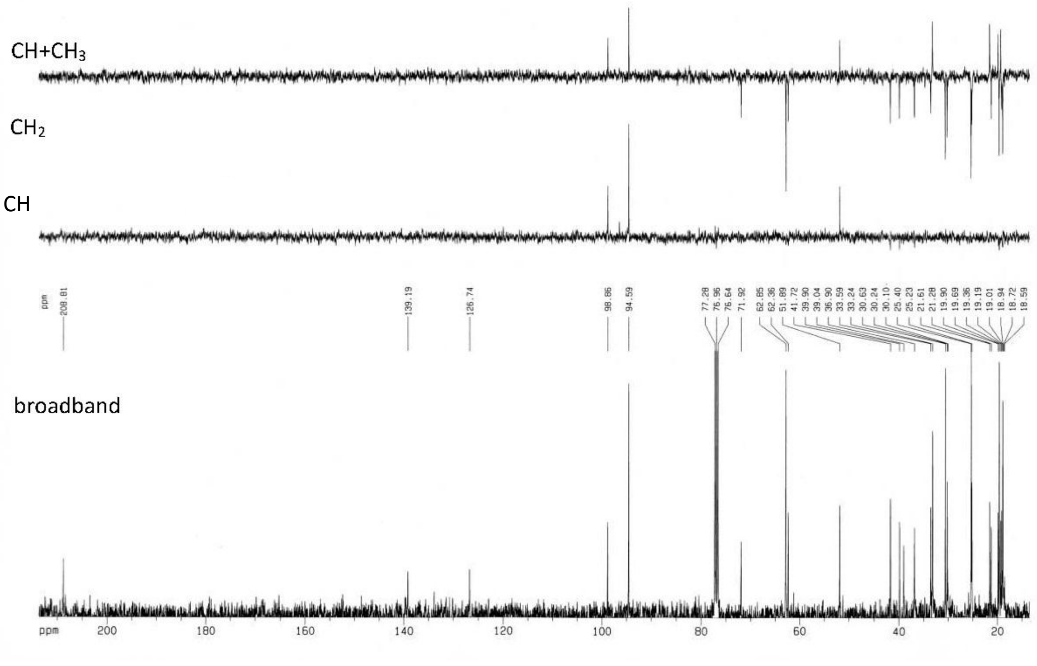

Figure S3. ^{1}H-NMR $CDCl_3$ and ^{13}C-NMR $CDCl_3$.

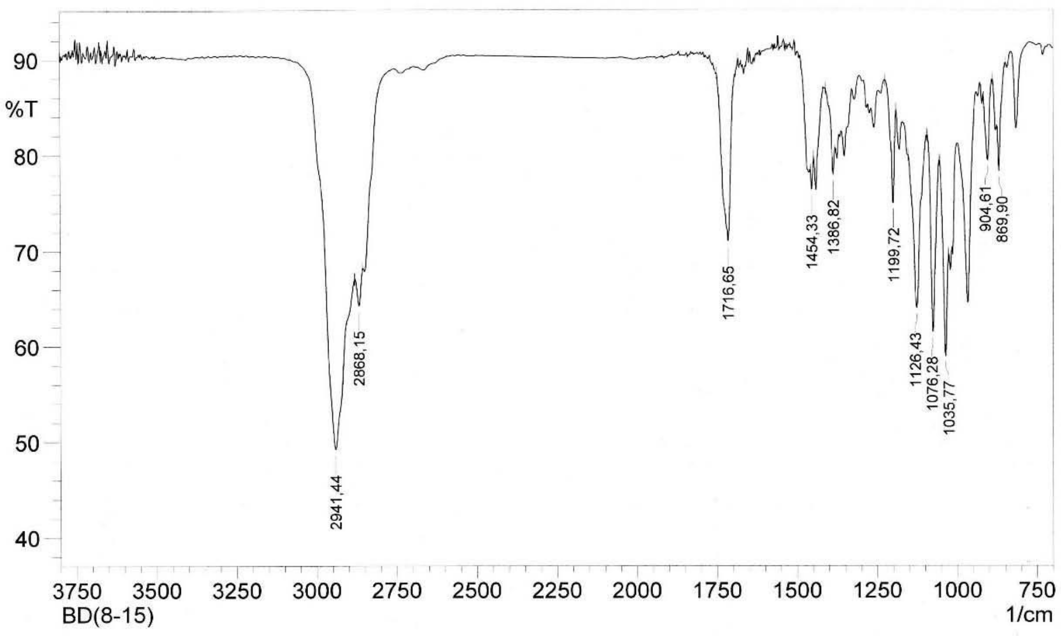

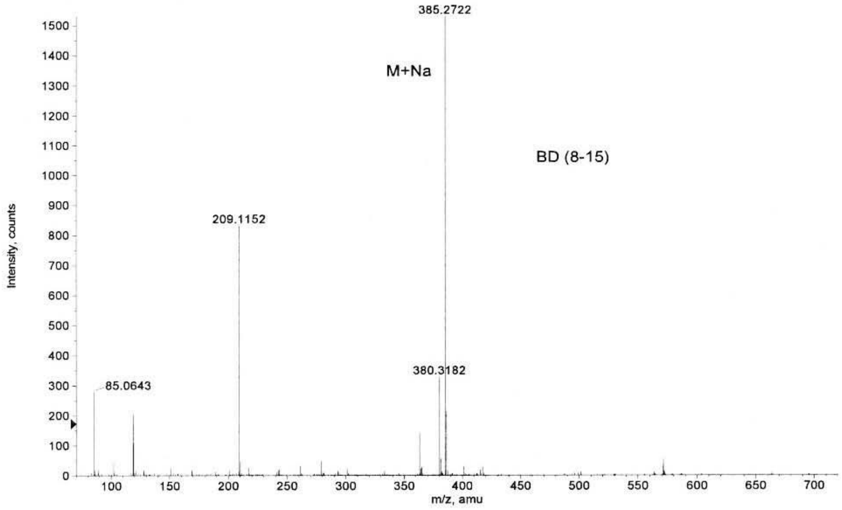

Formula	CalculatedMass	mDaError	ppmError	RDB
C23 H38 O3 Na	385.271317	0.88344	2.293025	4.5

Figure S4. IR and HRMS of **17**.

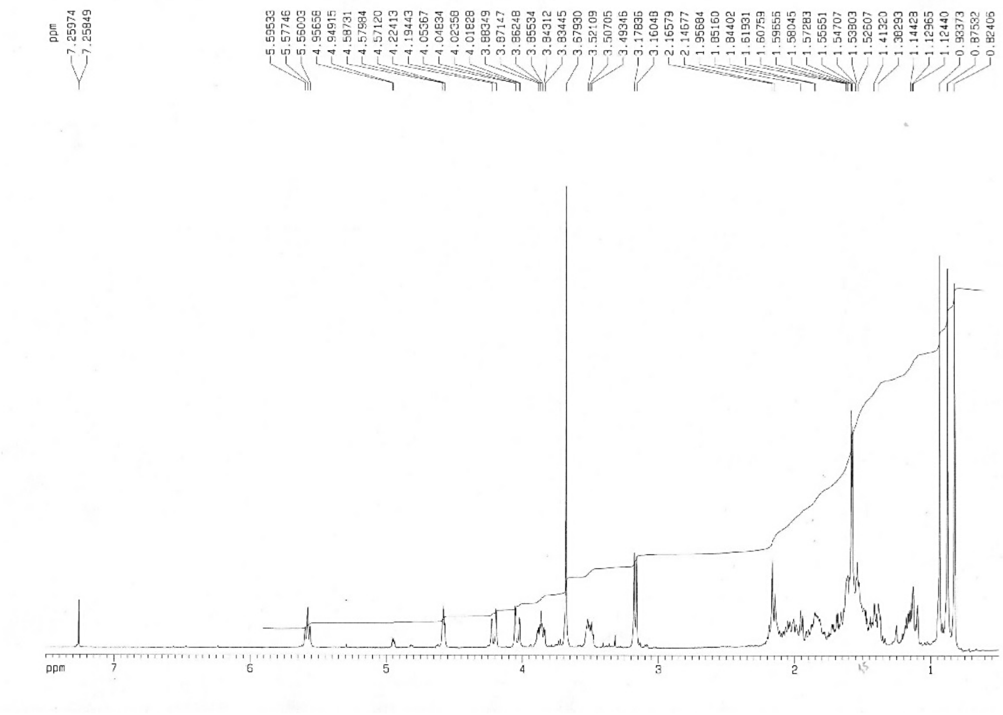

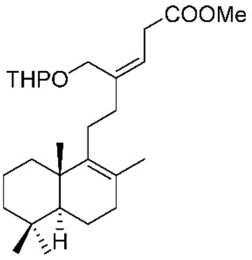

18

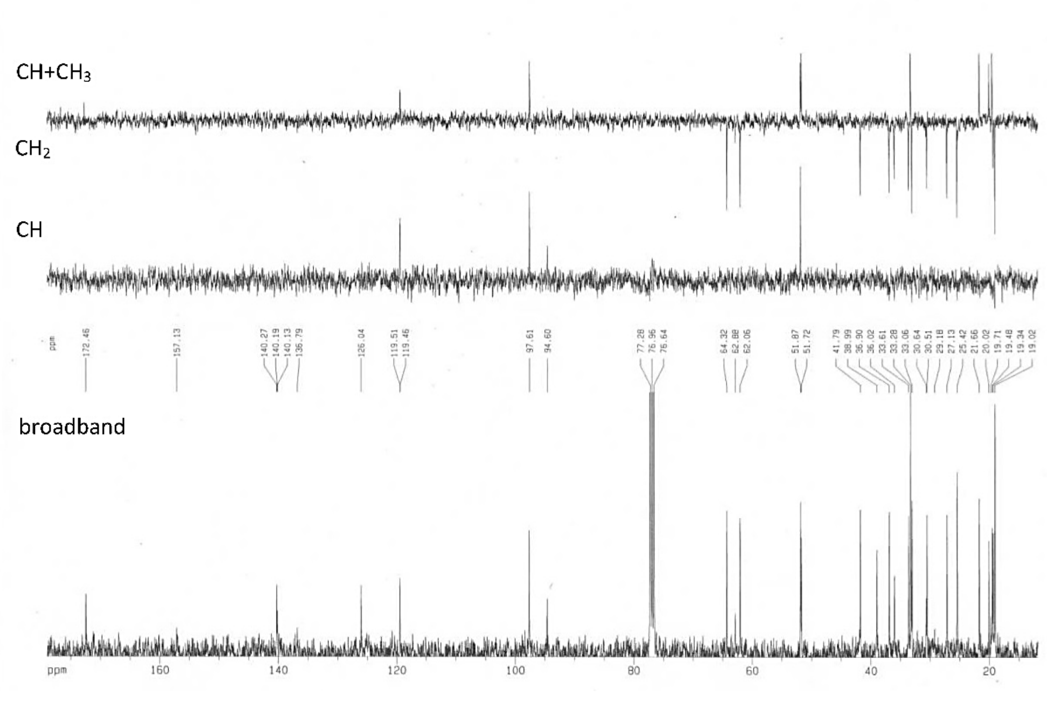

Figure S5. ^{1}H-NMR CDCl3 and ^{13}C-NMR CDCl3.

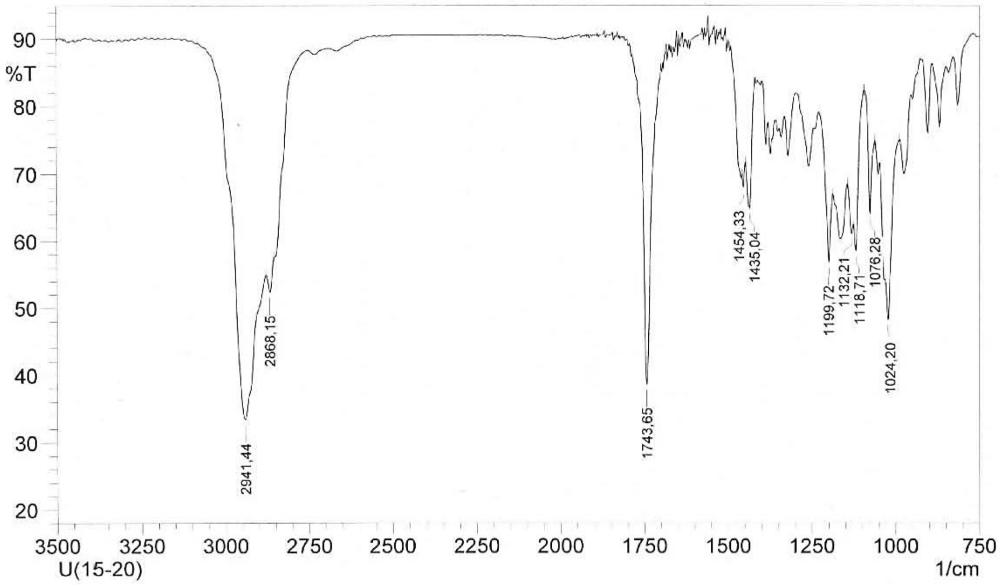

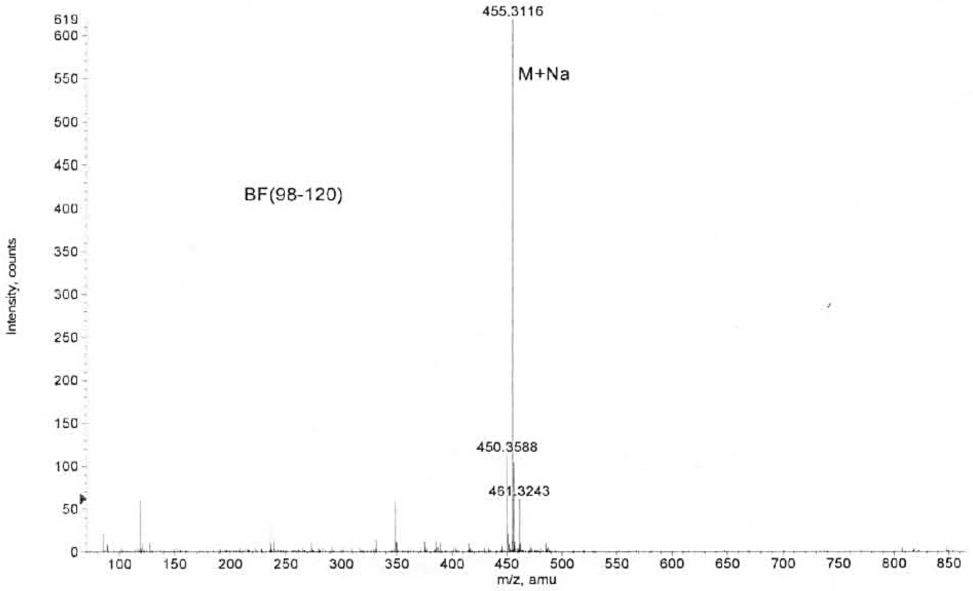

Formula	CalculatedMass	mDaError	ppmError	RDB
C24 H43 N2 O6	455.311564	0.036004	0.079075	4.5
C23 H40 N6 O2 Na	455.310496	1.103952	2.424605	6.5
C25 H39 N6 O2	455.312901	-1.301308	-2.858057	9.5
C21 H35 N12	455.310216	1.384084	3.039857	10.5
C27 H44 O4 Na	455.313181	-1.58144	-3.473309	5.5

Figure S6. IR and HRMS of **18**.

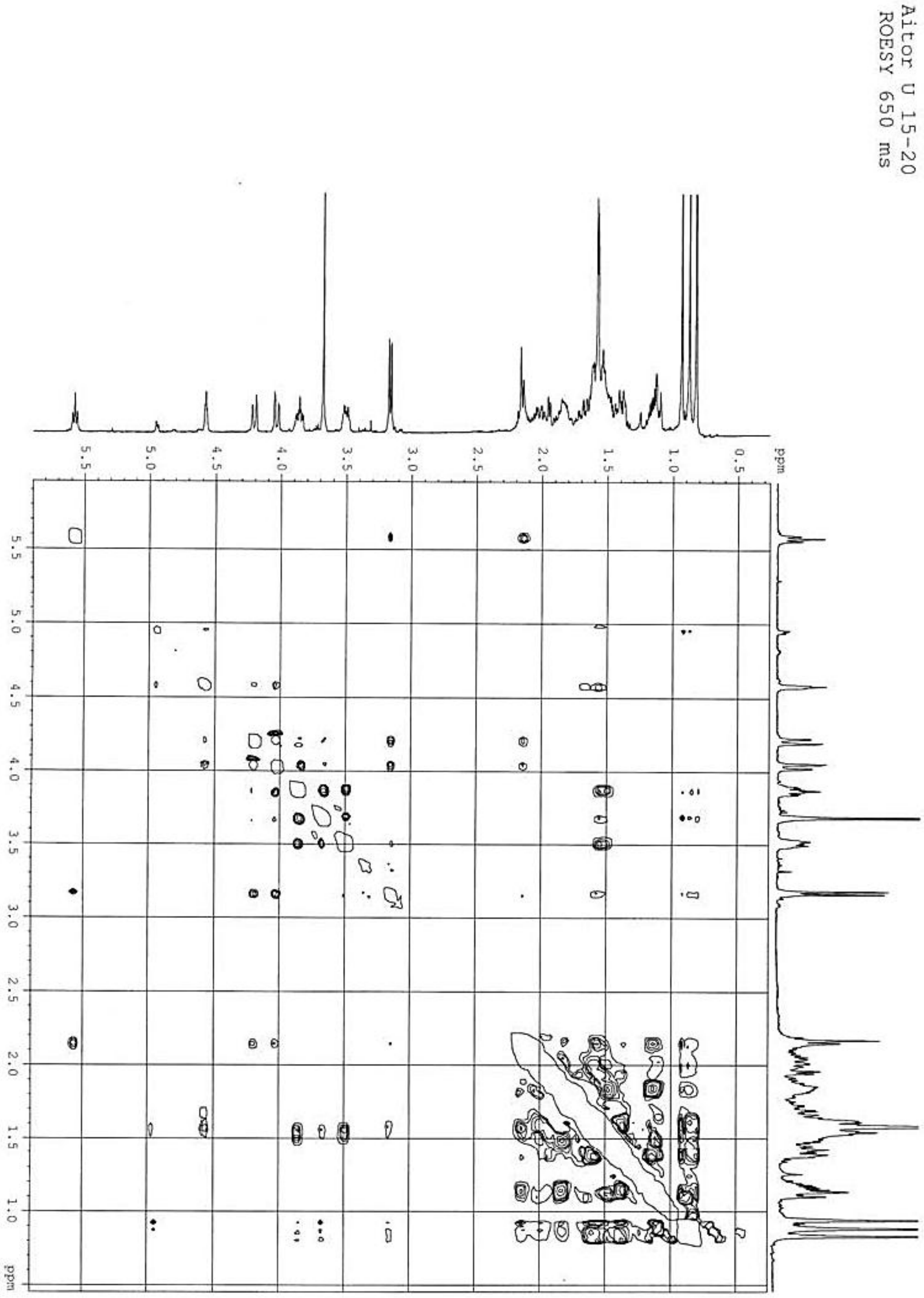

Figure S7. ROESY of **18**.

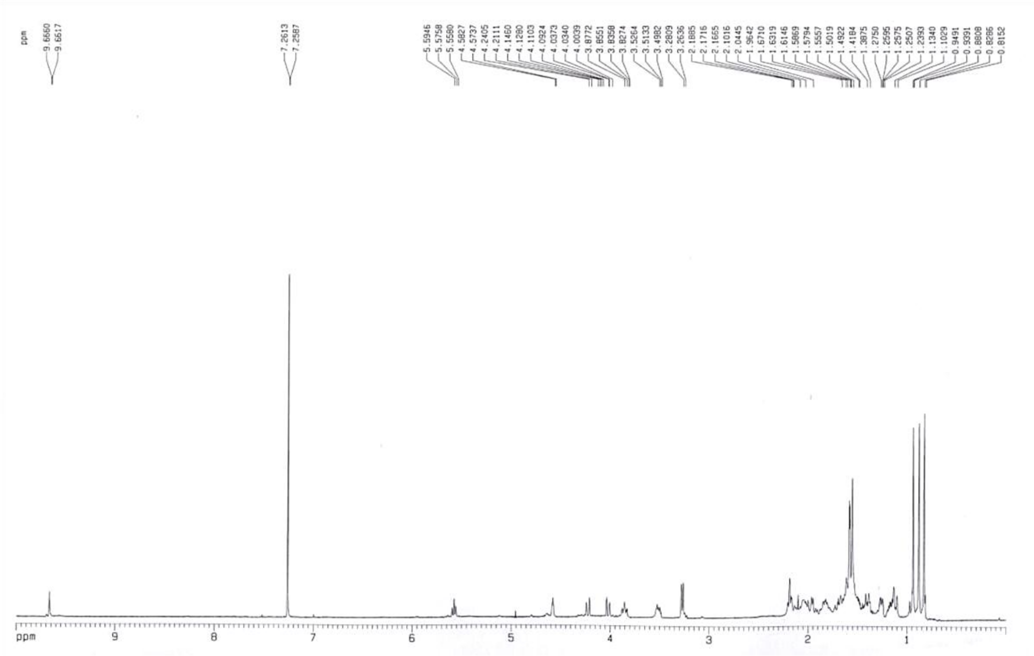

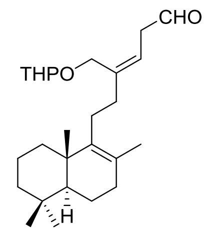

19

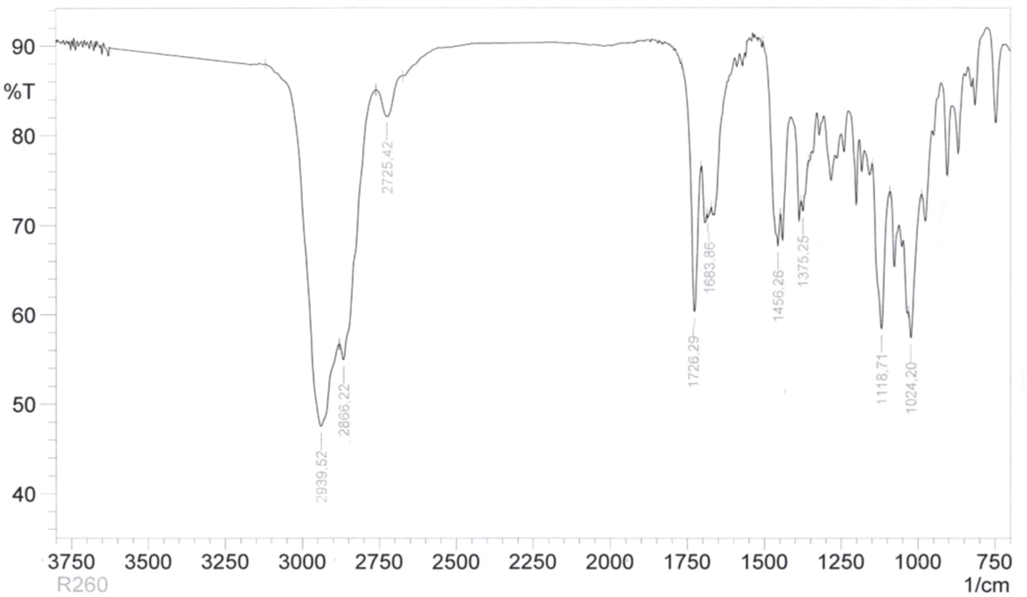

Figure S8. ^{1}H-NMR $CDCl_3$ and IR.

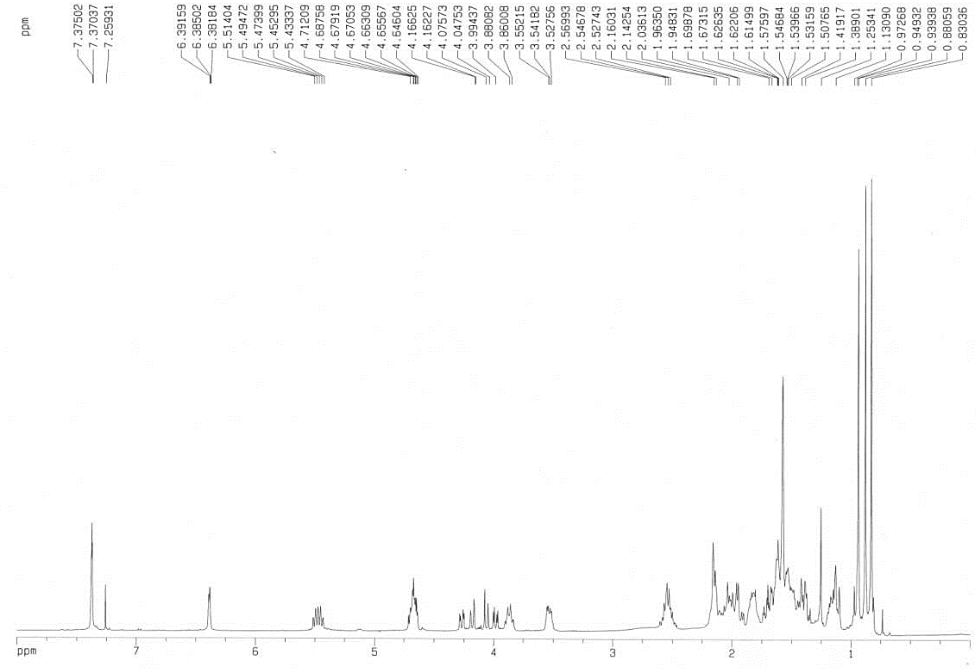

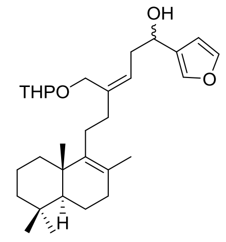

20a/20b

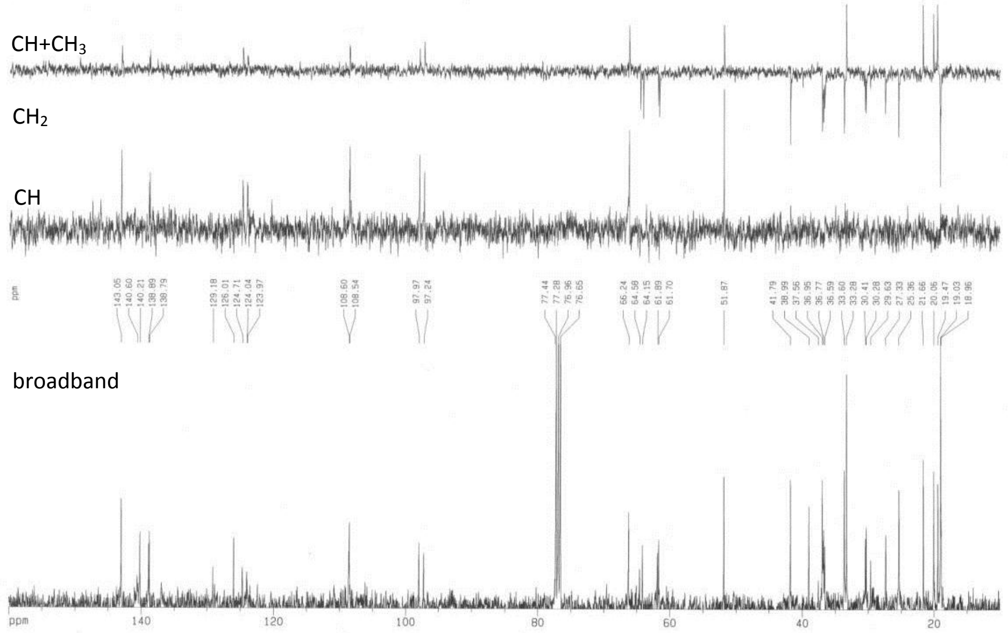

Figure S9. ^{1}H-NMR $CDCl_3$ and ^{13}C-NMR $CDCl_3$.

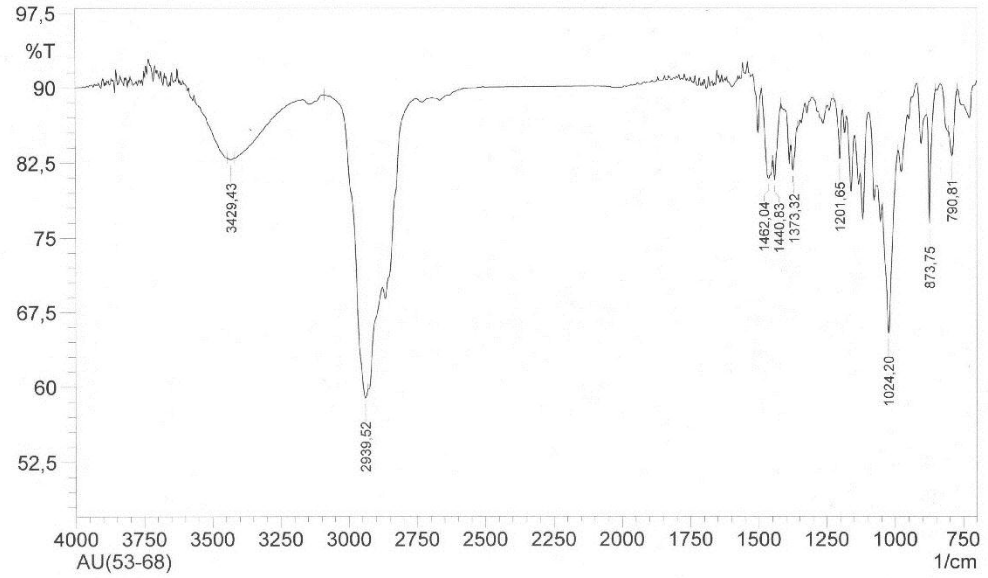

Figure S10. IR and HRMS of **20a/20b**.

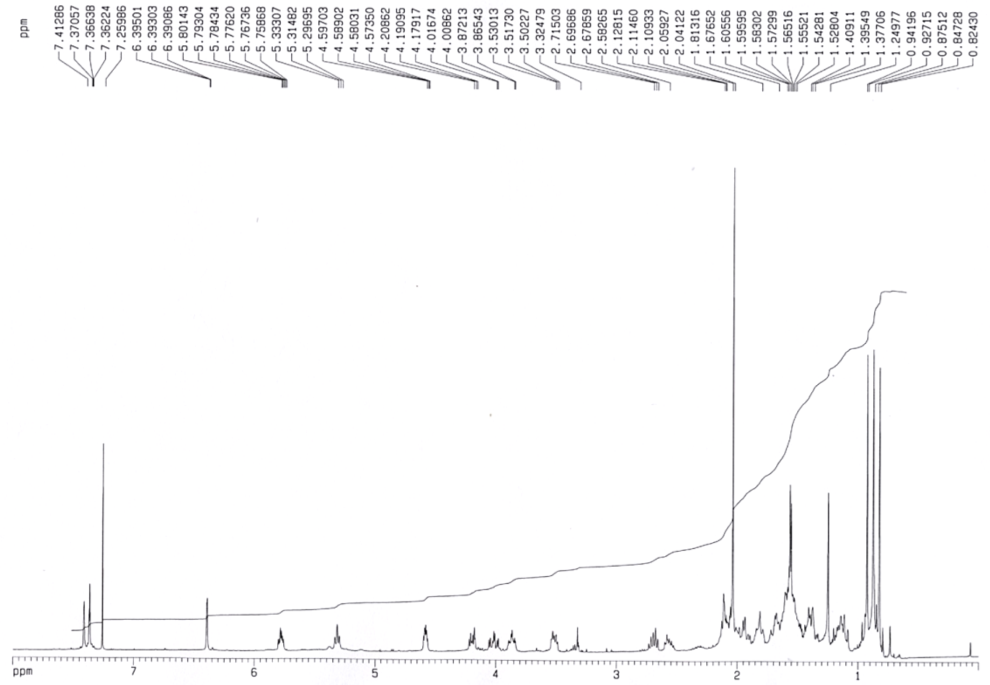

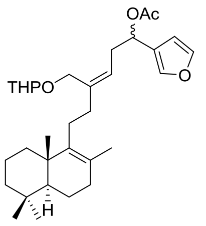

21a/21b

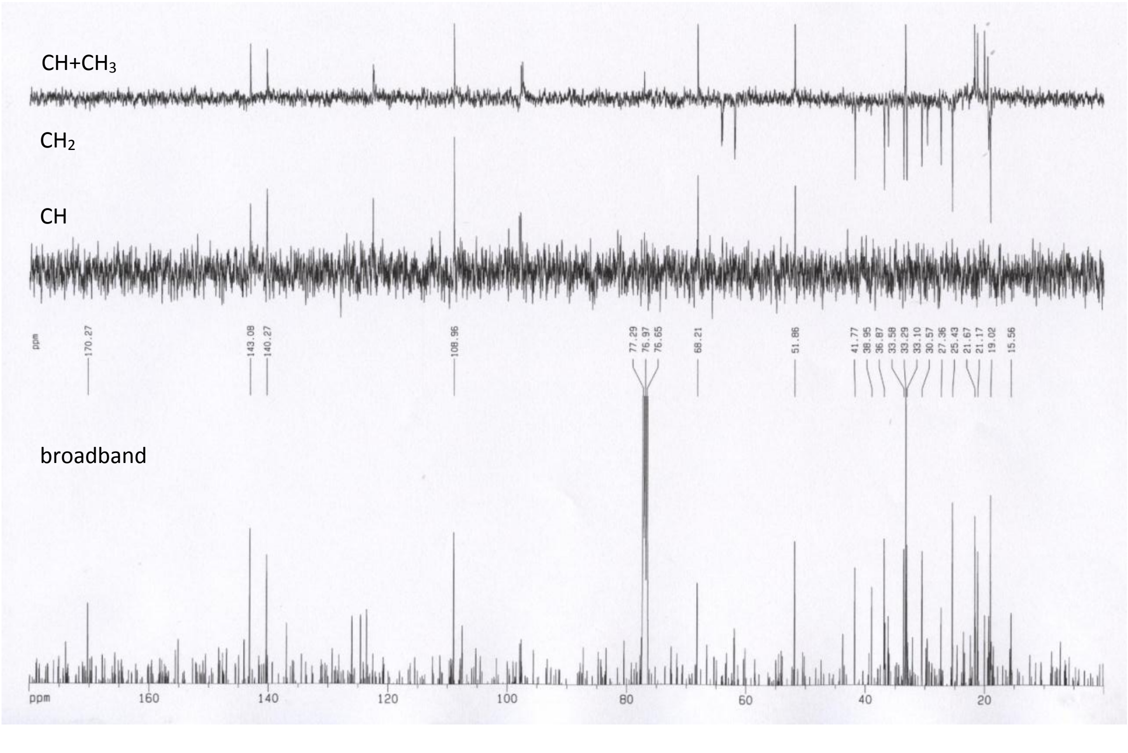

Figure S11. ^{1}H-NMR $CDCl_3$ and ^{13}C-NMR $CDCl_3$.

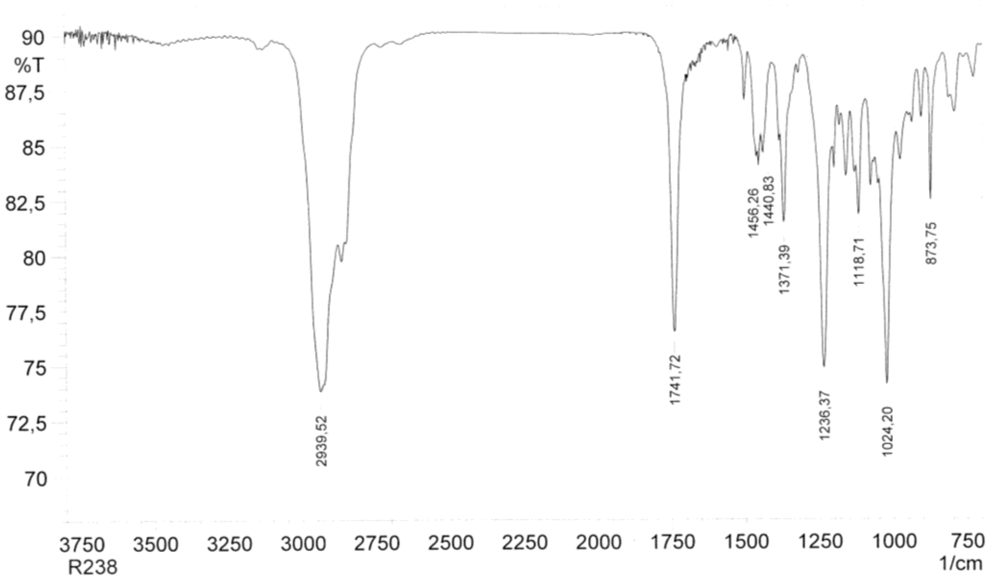

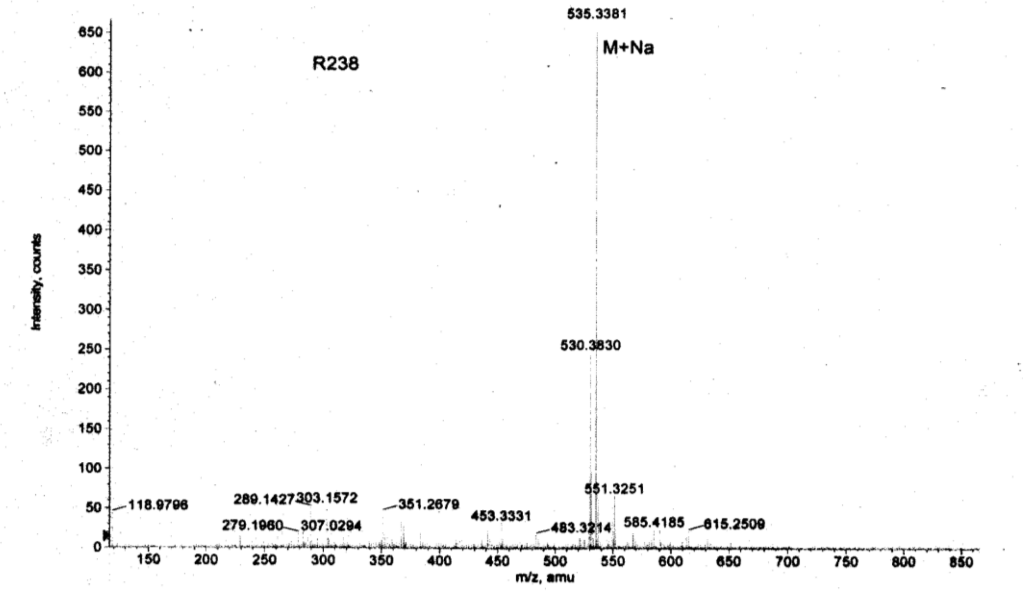

Formula	CalculatedMass	mDaError	ppmError	RDB
C32 H48 O5 Na	535.339396	-1.29624	-2.421346	8.5
C41 H43	535.335928	2.17186	4.056983	20.5
C34 H47 O5	535.341801	-3.7015	-6.914315	11.5
C39 H44 Na	535.333523	4.57712	8.549952	17.5

Figure S12. IR and HRMS of **21a/21b**.

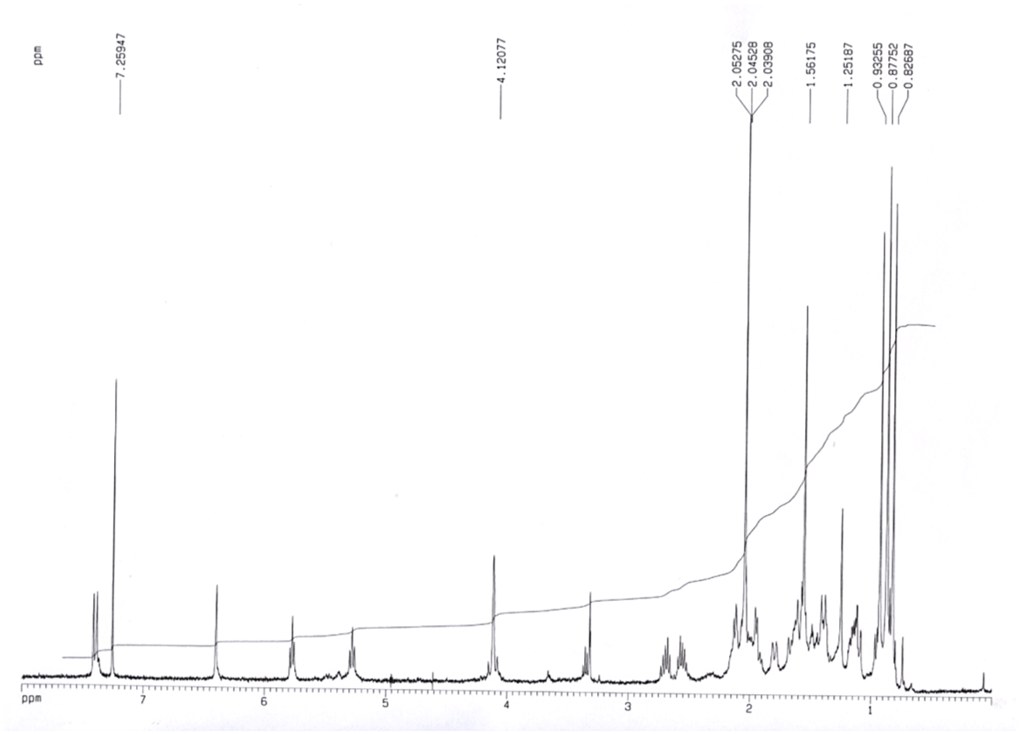

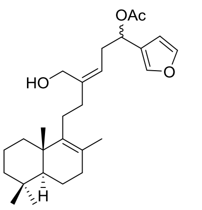

22a/22b

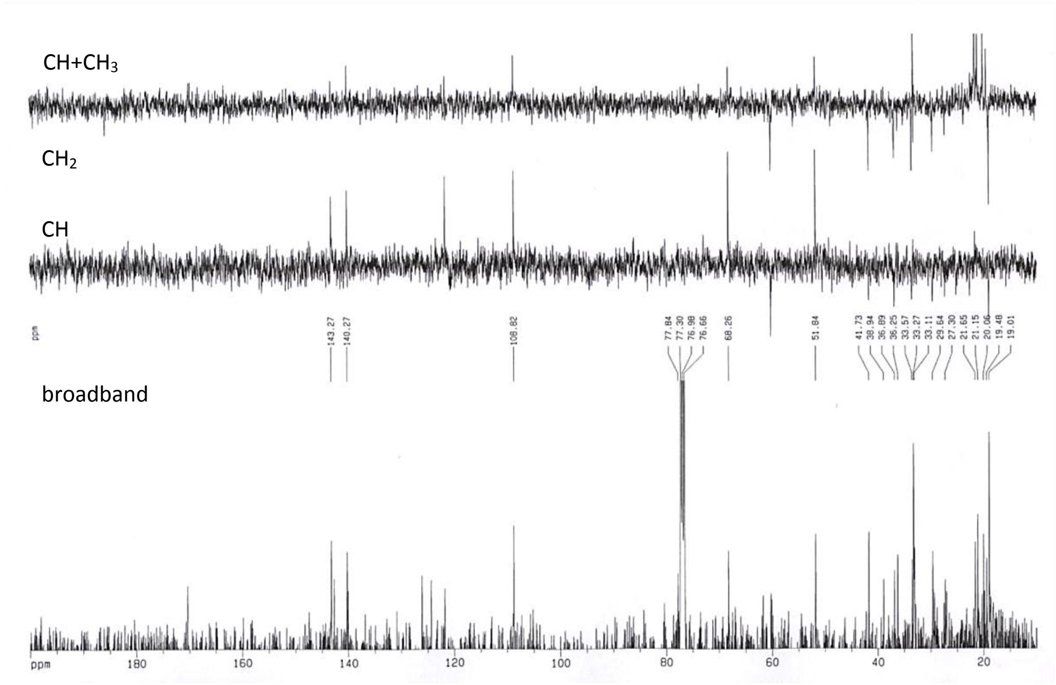

Figure S13. ^{1}H-NMR $CDCl_3$ and ^{13}C-NMR $CDCl_3$.

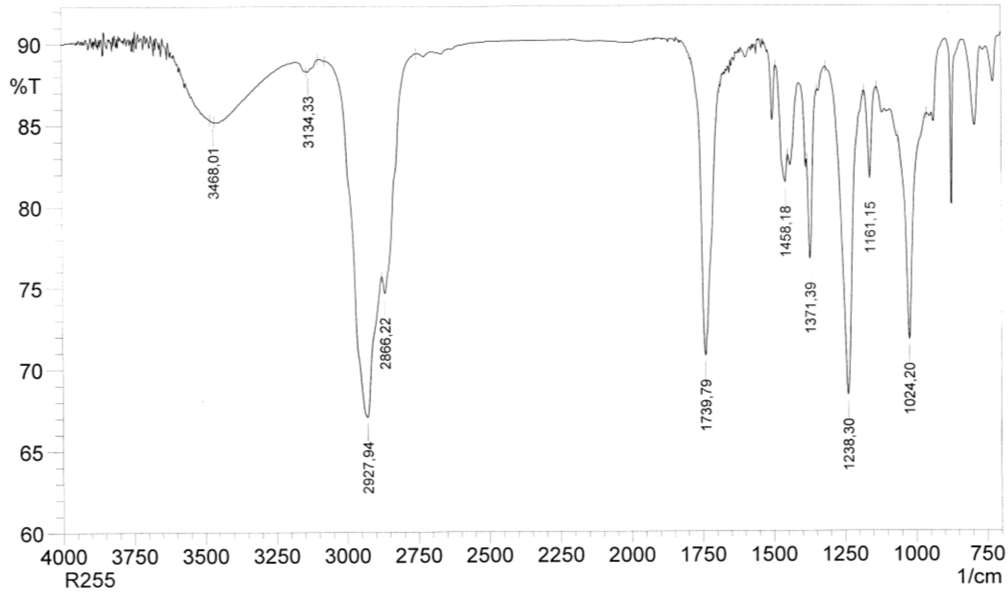

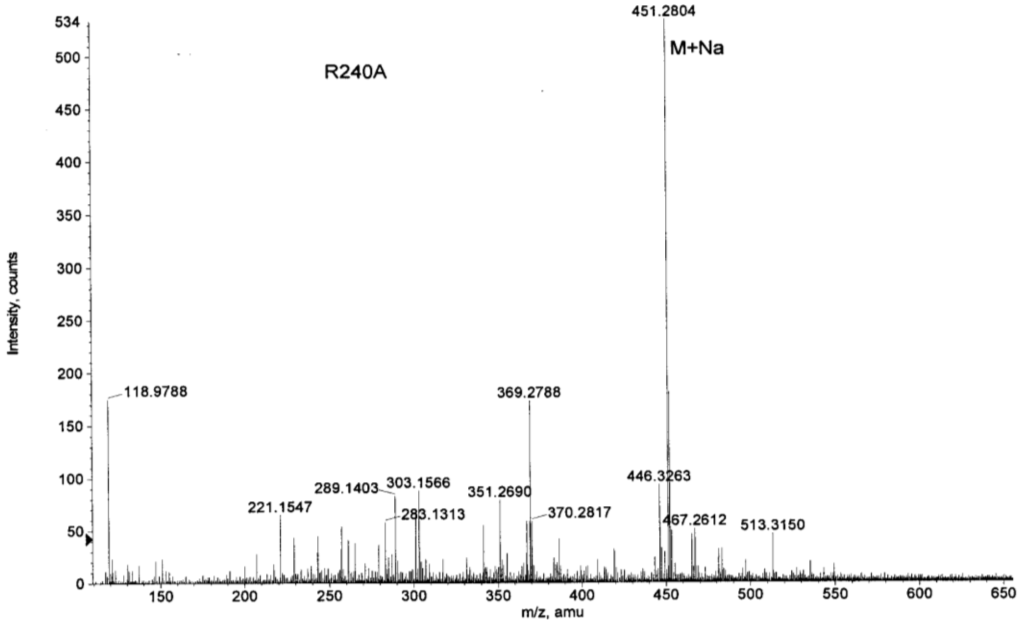

Formula	CalculatedMass	mDaError	ppmError	RDB
C27 H40 O4 Na	451.281881	-1.48128	-3.28239	7.5
C29 H39 O4	451.284287	-3.88654	-8.61224	10.5

Figure S14. IR and HRMS of **22a/22b**.

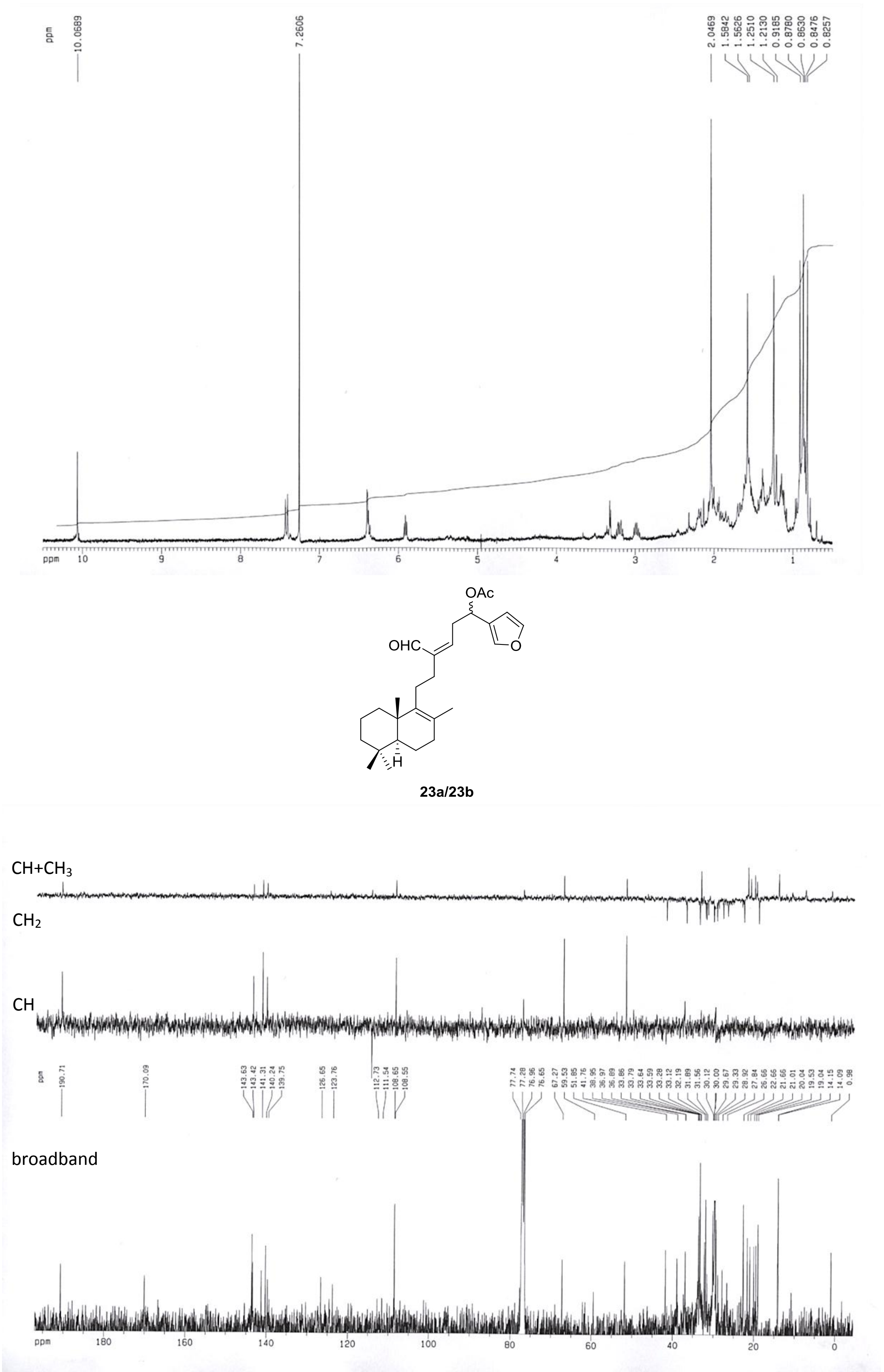

Figure S15. ^{1}H-NMR CDCl$_3$ and ^{13}C-NMR CDCl$_3$.

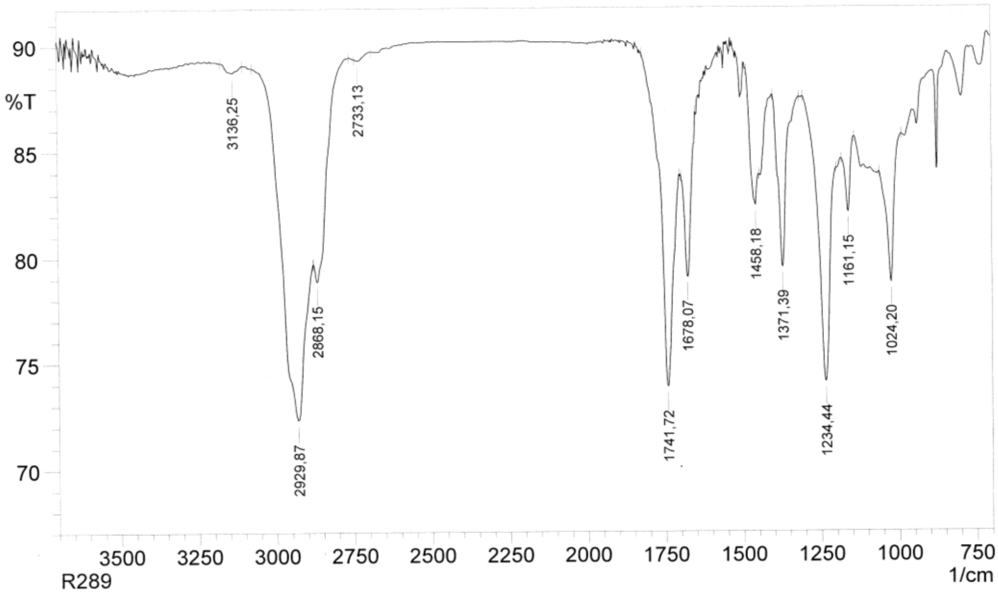

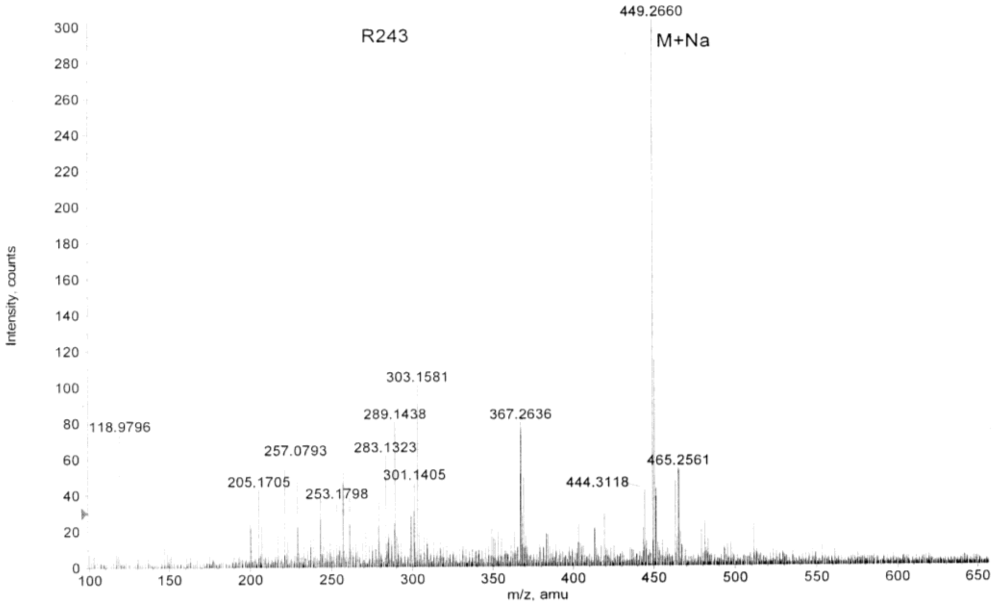

Formula	CalculatedMass	mDaError	ppmError	RDB
C27 H38 O4 Na	449.266231	-0.2312	-0.514617	8.5
C29 H37 O4	449.268636	-2.63646	-5.868365	11.5

Figure S16. IR and HRMS of **23a/23b**.

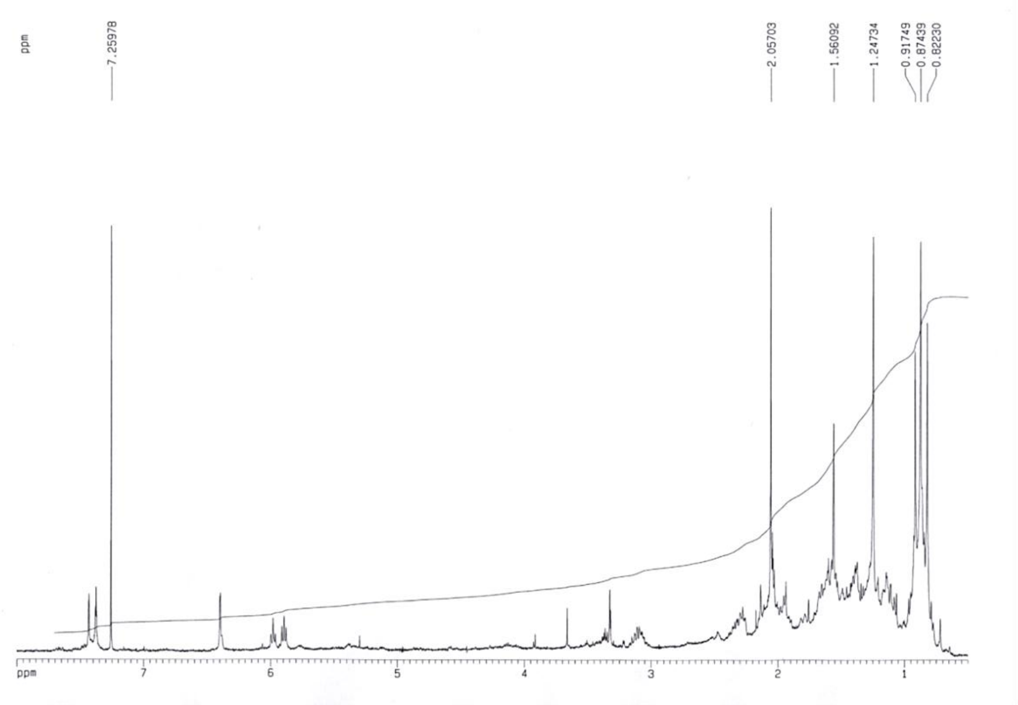

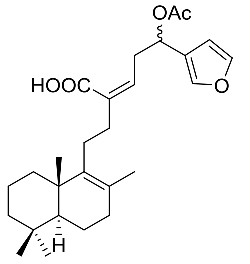

24a/24b

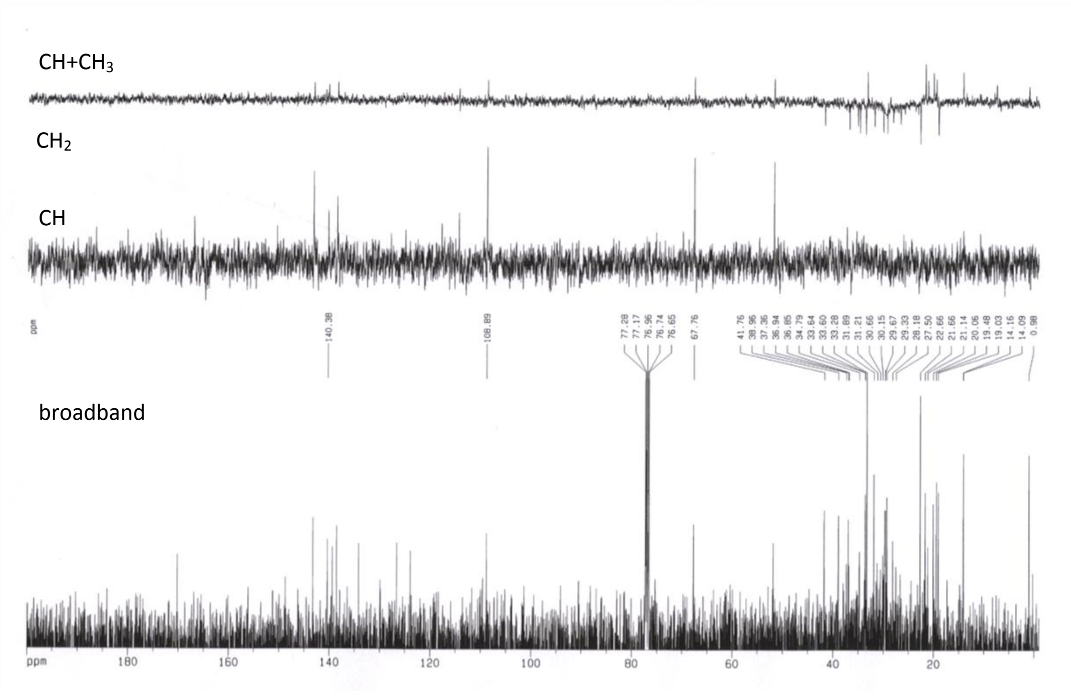

Figure S17. ^{1}H-NMR $CDCl_3$ and ^{13}C-NMR $CDCl_3$.

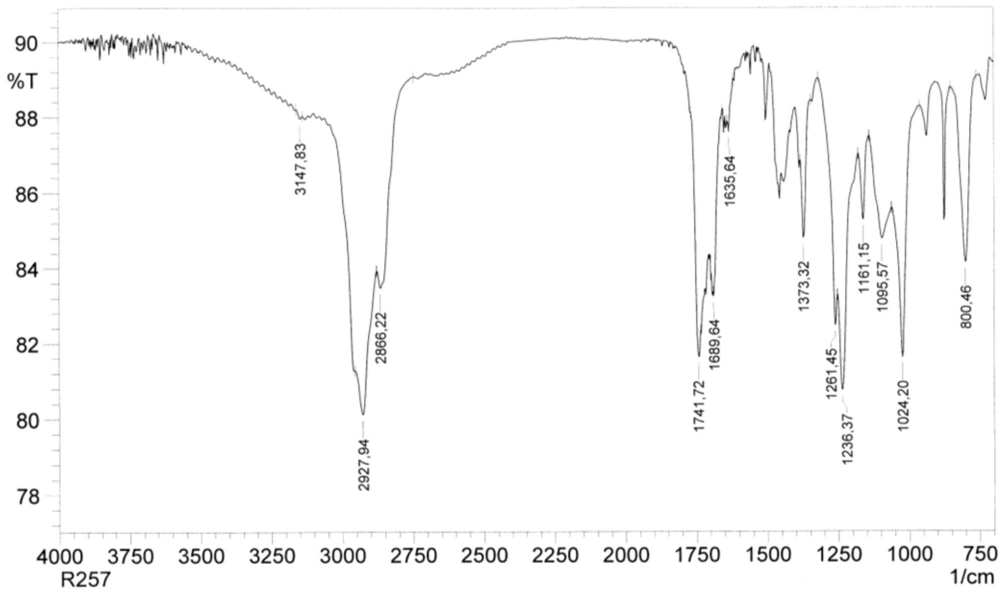

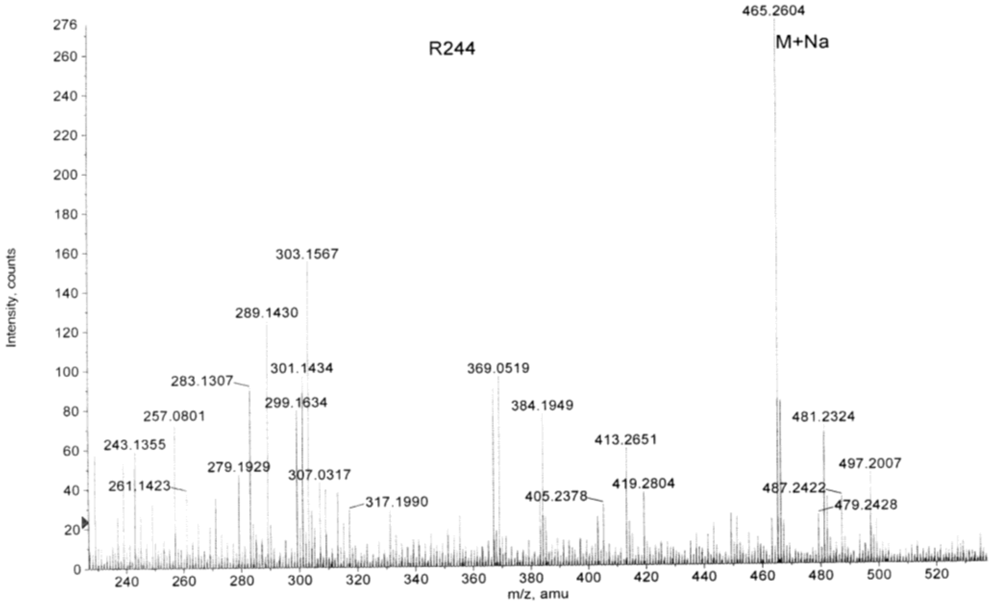

Formula	CalculatedMass	mDaError	ppmError	RDB
C25 H33 N6 O3	465.260866	-0.465708	-1.000961	12.5
C27 H38 O5 Na	465.261146	-0.74584	-1.603057	8.5
C24 H37 N2 O7	465.259528	0.871604	1.873366	7.5
C23 H34 N6 O3 Na	465.25846	1.939552	4.16874	9.5
C28 H34 N4 O Na	465.262483	-2.083152	-4.477384	13.5
C13 H37 N8 O10	465.262716	-2.316364	-4.978634	-0.5
C29 H37 O5	465.263551	-3.1511	-6.772758	11.5
C22 H38 N2 O7 Na	465.257123	3.276864	7.043067	4.5
C20 H33 N8 O5	465.256843	3.556996	7.645163	8.5
C16 H38 N6 O8 Na	465.264334	-3.933808	-8.455057	0.5
C30 H33 N4 O	465.264888	-4.488412	-9.647085	16.5

Figure S18. IR and HRMS of **24a/24b**.

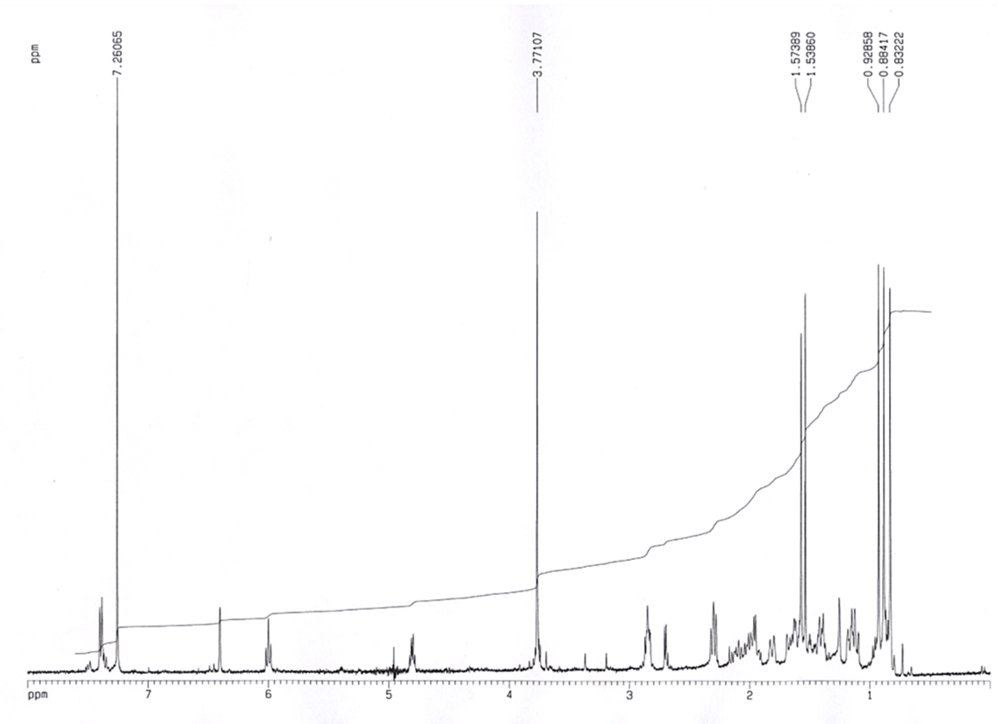

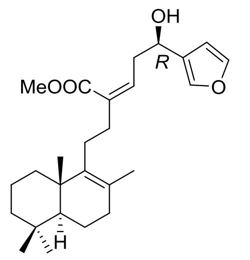

27

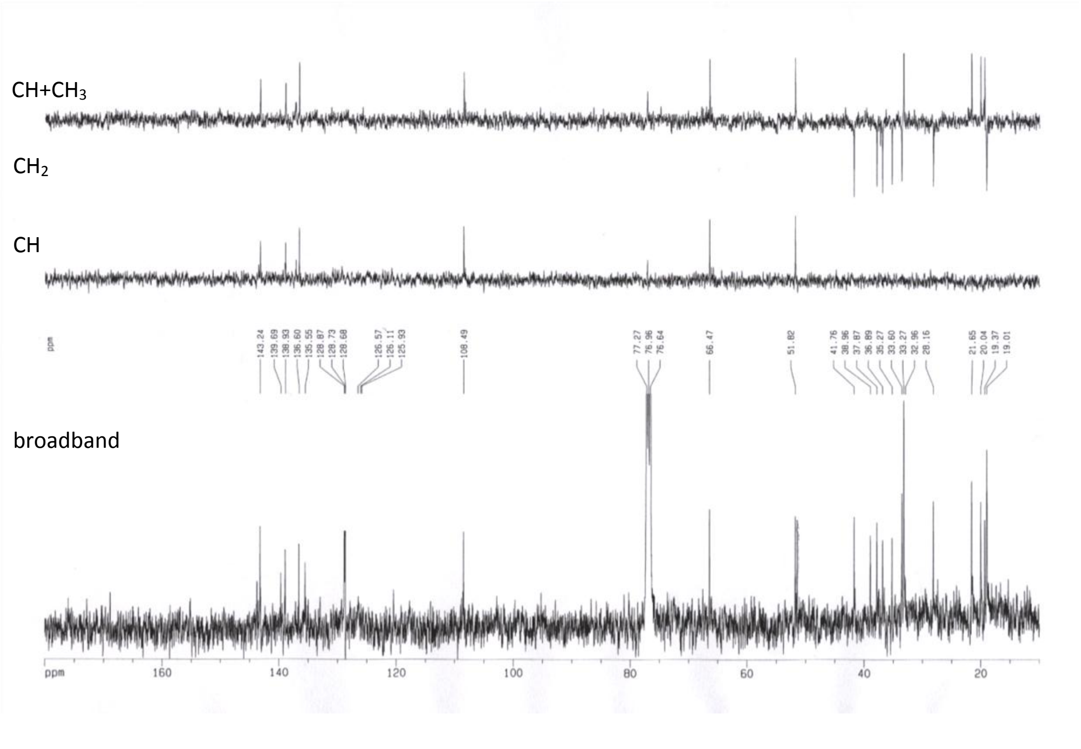

Figure S19. ^{1}H-NMR $CDCl_3$ and ^{13}C-NMR $CDCl_3$.

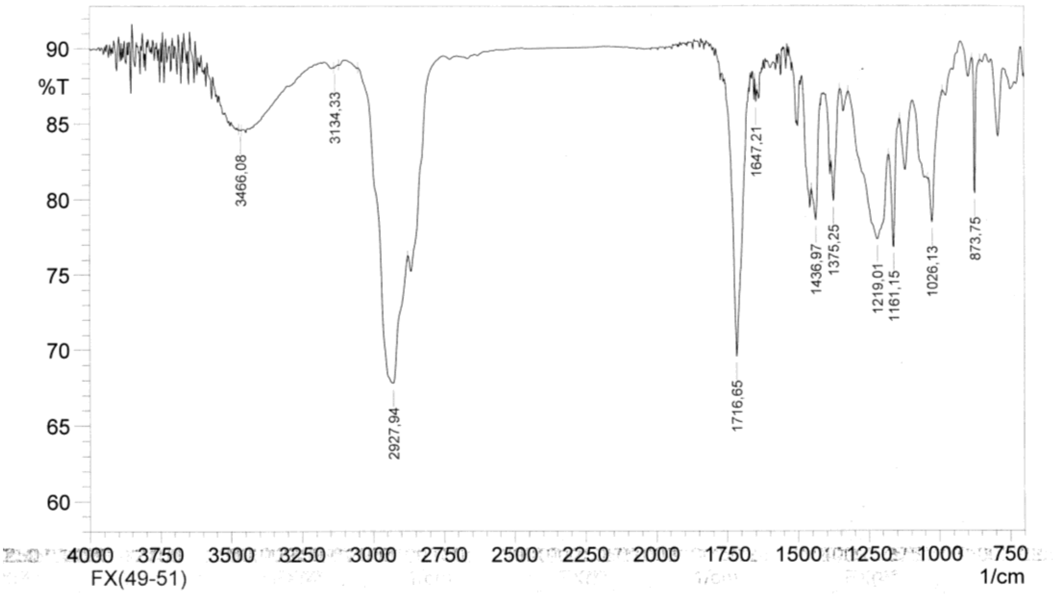

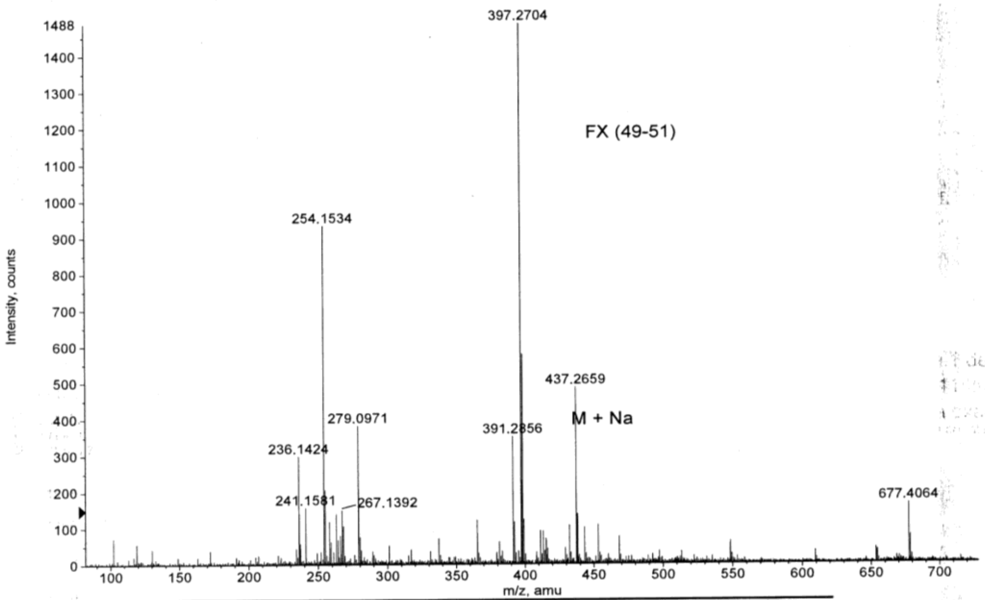

Formula	CalculatedMass	mDaError	ppmError	RDB
C26 H38 O4 Na	437.266231	-0.3312	-0.757433	7.5
C21 H39 N4 O2 Na Cl	437.265376	0.524279	1.198992	3.5
C22 H42 O6 Cl	437.266444	-0.543669	-1.243336	1.5
C23 H37 N2 O6	437.264614	1.286244	2.941557	6.5
C27 H34 N4 Na	437.267569	-1.668512	-3.815779	12.5
C23 H38 N4 O2 Cl	437.267781	-1.880981	-4.301682	6.5
C28 H37 O4	437.268636	-2.73646	-6.258106	10.5
C26 H39 N2 Na Cl	437.269398	-3.498425	-8.000671	7.5
C21 H38 N2 O6 Na	437.262208	3.691504	8.44223	3.5
C22 H43 N2 O2 Cl2	437.269611	-3.710894	-8.486574	1.5
C29 H33 N4	437.269974	-4.073772	-9.316452	15.5

Figure S20. IR and HRMS of **27**.

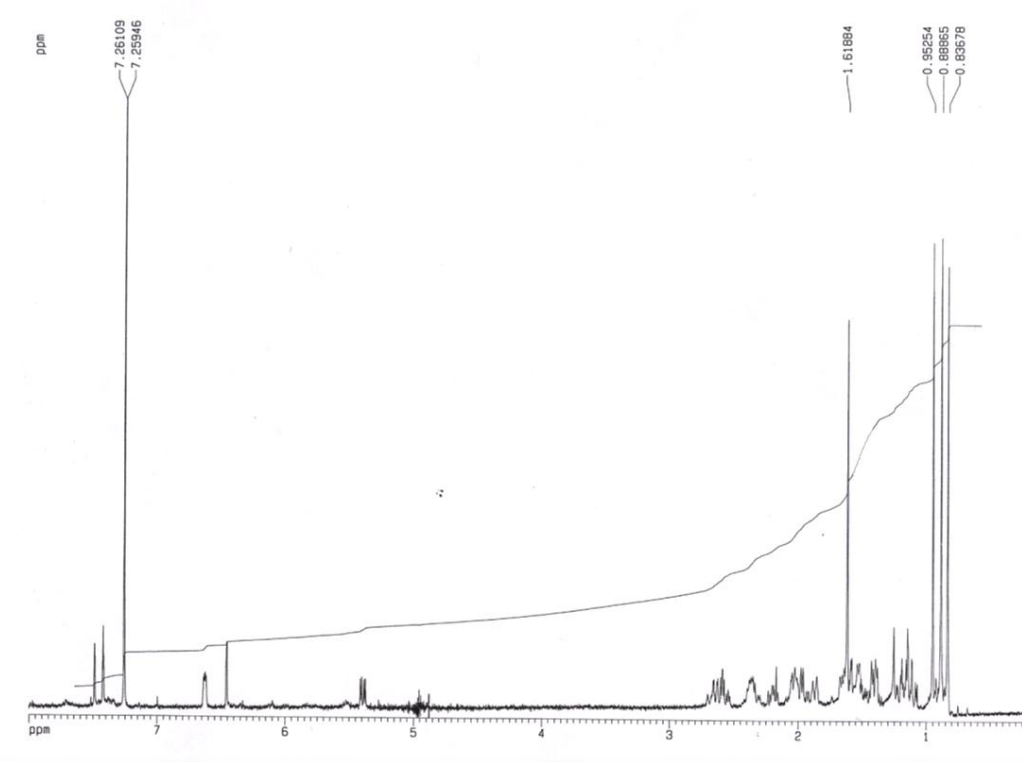

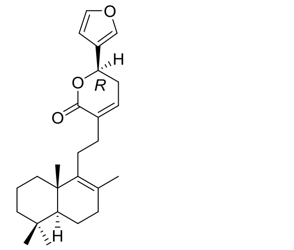

28

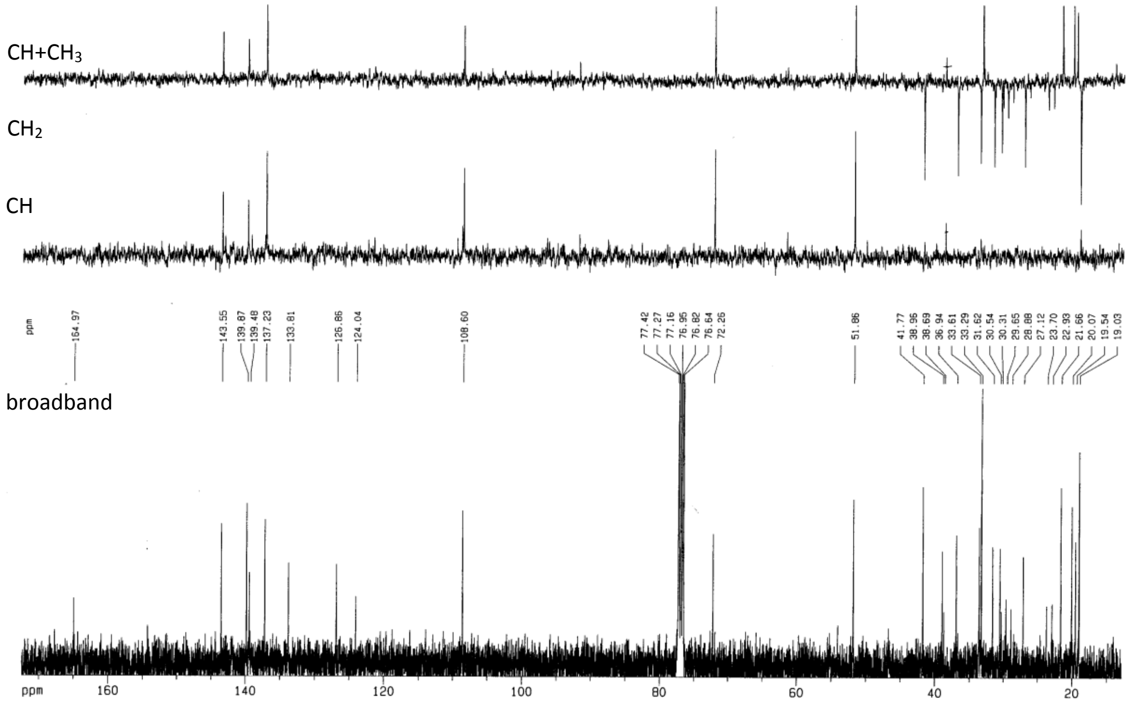

Figure S21. ^{1}H-NMR $CDCl_3$ and ^{13}C-NMR $CDCl_3$.

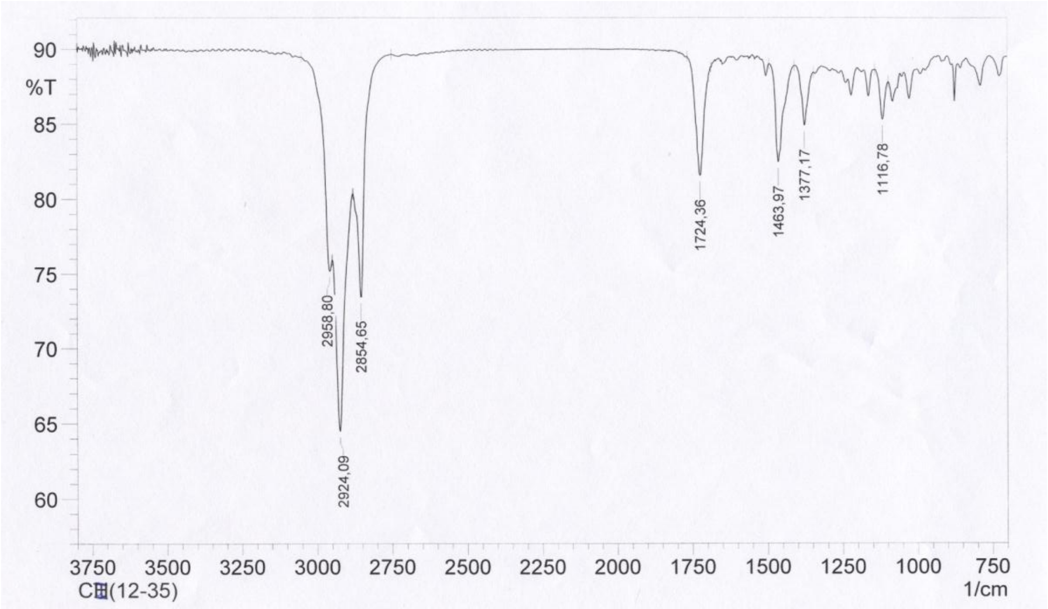

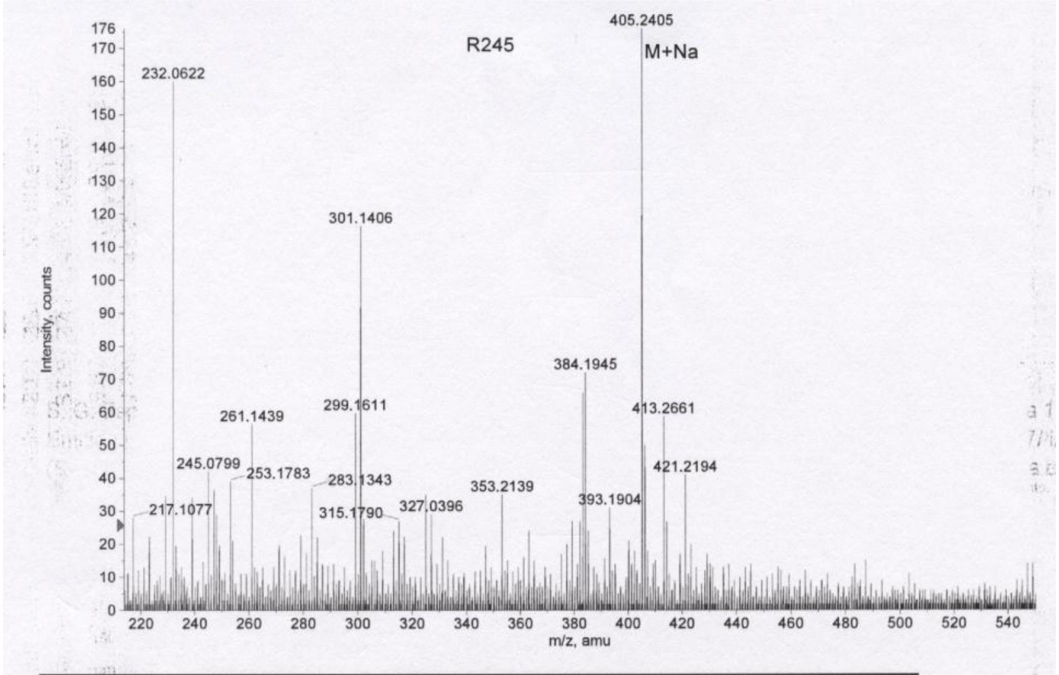

Formula	CalculatedMass	mDaError	ppmError	RDB
C25 H34 O3 Na	405.240016	0.4836	1.193364	8.5

Figure S22. IR and HRMS of **28**.

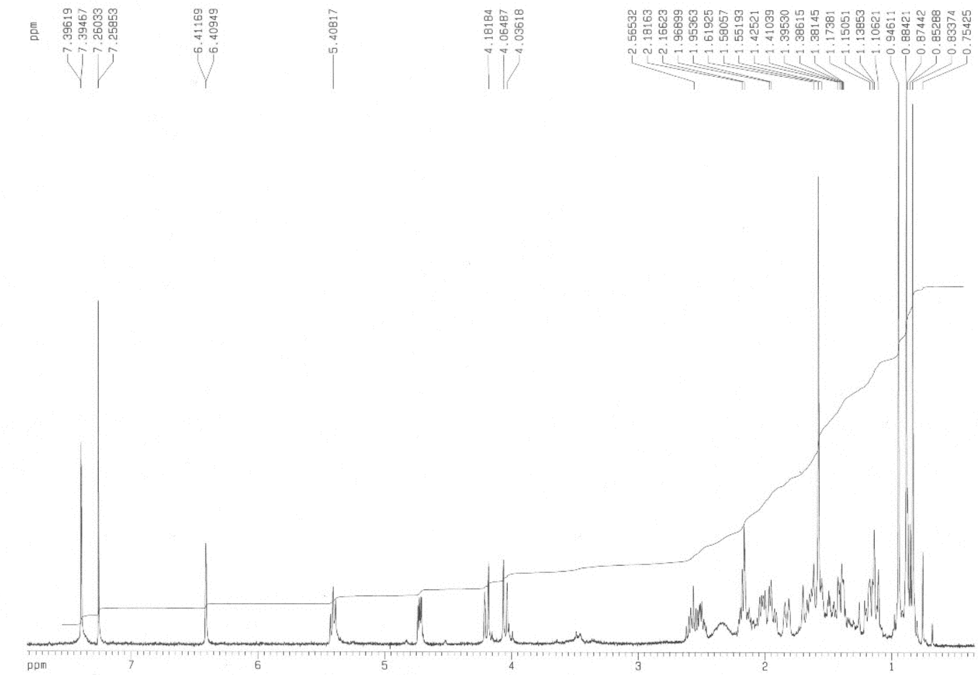

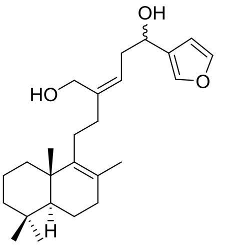

29/29b

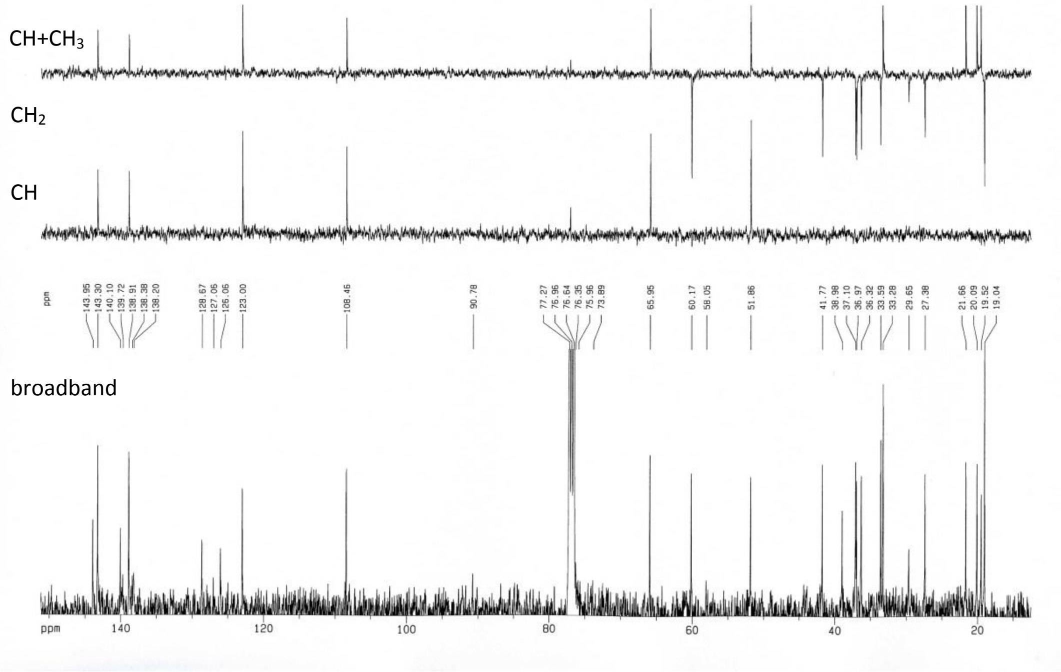

Figure S23. ^{1}H-NMR CDCl3 and ^{13}C-NMR CDCl3.

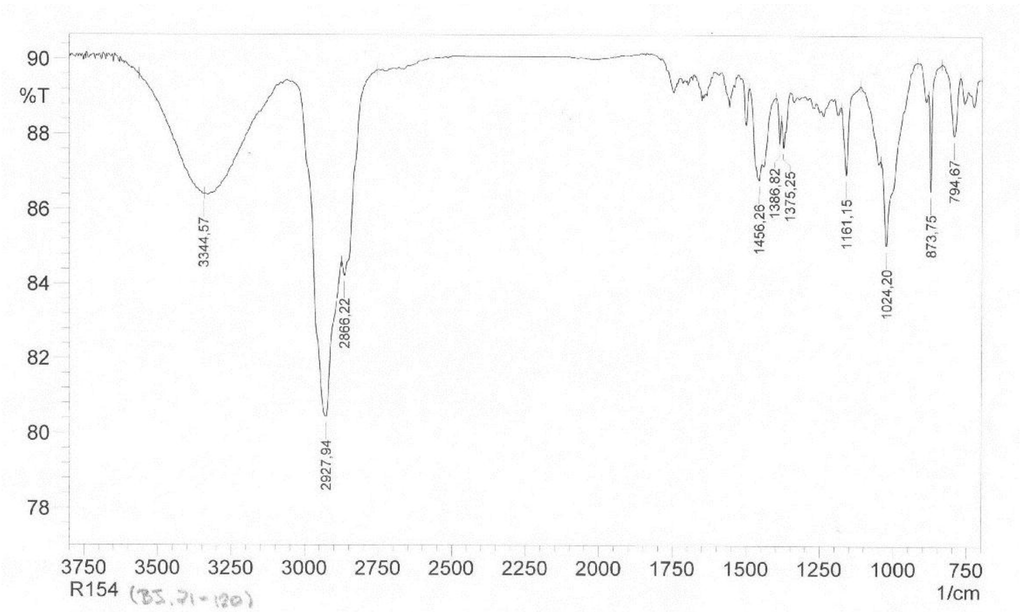

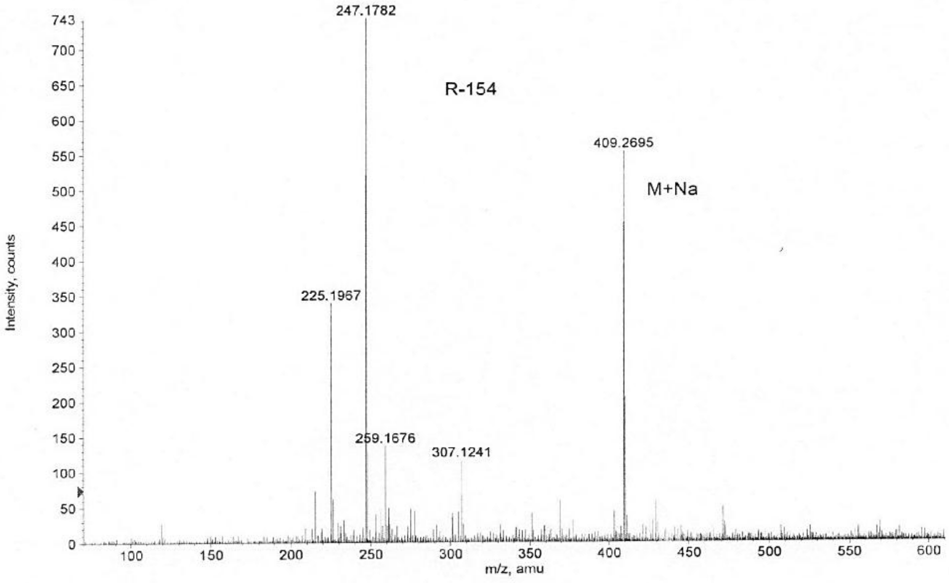

Formula	CalculatedMass	mDaError	ppmError	RDB
C22 H37 N2 O5	409.269699	-0.199116	-0.486515	5.5
C21 H34 N6 O Na	409.268631	0.868832	2.122882	7.5
C23 H33 N6 O	409.271036	-1.536428	-3.754069	10.5
C25 H38 O3 Na	409.271317	-1.81656	-4.438536	6.5
[illegible]	[illegible]	[illegible]	[illegible]	[illegible]

Figure S24. IR and HRMS of **29/29b**.

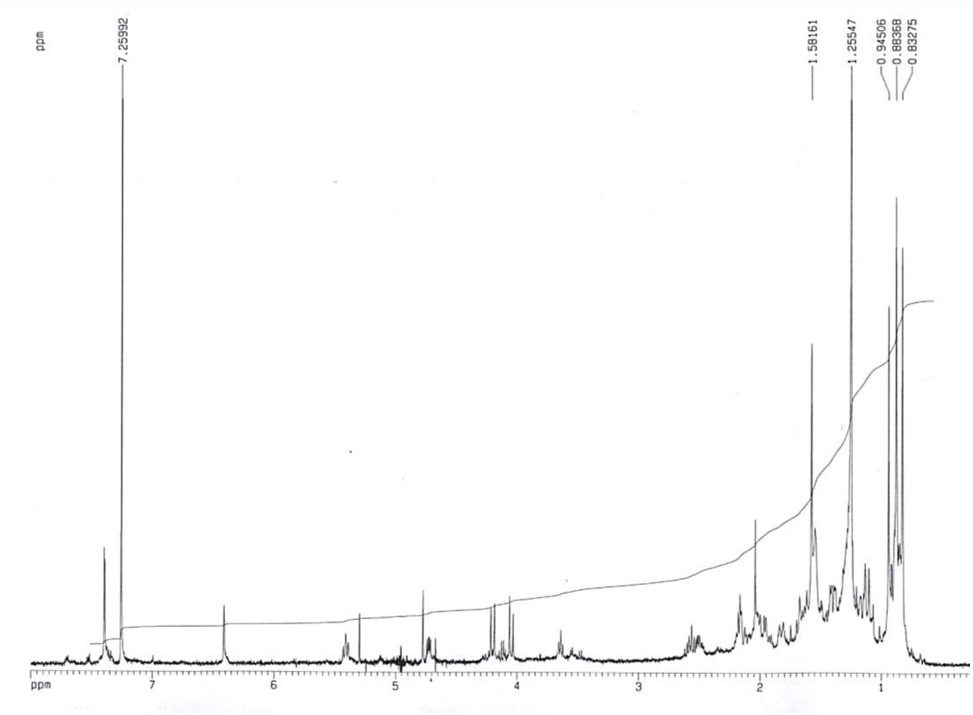

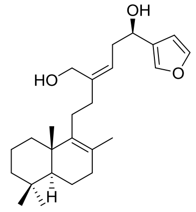

29

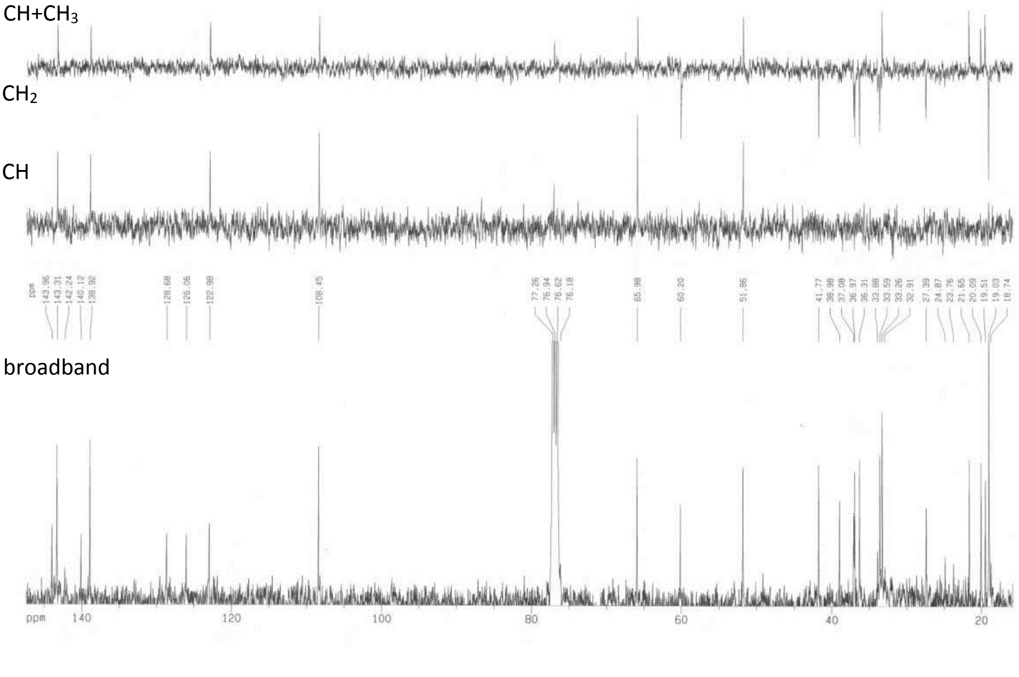

Figure S25. ^{1}H-NMR $CDCl_3$ and ^{13}C-NMR $CDCl_3$.

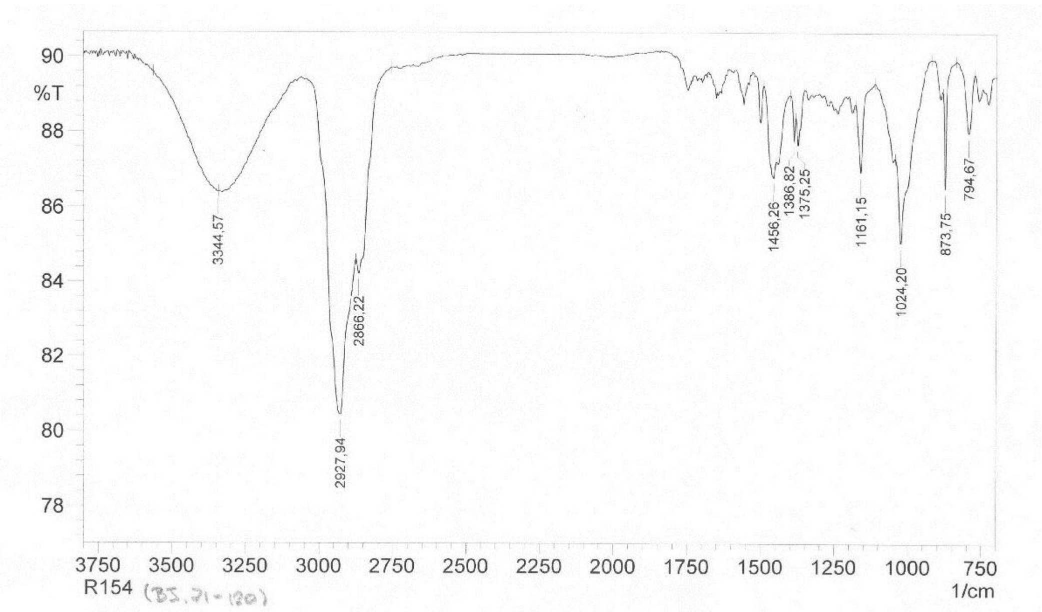

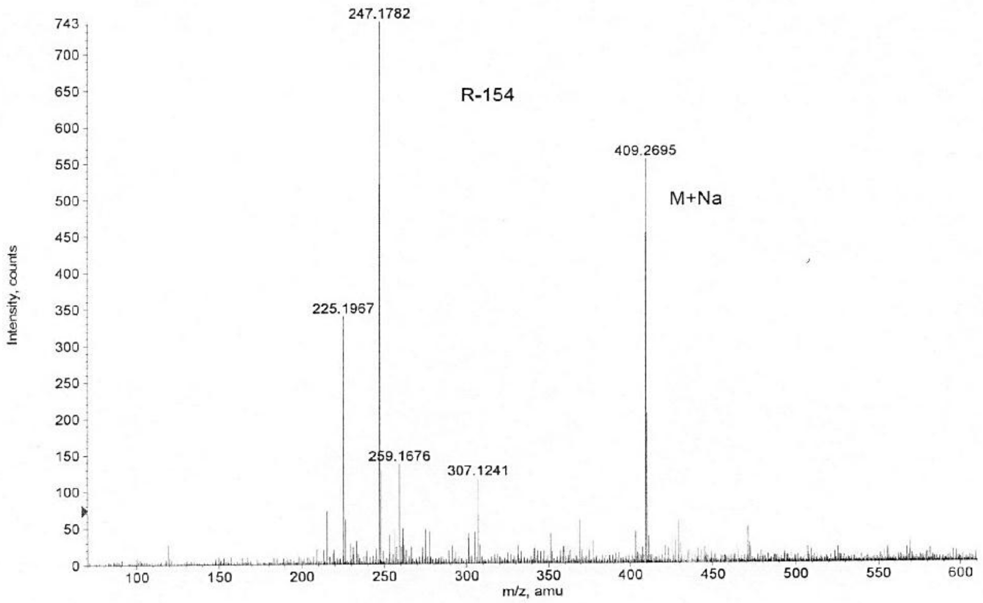

Formula	CalculatedMass	mDaError	ppmError	RDB
C22 H37 N2 O5	409.269699	-0.199116	-0.486515	5.5
C21 H34 N6 O Na	409.268631	0.868832	2.122882	7.5
C23 H33 N6 O	409.271036	-1.536428	-3.754069	10.5
C25 H38 O3 Na	409.271317	-1.81656	-4.438536	6.5
[illegible]	[illegible]	[illegible]	[illegible]	[illegible]

Figure S26. IR and HRMS of **29**.

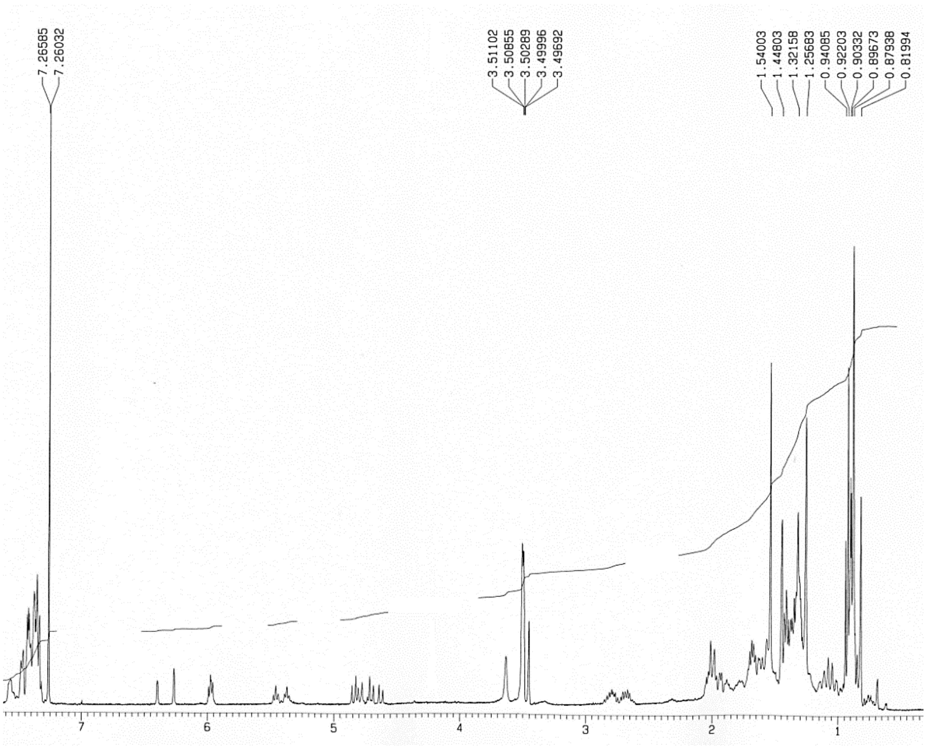

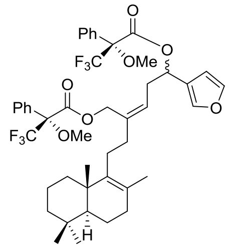

31a/31b

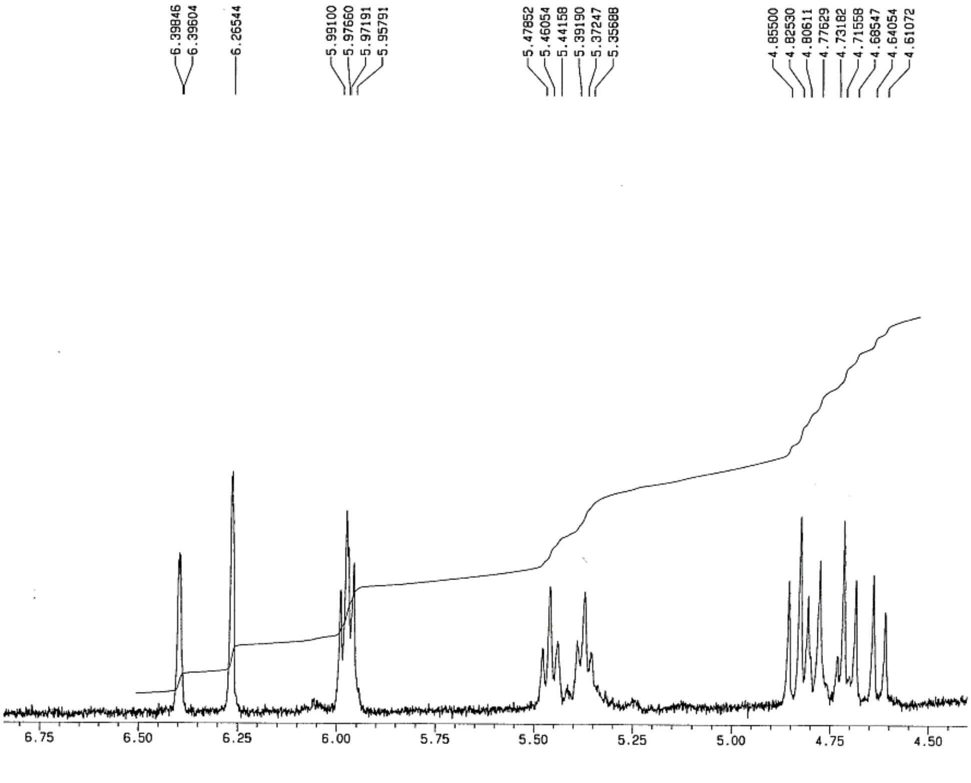

Figure S27. ^{1}H-NMR $CDCl_3$ and expansion of ^{1}H-NMR $CDCl_3$.

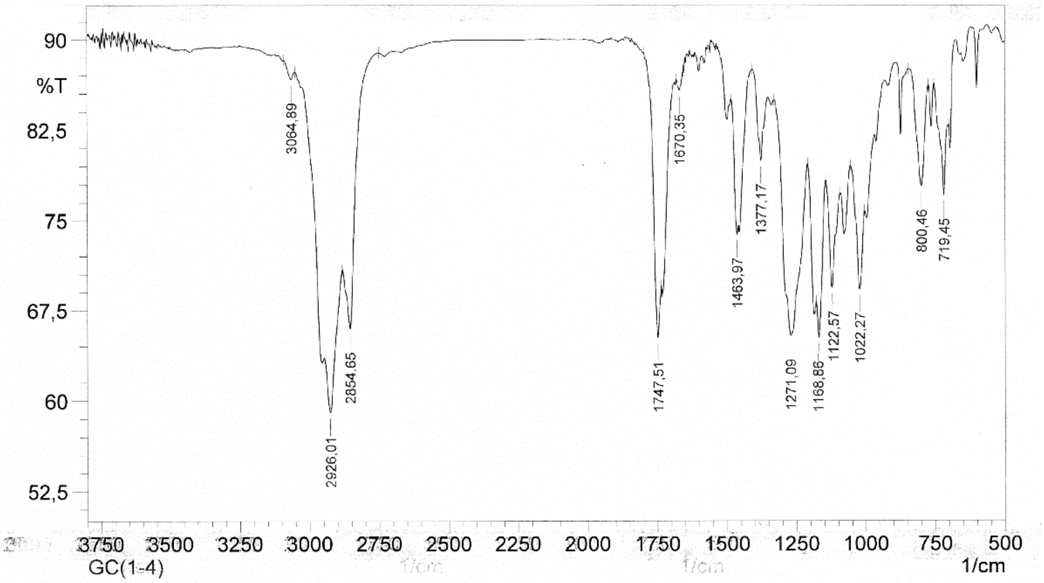

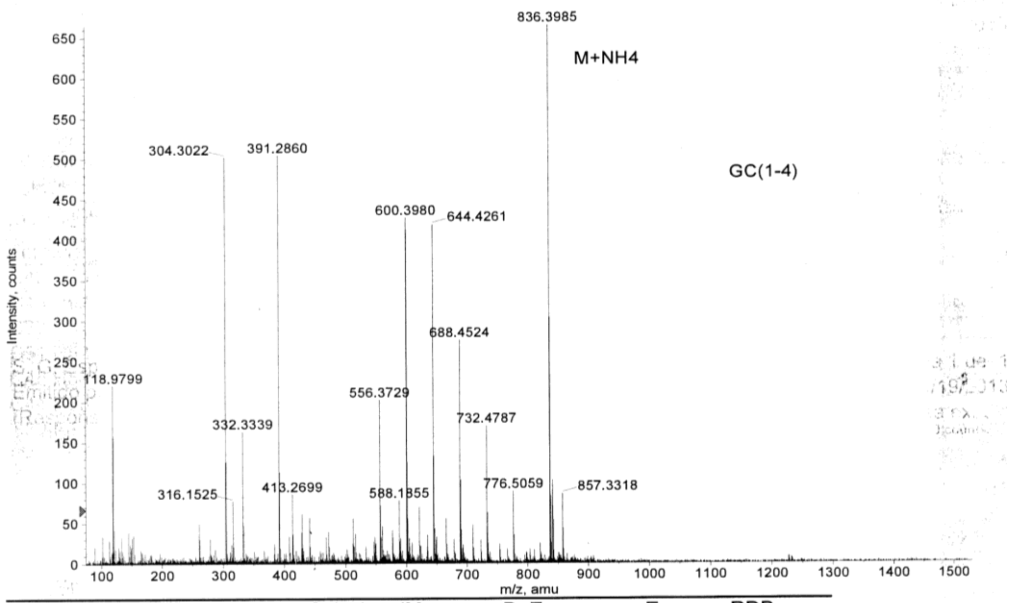

Formula	CalculatedMass	mDaError	ppmError	RDB
C49 H53 N3 O F6 Na	836.398504	-0.004404	-0.005265	21.5
C46 H52 N5 O3 F6	836.396887	1.61304	1.928553	20.5
C37 H57 N5 O8 F6 Na	836.400355	-1.85506	-2.217913	8.5
C51 H52 N3 O F6	836.40091	-2.409664	-2.880998	24.5
C45 H56 N O7 F6	836.39555	2.950352	3.527445	15.5
C44 H53 N5 O3 F6 Na	836.394482	4.0183	4.804286	17.5
C39 H56 N5 O8 F6	836.40276	-4.26032	-5.093645	11.5
C43 H57 N O7 F6 Na	836.393144	5.355612	6.403178	12.5
C42 H57 N3 O6 F6 Na	836.404378	-5.877764	-7.027464	12.5
C40 H56 N3 O9 F6	836.391527	6.973056	8.336997	11.5
C44 H56 N3 O6 F6	836.406783	-8.283024	-9.903196	15.5

Figure S28. IR and HRMS of **31a/31b**.

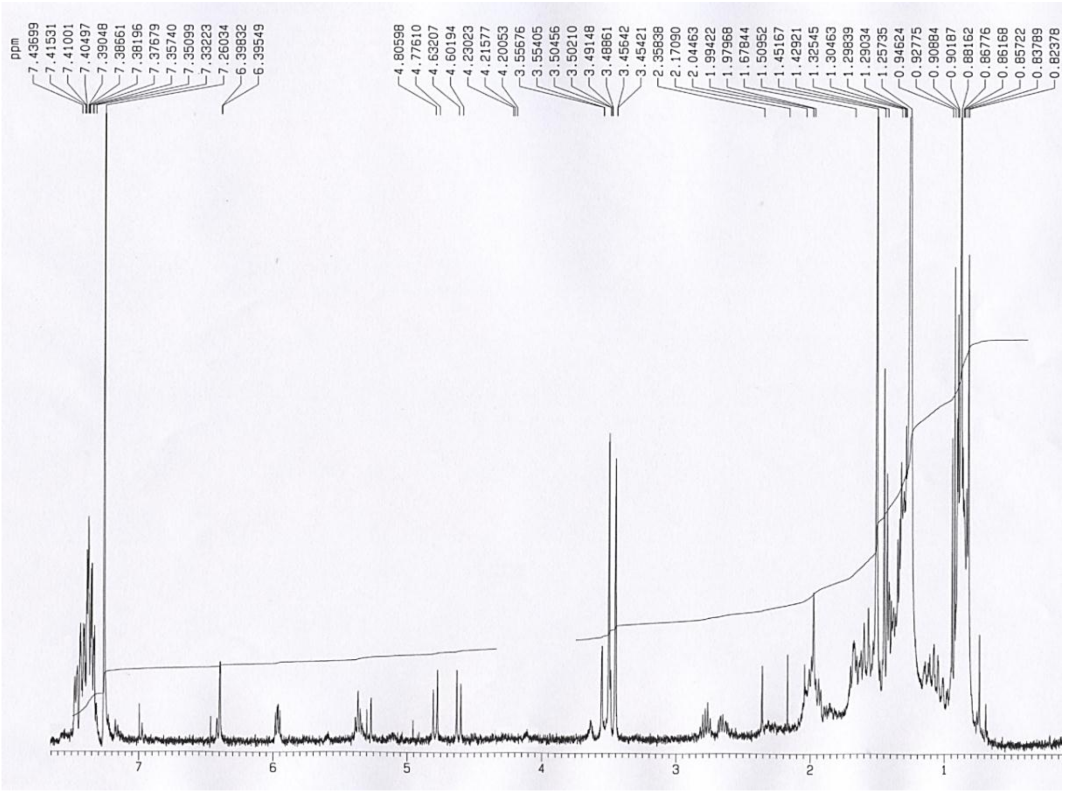

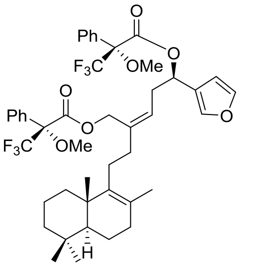

31a

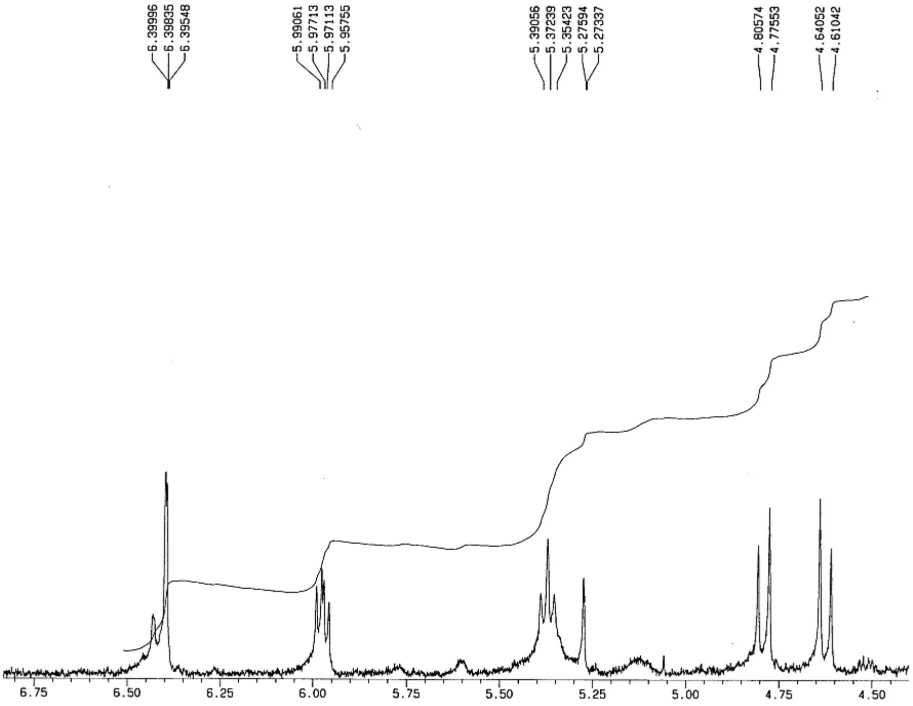

Figure S29. ^{1}H-NMR $CDCl_3$ and expansion of ^{1}H-NMR $CDCl_3$.

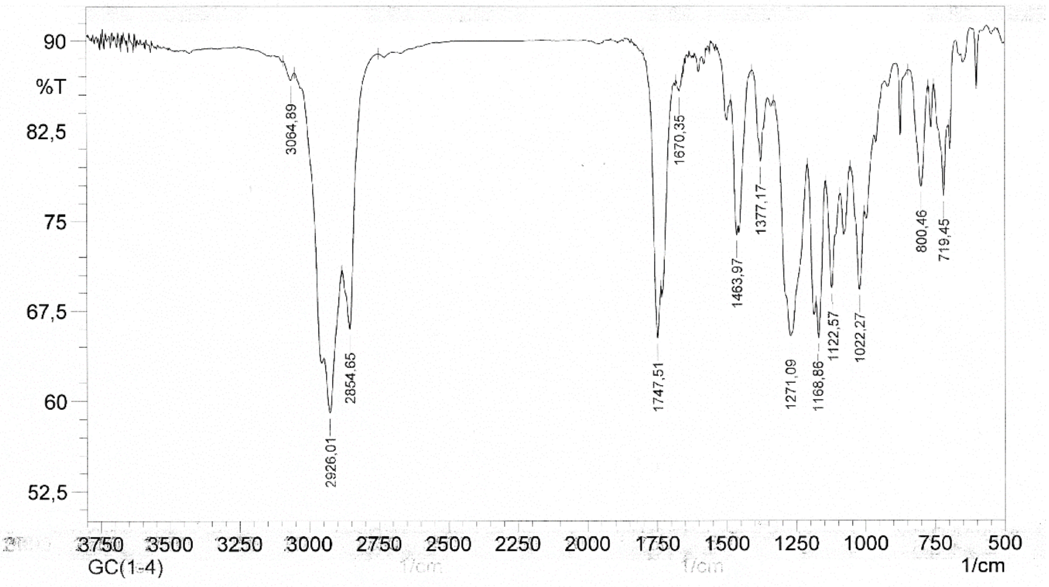

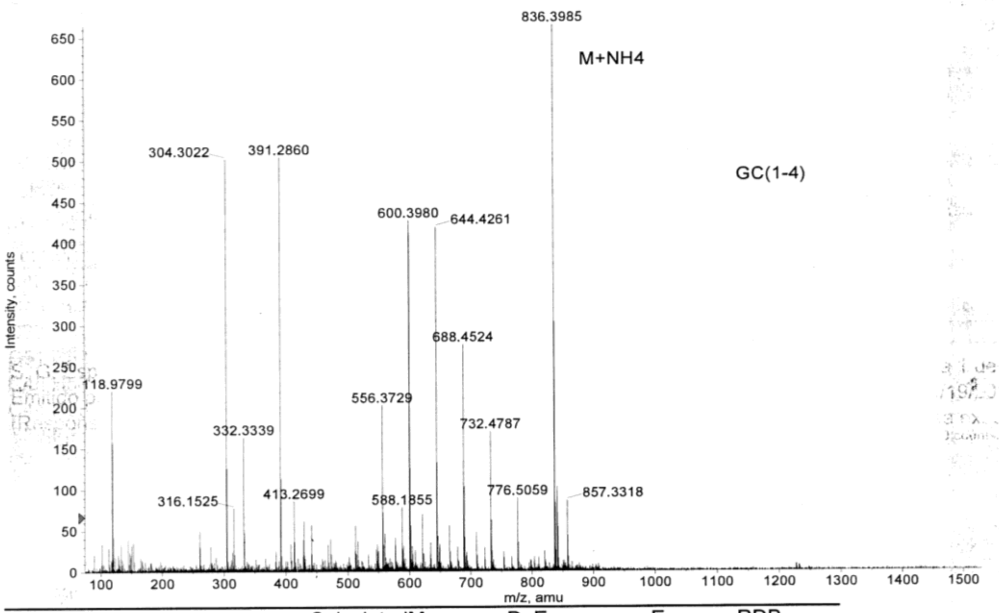

Formula	CalculatedMass	mDaError	ppmError	RDB
C49 H53 N3 O F6 Na	836.398504	-0.004404	-0.005265	21.5
C46 H52 N5 O3 F6	836.396887	1.61304	1.928553	20.5
C37 H57 N5 O8 F6 Na	836.400355	-1.85506	-2.217913	8.5
C51 H52 N3 O F6	836.40091	-2.409664	-2.880998	24.5
C45 H56 N O7 F6	836.39555	2.950352	3.527445	15.5
C44 H53 N5 O3 F6 Na	836.394482	4.0183	4.804286	17.5
C39 H56 N5 O8 F6	836.40276	-4.26032	-5.093645	11.5
C43 H57 N O7 F6 Na	836.393144	5.355612	6.403178	12.5
C42 H57 N3 O6 F6 Na	836.404378	-5.877764	-7.027464	12.5
C40 H56 N3 O9 F6	836.391527	6.973056	8.336997	11.5
C44 H56 N3 O6 F6	836.406783	-8.283024	-9.903196	15.5

Figure S30. IR and HRMS of **31a**.

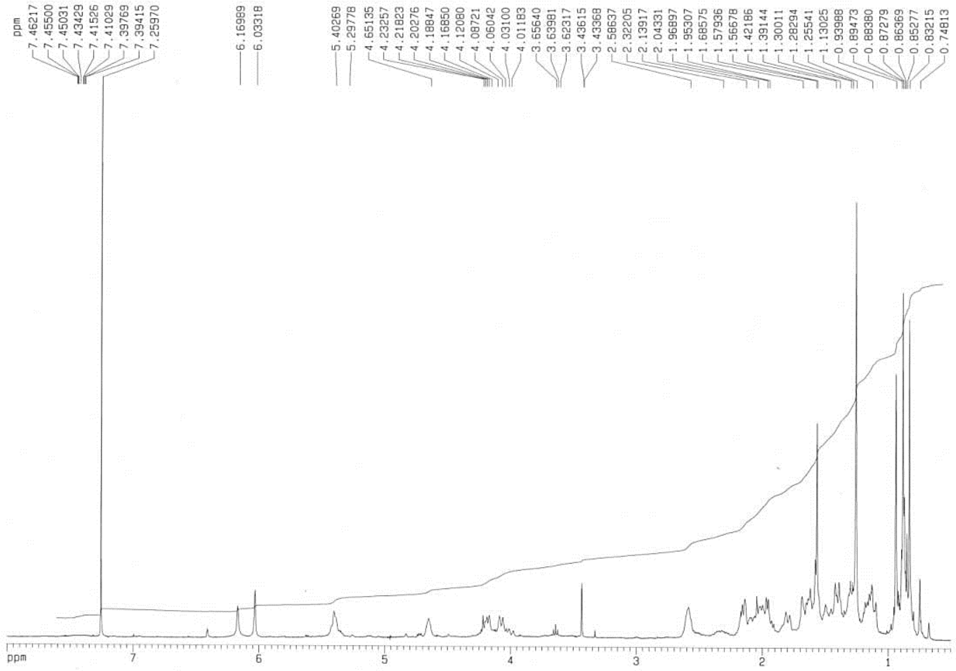

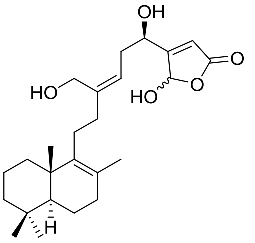

30

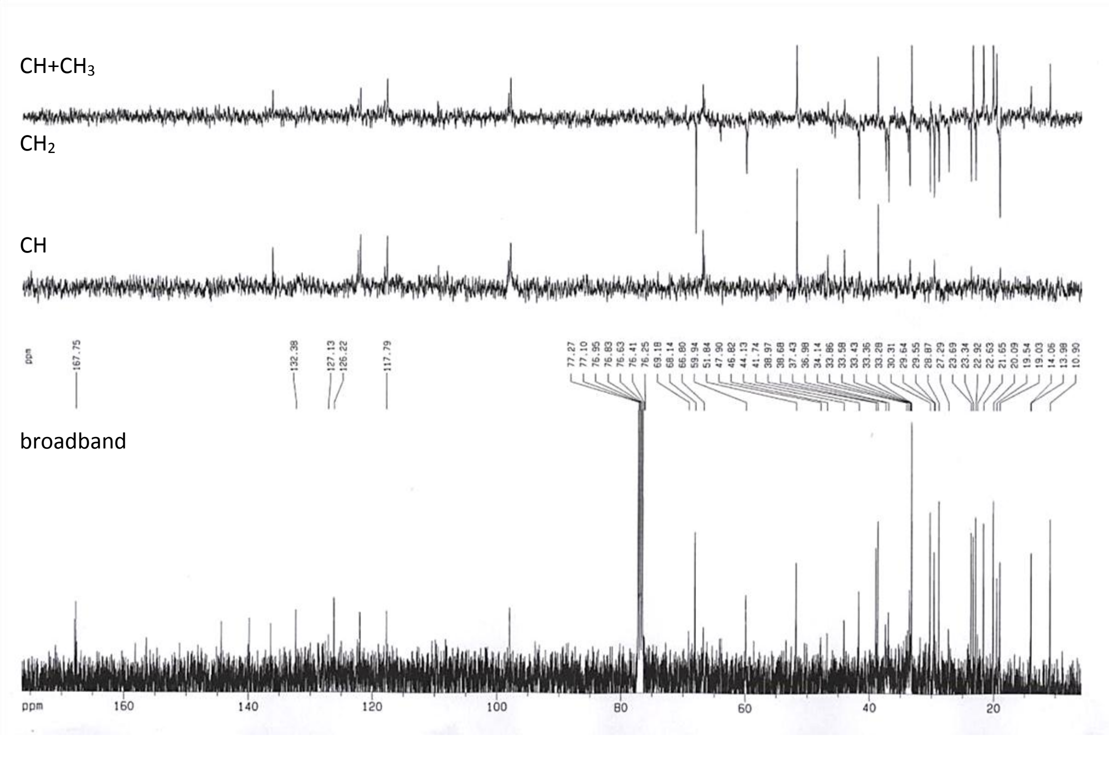

Figure S31. ^{1}H-NMR $CDCl_3$ and ^{13}C-NMR $CDCl_3$.

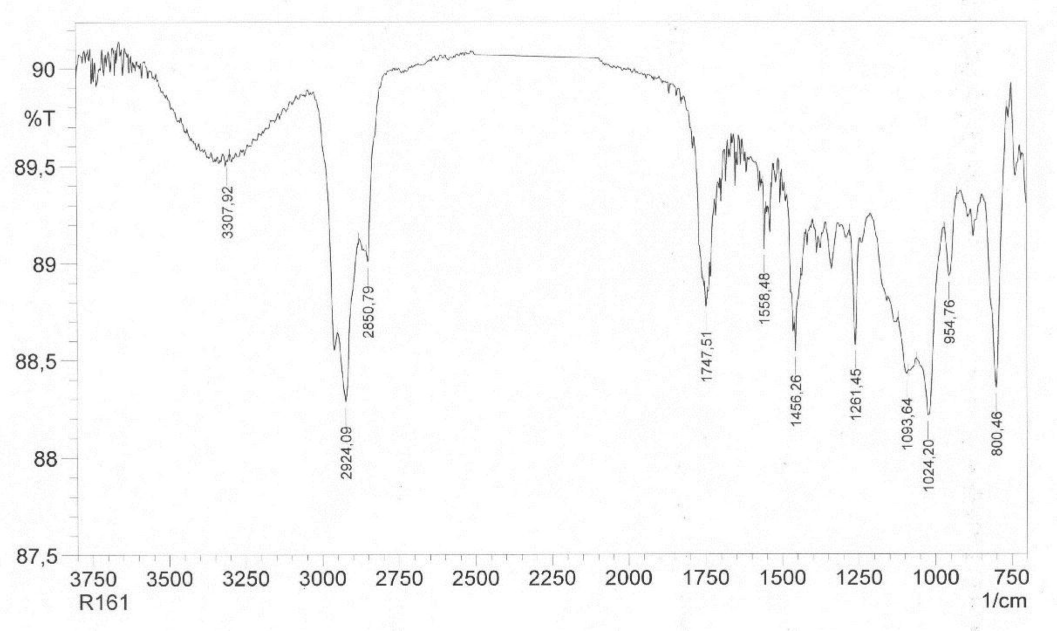

Figure S32. IR of **30**.

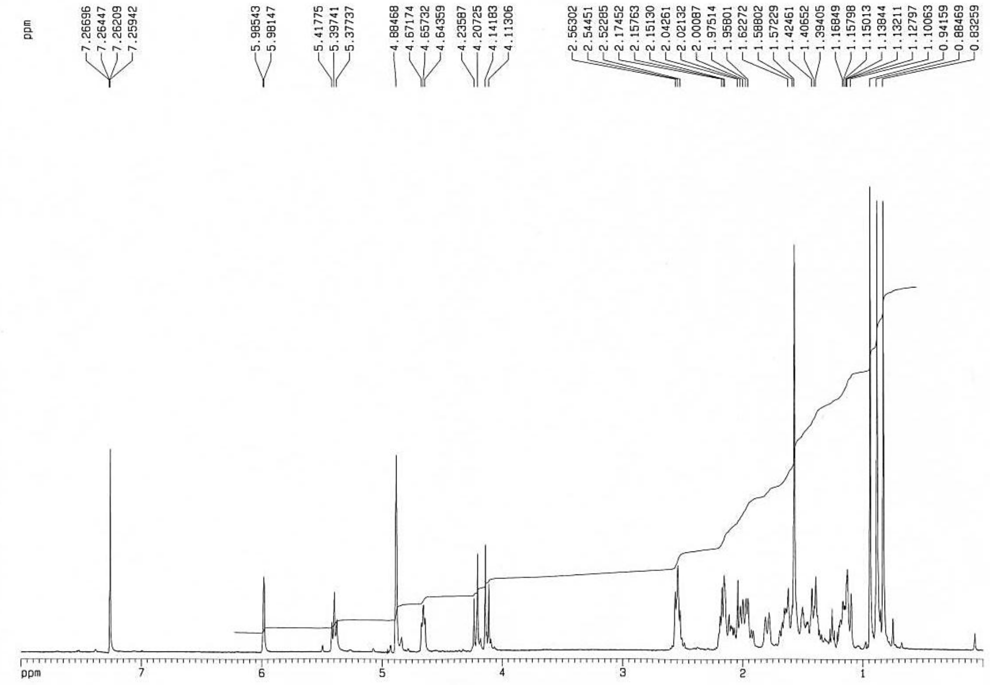

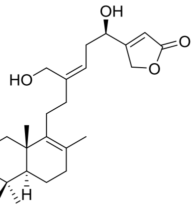

Luffarin I (9)

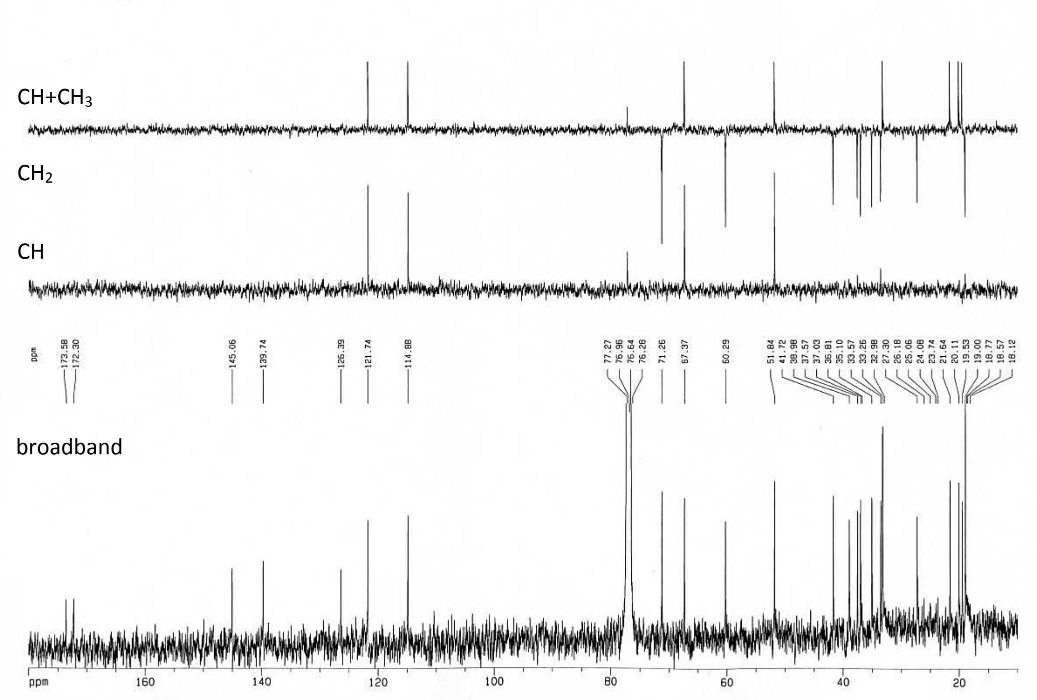

Figure S33. ^{1}H-NMR CDCl3 and ^{13}C-NMR CDCl3.

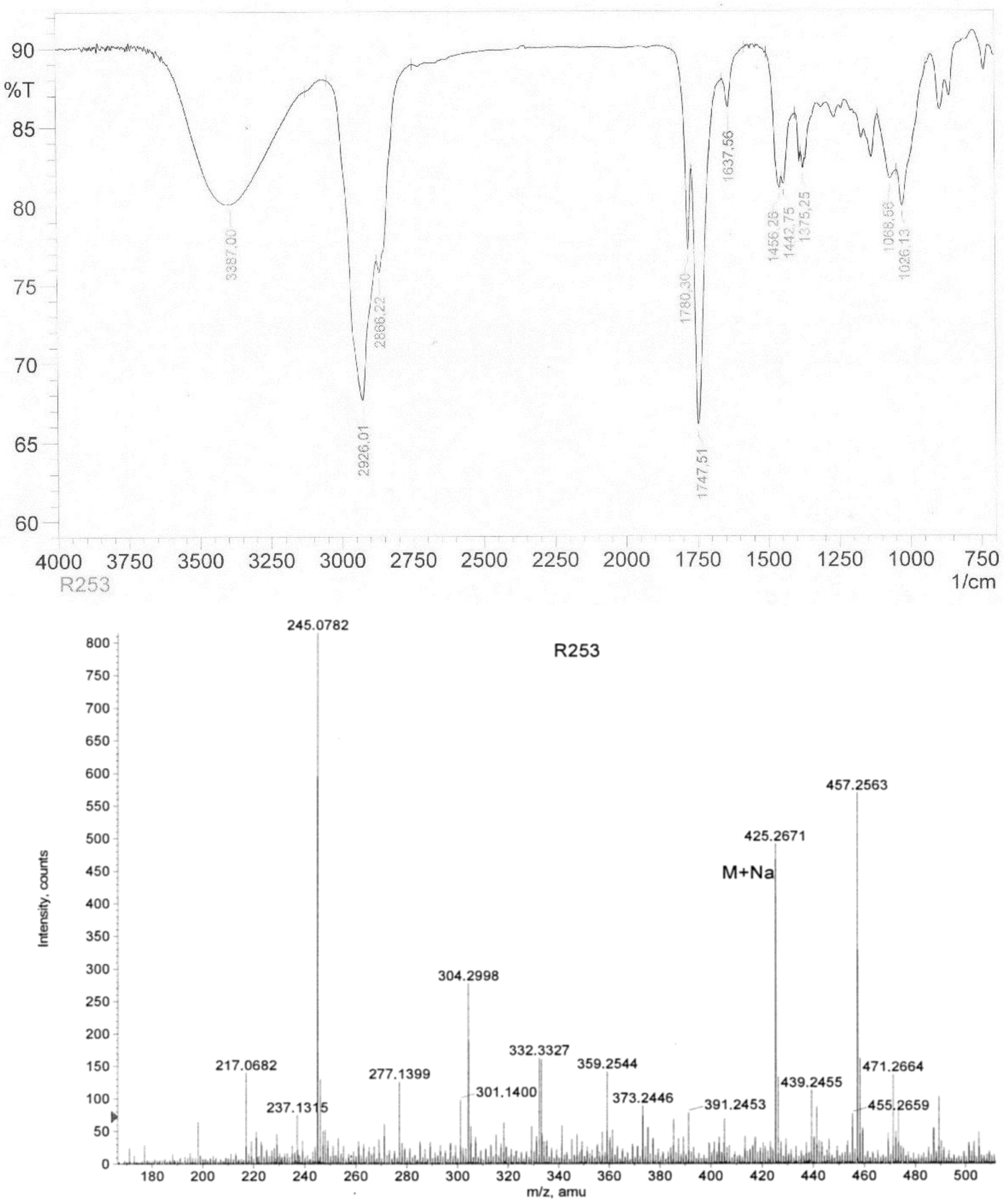

Formula	CalculatedMass	mDaError	ppmError	RDB
C25 H38 O4 Na	425.266231	0.8688	2.042949	6.5
C27 H37 O4	425.268636	-1.53646	-3.612925	9.5

Figure S34. IR and HRMS of **9**.

Reference

1. Ohtani, I.I.; Hotta, K.; Ichikawa, Y.; Isobe, M. Application of modified mosher's method to α-aromatic secondary alcohols. Exception of the rule and conformational analyses. *Chem. Lett.* **1995**, 513–514, doi:10.1246/cl.1995.513.

© 2015 by the authors; licensee MDPI, Basel, Switzerland. This article is an open access article distributed under the terms and conditions of the Creative Commons Attribution license (http://creativecommons.org/licenses/by/4.0/).
